# The global distribution of macroalgal carbon burial in coastal sediments

**DOI:** 10.1038/s41467-026-77215-7

**Published:** 2026-08-31

**Authors:** Dongyang Fan, Carlos M. Duarte, Faming Wang, Oscar Serrano, Dabo Guan, Junyu He, Xiaojing Hua, Yingjie Zhu, Zhangqi Zuo, Karen Filbee-Dexter, Chuancheng Fu, Marianne Holmer, Thomas Wernberg, Ik Kyo Chung, Hatim Albasri, Lei Chen, Yang Liu, Tao Tang, Simai Wang, Xufeng Guo, Xi Xiao

**Affiliations:** 1 Ocean College, Zhejiang University, Zhoushan, China; 2 Marine Science Program, Biological and Environmental Science and Engineering Division (BESE), King Abdullah University of Science and Technology (KAUST), Thuwal, Kingdom of Saudi Arabia; 3 Xiaoliang Research Station for Tropical Coastal Ecosystems, Key Laboratory of Vegetation Restoration and Management of Degraded Ecosystems, and the CAS engineering Laboratory for Ecological Restoration of Island and Coastal Ecosystems, South China Botanical Garden, Chinese Academy of Sciences, Guangzhou, China; 4 Centro de Estudios Avanzados de Blanes, Consejo Superior de Investigaciones Científicas (CEAB-CSIC), Blanes, Spain; 5 Department of Earth System Science, Tsinghua University, Beijing, China; 6 Joint Center for Blue Carbon Research, Ocean Academy, Zhejiang University, Zhoushan, China; 7 Ocean Research Center of Zhoushan, Zhejiang University, Zhoushan, China; 8 School of Biological Sciences and Oceans Institute, University of Western Australia, Perth, WA Australia; 9 Department of Biology, University of Southern Denmark, Odense, Denmark; 10 Department of Ecological Engineering, Pukyong National University, Busan, South Korea; 11 Research Organization for Agriculture and Food, National Research and Innovation Agency, Jakarta, Indonesia; 12 College of Mechanical Engineering, Guizhou University, Guiyang, China

**Keywords:** Carbon cycle, Climate-change mitigation, Carbon cycle, Climate and Earth system modelling

## Abstract

Macroalgae play a pivotal role in the global ocean carbon cycle, yet the spatial distribution of their coastal carbon burial, a long-term sink relevant to national inventories, remains poorly resolved. Here, we present a high-resolution (5-arcminute, ~9.2 km at the equator) global assessment of the Macroalgal Carbon Burial Index (MCBI), a measure of how favorable coastal environments are for burying macroalgal carbon. Synthesizing the most comprehensive dataset of sediment accumulation rates (*n* = 1617), we identify the depositional environment as a critical constraint on burial, acting independently of macroalgal productivity. High-favorability sinks are geographically rare (<1% of global coastlines), clustering in regions such as the Korea Bay and Okhotsk-Sakhalin region. We further identify coastline complexity as an informative predictor of MCBI (R² = 0.59), enabling low-cost identification of burial hotspots. Notably, we identify a mismatch in conservation: existing marine protected areas cover 23.9% of low-burial coastlines but only 17.1% of primary burial hotspots. Our findings map the global pattern of macroalgal carbon burial and provide a spatially explicit basis for its integration into conservation and management.

## Abstract (web summary)

This study mapped global hotspots for macroalgal carbon burial, showing favorable sites for sequestration. Coastline complexity predicts these hotspots, and combining burial favorability with century-scale preservation identifies the most durable blue carbon sinks.

## Introduction

With an estimated global net primary productivity (NPP) of ~1.32 Pg C yr⁻¹^1^, macroalgae rank among the most productive ecosystems, forming habitats widely distributed across the coastal ocean^1^. These significant ‘blue carbon’ habitats, alongside mangroves, seagrass beds, and tidal marshes, play an important role in climate change mitigation and adaptation^2,3^. The extensive practice of macroalgal aquaculture further amplifies these benefits, contributing not only to carbon sequestration^4^, but also to food security, pollution reduction, and coastal protection^5–8^.

Despite the recognition of macroalgae as a major marine primary producer, a critical question regarding its ultimate carbon sink strength remains unresolved. The sequestration pathways are known: (i) burial in coastal sediments and (ii) export to the deep ocean (below 200 m)^9^. The deep ocean carbon sink was recently estimated at 10–170 Tg C yr⁻¹^10^, with a possible portion transforming into refractory dissolved organic carbon supporting long-term storage^11,12^. However, a pivotal limitation persists: while deep ocean export is increasingly mapped at 7.5-arcminute resolution^10^, the spatial distribution of macroalgal carbon burial in coastal sediments remains poorly resolved. Closing this gap is critical because most macroalgal detritus is likely to accumulate nearshore, where it can be buried over long timescales^9,10^. The current global-scale estimate of this nearshore storage flux, approximately 20 Tg C yr⁻¹, or 13.1% of exported macroalgal carbon^9^, is still preliminary, relying heavily on limited observations (*n* = 30) and burial rates from a single mesocosm experiment^13,14^. The challenge is compounded by the inherent complexity of coastal systems, where burial rates vary substantially due to differences in macroalgal NPP, sediment accumulation rate (SAR), detritus transport, and hydrodynamic conditions^4,9,15,16^. Among these, NPP and SAR are the two paramount parameters directly governing carbon deposition^17–19^. Functionally, high SAR serves as a critical physical constraint on sequestration, supporting the co-deposition of macroalgal detritus with mineral sediments, leading to rapid vertical burial, effectively shielding organic matter from remineralization and physical resuspension^20,21^. Yet, existing data-driven models for global SAR lack sufficient data coverage across extensive coastal regions, particularly along the coastlines of Australia and South America, thereby preventing an accurate characterization of the depositional environments that control long-term macroalgal carbon burial^22,23^.

The spatial distribution of macroalgal sedimentary carbon sequestration in coastal sediments is governed by a sequential, biogeochemical framework comprising three primary factors: (i) the initial production of macroalgal-derived detrital particulate organic carbon (POC; herein defined broadly to encompass both fragmented macroalgal particles and larger, stochastically dislodged macroalgal biomass) at the source^10,24^, (ii) the efficiency of adjacent depositional environments as long-term sinks^4^, and (iii) the hydrodynamic transport pathways that connect them^10^. Because macroalgae predominantly colonize hard substrates where in situ sedimentation is negligible^9,25^, their long-term burial relies entirely on this spatial coupling. Therefore, accurately mapping this carbon sink requires a framework that evaluates the capacity of nearby soft-sediment systems to trap detritus exported from proximal hard-substrate habitats. The process begins with high NPP, which supplies the essential organic substrate; an estimated ~71% of macroalgal NPP enters the water column as detritus^10^. This material is then subject to transport, with currents determining its fate. Crucially, approximately 82% of macroalgal POC does not reach the deep ocean but remains within the continental shelf system^10^. Its final sequestration depends on encountering depositional environments with high SAR, which function as effective carbon traps by rapidly burying organic matter and facilitating its transfer into century-scale storage pools. Conversely, in low SAR environments, macroalgal POC remains exposed at the sediment-water interface for extended periods, leading to rapid microbial biodegradation and remineralization, which effectively reduces long-term sequestration^26^. The sustained retention of macroalgal carbon occurs predominantly within these settings, underscoring their role as stable and persistent sinks^27–29^. Consequently, regions that combine high macroalgal productivity with favorable sedimentation dynamics, a synergy that can be amplified through careful selection of the future location of macroalgal aquaculture farms, emerge as hotspots for macroalgal carbon storage^4,19,27,30–32^.

Moreover, delineating the geography of macroalgal carbon burial is pivotal for translating biogeochemical constraints into actionable climate policy. A critical limitation in current carbon accounting is the exclusion of deep-ocean sinks due to the jurisdictional ambiguity of the high seas^11,33,34^. In contrast, sedimentary sequestration within coastal margins falls exclusively under national jurisdiction (Exclusive Economic Zones, EEZs). This sovereignty renders coastal blue carbon a tractable and accountable asset, offering a viable pathway for its formal integration into National Greenhouse Gas Inventories and, ultimately, voluntary carbon markets^11,33,34^.

Here, we present a global assessment that spatially resolves the Macroalgal Carbon Burial Index (MCBI) of coastal sediments. Leveraging the most comprehensive marine SAR database to date, we first established the depositional environment as a critical physical constraint governing sequestration. We then coupled this geological constraint with macroalgal NPP to delineate burial hotspots and characterize the biogeochemical mechanisms underlying MCBI. To provide a scalable predictive tool, we further validated coastline complexity as an informative geomorphological proxy for burial favorability. Beyond spatial extent, we explored the durability of this sink by weighting MCBI with centennial-scale organic-carbon transfer efficiencies, identifying coastlines where favorable burial conditions coincide with efficient long-term preservation. Upscaling to national jurisdictions, we identified where conservation, restoration, or macroalgal aquaculture would deliver the greatest carbon-burial benefit, providing a basis for differentiated management strategies. Finally, we evaluated the representation of these coastlines within existing marine protected areas (MPAs), quantifying protection gaps and analyzing conservation disparities across jurisdictions. Our study provides the spatial baseline needed to operationalize macroalgal carbon burial as a nature-based climate solution.

## Results and discussion

### Global assessment of Macroalgal Carbon Burial Index (MCBI) and its physical constraints

**Fig. 1:**
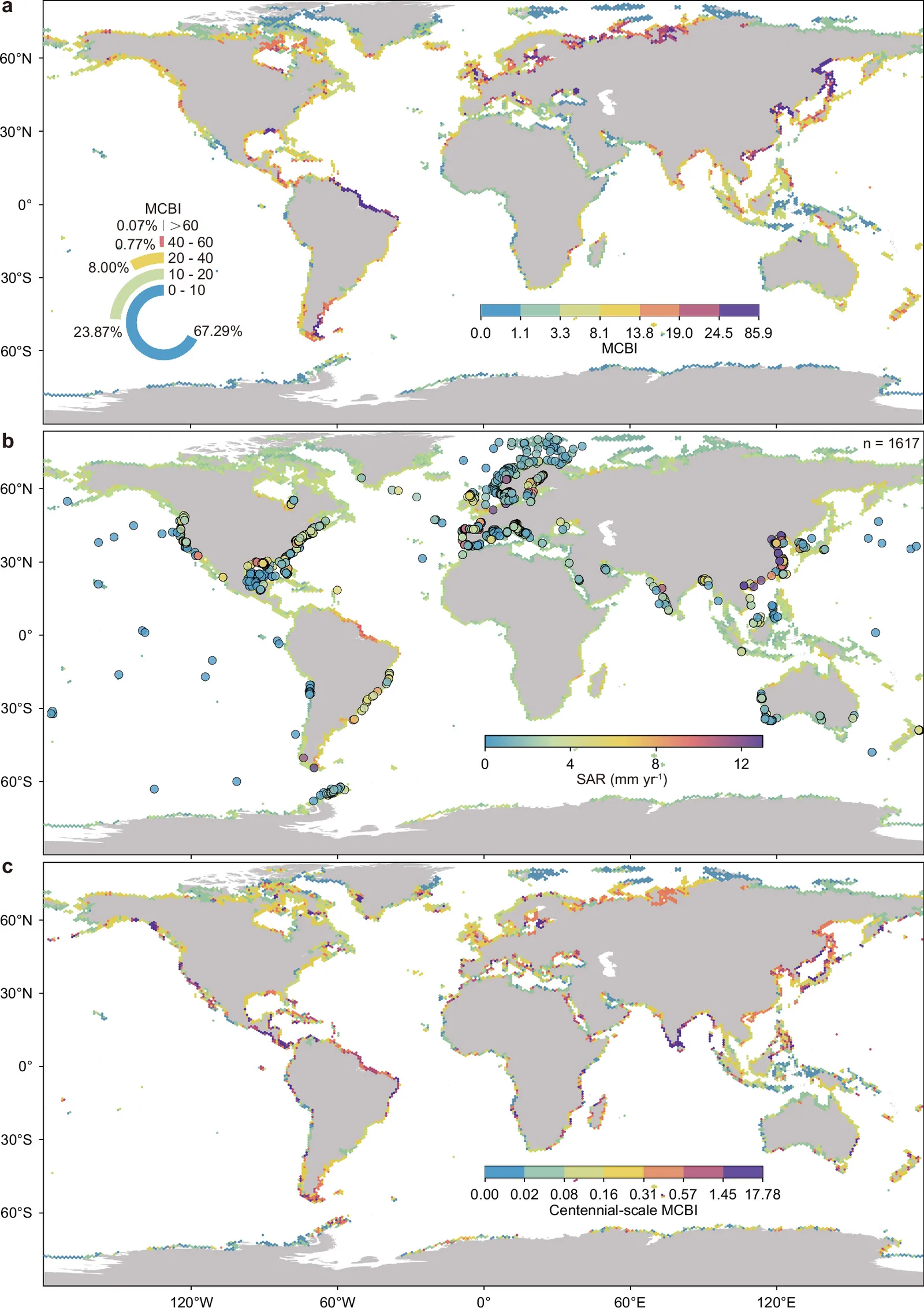
Global assessment of Macroalgal Carbon Burial Index (MCBI) and its physical foundation. **a** Spatial distribution of MCBI across global coastlines, and the inset shows the proportion of global coastlines within different MCBI ranges (0–10, 10–20, 20–40, 40–60, and >60). **b** Global predicted marine sediment accumulation rate (SAR, mm yr⁻¹) derived from a Gradient Boosting Regression (GBR) model, and dots indicate the locations of empirical SAR measurements (*n* = 1617) compiled to constrain the model. **c** Spatial distribution of the centennial-scale MCBI, obtained by weighting MCBI with the 100-year organic-carbon transfer efficiency of Bradley et al. ^40^. Base map data are from Natural Earth.

To characterize the global favorability of coastal environments to bury macroalgal carbon, we developed the Macroalgal Carbon Burial Index (MCBI; Fig. 1a), defined as the normalized product of macroalgal NPP and coastal SAR and expressed as a dimensionless index scaled 0–100 (Methods). Unlike previous estimates that relied on sparse observations^4,9^, our assessment is underpinned by a rigorous reconstruction of the global coastal sedimentary environment. We compiled a global database of marine SAR comprising 1617 records (Fig. 1b), integrating coastal observations together with records from estuaries, marginal seas, and the open ocean, spanning the range of depositional environments relevant to macroalgal carbon burial^35^. This expanded spatial coverage allows the model to move beyond localized extrapolation toward a global-scale assessment.

To predict the physical trapping efficiency (i.e., SAR) across global coastlines, we employed a data-driven Gradient Boosting Regression (GBR) model^36^, which achieved good explanatory power (R² = 0.64, Supplementary Fig. 1). This model integrated eleven environmental predictors across broad spatial gradients and distinct forcing mechanisms, encompassing climatic, hydrological, and physical oceanographic controls (detailed in Methods). Feature importance analysis identified total suspended matter and water depth as the dominant controls on the global spatial pattern of SAR (Supplementary Fig. 3), indicating that sediment accumulation is governed primarily by sediment supply and hydrodynamic setting. The modeled SAR averaged 3.3 ± 1.5 mm yr⁻¹ (mean ± SD; range: 0.1–13.2 mm yr⁻¹; Fig. 1b). We interpret modeled SAR as representing the depositional potential of the sedimentary environments within the same 5-arcminute spatial unit, specifically functioning to trap detritus exported from adjacent hard-substrate macroalgal habitats. This spatial coupling, where organic matter produced on hard-bottom habitats is sequestered in adjacent soft-sediment sinks, constitutes the most prevalent pathway for coastal macroalgal carbon burial^10,19,27,37^.

Our high-resolution modeling resolved the global coastal distribution of high-sedimentation regions, including the extensive coastlines of China, the western Korean Peninsula, the Pacific coast of Japan, and the Philippine coast, as well as localized high-SAR zones in the Gulf of Mexico (USA), the Amazon and Mississippi River estuaries, and the Joseph Bonaparte Gulf (Australia; Fig. 1b). These patterns are broadly consistent with independent global sediment-accumulation assessments^22^ and with established marine-geological understanding, confirming that favorable depositional environments are concentrated in river-dominated estuaries, semi-enclosed bays, and shallow continental shelves^26,38,39^. This consistency lends confidence to our SAR predictions, which we then combine with macroalgal productivity to characterize the relative favorability of coastal environments for macroalgal carbon burial.

Integrating this depositional constraint with biological productivity reveals the global landscape of MCBI (Fig. 1a). MCBI values range from 0 to 85.9 (theoretical maximum 100), with a global mean of 8.1, and the distribution is strongly right-skewed: 67.3% of coastlines record an MCBI below 10, while fewer than 1% exceed 40 (Fig. 1a, inset). High-value coastlines are consistently identified in several regions, including the northeastern coast of South America (notably the Amazon footprint), Bohai Bay and Korea Bay, southwestern Japan, the southern Patagonian fjords, and the Okhotsk-Sakhalin region (Fig. 1a and Supplementary Fig. 14), where high SAR coincides with high macroalgal NPP. We emphasize that MCBI is intended to characterize the conditions governing the burial favorability of macroalgal carbon at a given coastline, integrating NPP and SAR on a per-area basis. It therefore represents a relative index of macroalgal carbon burial favorability rather than an estimate of absolute carbon burial, and is best suited to global-scale prioritization of areas where coastal environments are physically conducive to macroalgal carbon sequestration. Because the underlying NPP estimates (Pessarrodona et al. ^35^) derive from ecological niche models of potential productivity, high-MCBI regions identify environments favorable for macroalgal carbon burial even where current biomass is locally depleted (e.g., by historical harvesting or overgrazing). Notably, however, because MCBI does not incorporate the actual extent of macroalgal habitat, it should not be interpreted as a measure of the total macroalgal carbon buried at a given location, nor as a substitute for local-scale carbon accounting. As a next step, integrating MCBI with observed or modeled macroalgal distributions (e.g., Duarte et al. ^1^) would advance toward the quantification of absolute burial fluxes, representing a promising direction for future work. Moreover, even where sizeable macroalgal stands are currently absent, MCBI can help identify locations where macroalgal aquaculture could yield the strongest carbon-burial benefits beneath the farm.

Beyond spatial extent, the long-term fate of buried carbon is governed by post-depositional preservation. To explore this dimension, we developed a centennial-scale index by weighting MCBI with the 100-year organic-carbon transfer efficiency of Bradley et al. ^40^, which estimates the fraction of deposited carbon that survives a century of degradation within the sediment (Supplementary Methods 4 and Fig. 1c). The resulting index preserves the principal spatial patterns of MCBI while additionally reflecting where buried carbon is more likely to be retained over centennial timescales (Supplementary Fig. 8). Coastlines that rank highly under both metrics, including the Okhotsk-Sakhalin region, the eastern Philippines, and the southern Patagonian fjords, represent settings where favorable burial conditions coincide with efficient long-term preservation, and thus constitute the most robust priorities for macroalgal carbon management (Fig. 1c). We regard this as an exploratory step toward longer-term carbon accounting rather than a definitive preservation estimate, as the underlying transfer-efficiency values derive from a global reaction-transport model that carries substantial uncertainty in nearshore and shelf settings. Nonetheless, coupling burial favorability with preservation efficiency illustrates a path toward resolving not only where macroalgal carbon is buried but also how durably it is stored.

As with all machine-learning-based global assessments, our predictions are subject to geographical bias arising from the uneven spatial distribution of available observations, which reflects historical differences in research effort rather than underlying environmental variability^41^. Overall, our SAR predictions are stable across most temperate and tropical coastlines (98.5% of coastlines with a coefficient of variation below 0.3 across 100 bootstrap iterations; Supplementary Methods 2, Supplementary Fig. 4), while larger uncertainties persist in under-sampled regions, such as the polar coastlines. To further evaluate where predictions are well constrained by data versus where they rely on extrapolation, we performed spatial block cross-validation and computed two complementary coverage metrics, the feature-space distance (mean Euclidean distance to the five nearest training observations in the standardized predictor space, excluding geographic coordinates) and the geographical distance to the nearest observations (Supplementary Methods 2)^41^. As expected for spatially autocorrelated data, the block cross-validation showed that absolute predictive skill decreased relative to random cross-validation, although the relative spatial ranking of predictions remained moderately preserved (Supplementary Methods 2). The two coverage metrics further reveal regions where apparent precision may be misleading. Areas with small feature-space distance but large geographical distance (e.g., the west coast of Africa) exemplify the decoupling between environmental representativeness and local data availability: predictions there may appear precise (low coefficient of variation) yet lack local validation, and should be interpreted with caution irrespective of their apparent uncertainty. Together, these analyses (Supplementary Figs. 4–6) delineate where the global predictions are robust and where they should be treated as extrapolation.

### Biogeochemical coupling underlying macroalgal carbon burial hotspots

**Fig. 2:**
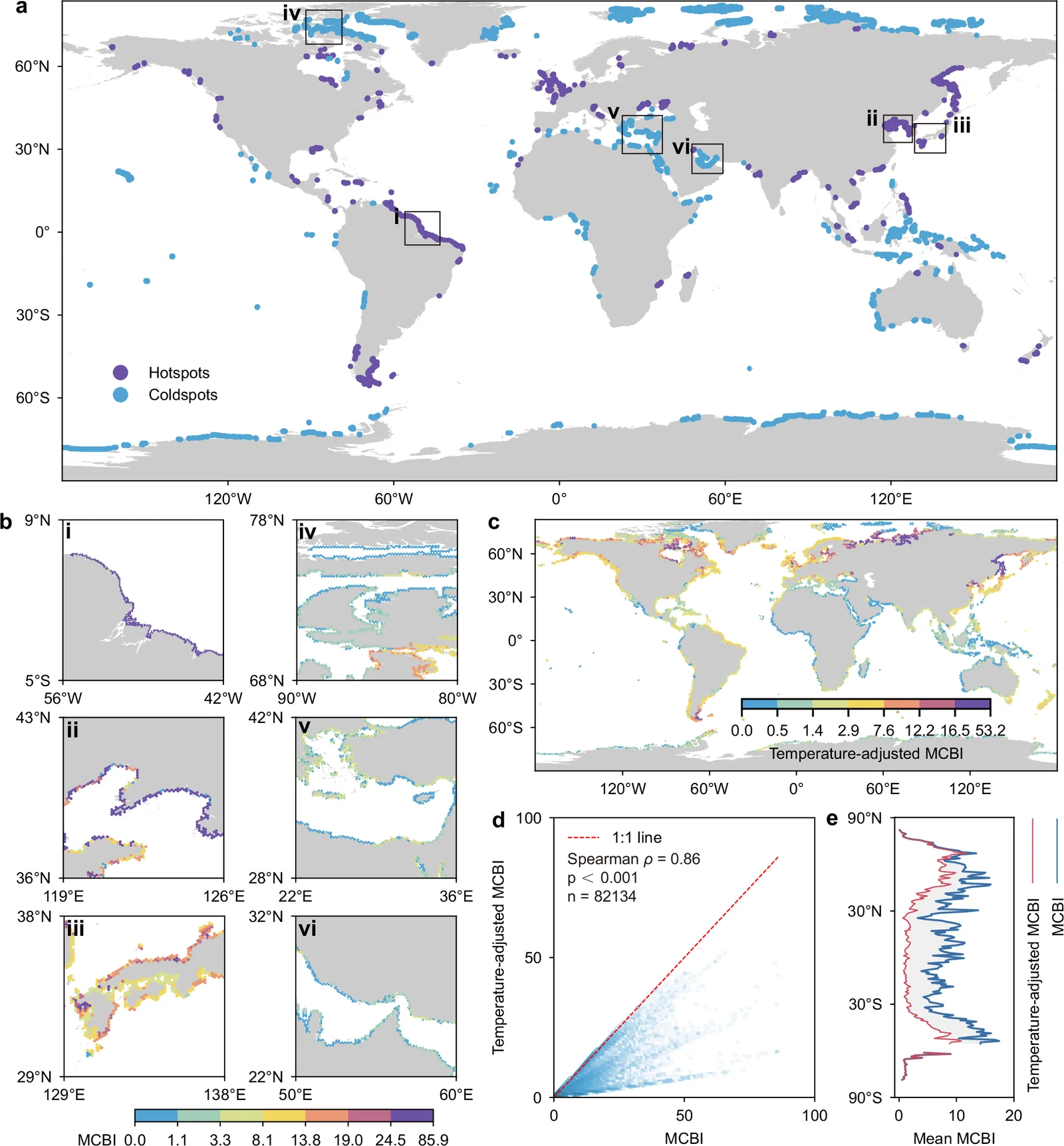
Biogeochemical coupling and thermal sensitivity of Macroalgal Carbon Burial Index (MCBI). **a** Hotspots and coldspots of MCBI on a global scale (identified as co-hotspots and co-coldspots of macroalgal net primary productivity (NPP) and sediment accumulation rate (SAR)); 95% confidence level. **b** Spatially explicit maps of MCBI in typical hotspot or coldspot areas: (**i**) the Amazon River estuary, (**ii**) the Bohai and Korea Bays, and (**iii**) the southwestern Japan as hotspots; (**iv**) the Canadian Arctic Archipelago, (**v**) the Eastern Mediterranean and (**vi**) the Persian Gulf as coldspots. **c** Global distribution of temperature-adjusted MCBI, incorporating a temperature-dependent remineralization factor (Q_10_ = 2.0). **d** Relationship between the original and temperature-adjusted MCBI, assessed by Spearman’s rank correlation (*ρ* = 0.86, *p* < 0.001, *n* = 82,134). The red dashed line denotes the 1:1 relationship. **e** Latitudinal distribution of the original and temperature-adjusted MCBI (zonal means). Base map data are from Natural Earth.

The spatial distribution of MCBI is governed by the nonlinear interaction between organic carbon supply (macroalgal NPP) and geological burial capacity (SAR). To disentangle the drivers of coastal carbon sequestration, we employed the Getis-Ord Gi* statistic, a spatial clustering method, to identify significant hotspots and coldspots (95% confidence level)^42^, revealing distinct regimes of biotic-abiotic coupling (Methods; Fig. 2a). Our analysis reveals that significant carbon sinks are not randomly distributed but emerge as co-hotspots where high macroalgal NPP coincides with favorable sedimentation. Notable examples include the aforementioned Amazon estuary, the Bohai and Korea Bays, and parts of the Japanese coast (Fig. 2a, b). In these optimal sequestration settings, the convergence of intense sedimentation and robust macroalgal productivity driven by favorable climatic and hydrological conditions maximizes the trapping efficiency of organic matter^35^.

Conversely, at the highest latitudes (poleward of ~70° N and ~60° S), coasts generally manifest as coldspots (Fig. 2a, b), primarily driven by severe biological constraints (e.g., seasonal light limitation, persistently low temperatures, and extensive sea-ice cover) that restrict the extent and annual productivity of macroalgae^35,43,44^, combined with open glacial shelves where SAR remains below the global average^45,46^. Localized exceptions nonetheless occur even at these latitudes: along the Kara Sea coast, for example, exceptionally high macroalgal productivity coincides with favorable, river-driven sedimentation giving rise to burial hotspots^35,47^ (Fig. 1a). It is also critical to distinguish these vast open polar shelves from localized topographic traps such as fjords^48^. Fjords function as highly efficient macroalgal particulate sinks through a dual mechanism: they restrict the offshore export of in situ macroalgal biomass while simultaneously receiving large allochthonous detrital subsidies funneled from adjacent coastal habitats^16,32^. This dual input explains the strong macroalgal carbon and eDNA signatures frequently observed in polar fjord sediments^49,50^, and accordingly, our model does not uniformly classify fjord-dense coastlines (e.g., Norway and coastal Greenland; Fig. 2a) as coldspots. Nevertheless, we acknowledge that at 5-arcminute resolution, and given empirical data scarcity, our framework cannot fully separate micro-scale fjords from surrounding open shelves, nor does our local-coupling approach explicitly route cross-pixel allochthonous subsidies; higher-resolution hydrodynamic models will be required to resolve these sub-grid dynamics. In contrast to these spatially heterogeneous polar systems, the Eastern Mediterranean and the Persian Gulf manifest as consistent coldspots, driven by oligotrophic conditions and limited terrigenous input across narrow continental shelves (Fig. 2b)^22,51,52^.

At the global scale, MCBI exhibits a latitudinal pattern broadly consistent with that of macroalgal NPP, with high values concentrated at mid-to-high latitudes (approximately 30°-70° N and 30°-60° S), where high macroalgal productivity and favorable sedimentation coincide, reflecting a spatial coupling of NPP and SAR^25,35^ (Supplementary Fig. 15). In the tropics (approximately 30° S-30° N), brown-algal productivity declines sharply or disappears altogether (e.g., along the west coast of Africa), leaving red algal turfs as the dominant macroalgal contributor (Supplementary Figs. 16, 18), typically an order of magnitude lower in productivity than brown-algal forests^35^. Consequently, MCBI is generally low across most tropical coastlines. Notable exceptions arise where exceptional geological conditions compensate: along the coasts of the Philippines, for example, the combination of rapidly turning-over algal turfs^53^ and high SAR driven by small mountainous rivers sustains locally high burial favorability^38,54^ (Fig. 1a, Supplementary Figs. 18, 19).

Our current framework focuses on quantifying the geophysical boundary conditions favorable for macroalgal carbon burial, yet the realized long-term preservation of buried macroalgal carbon is further modulated by biological and environmental loss pathways prior to ultimate sequestration. Temperature-driven microbial decomposition is regarded as a key control on macroalgal organic carbon preservation: recent large-scale field evidence shows that macroalgal detritus decomposition is strongly temperature-dependent, and microbial remineralization fundamentally follows Arrhenius-type kinetics^55^. To explore the spatial heterogeneity this introduces, without over-parameterizing our dimensionless index, we conducted a theoretical sensitivity analysis by applying a standard biological temperature coefficient (Q_10_ = 2.0) to formulate a relative thermal retention factor (*f*)^18,56^, and incorporated it into the MCBI (Supplementary Methods 5, Fig. 2c). The temperature adjusted MCBI remained strongly correlated with the original metric (Spearman *ρ* = 0.86, *p* < 0.001 and Fig. 2d), indicating that the relative ranking of burial hotspots is robust to this adjustment. Spatially, the adjustment primarily reduced MCBI in the equatorial zone (−30° to 30°), while high-latitude hotspots remained largely unaffected (Fig. 2e), reflecting stronger temperature-driven remineralization in warm tropical waters. Importantly, however, the high sediment accumulation rates characteristic of many mid-to-low-latitude coastlines may counterbalance this thermodynamic penalty: rapid mineral input facilitates the swift burial of detritus below the active oxic zone, shielding organic matter from rapid microbial decay^26^.

Beyond thermal controls, the long-term preservation of macroalgal carbon is also affected by biological consumption by benthic fauna (e.g., grazing by sea urchins^28^) and by physical processes that decouple SAR from net carbon preservation. Although SAR broadly governs the transfer efficiency of organic carbon into deeper sediment layers^40^, and net preservation generally scales positively with SAR, this coupling can break down in hyper-dynamic settings^40^. In high-energy mobile muds (e.g., the Amazon-Guianas mudbelt^57^) and strongly resuspended large-river estuaries^51^, where deep sediment mixing repeatedly re-exposes buried material, intense physical reworking subjects organic matter to prolonged oxic degradation, so that high gross SAR does not translate into long-term preservation^40,51,57^. Future macroalgal blue-carbon models should prioritize integrating spatially resolved benthic food-web dynamics, diagenetic processes, and high-resolution coastal hydrodynamics to move from mapping burial favorability toward quantifying precise carbon fluxes.

### Coastline complexity contributing to macroalgal carbon burial

**Fig. 3:**
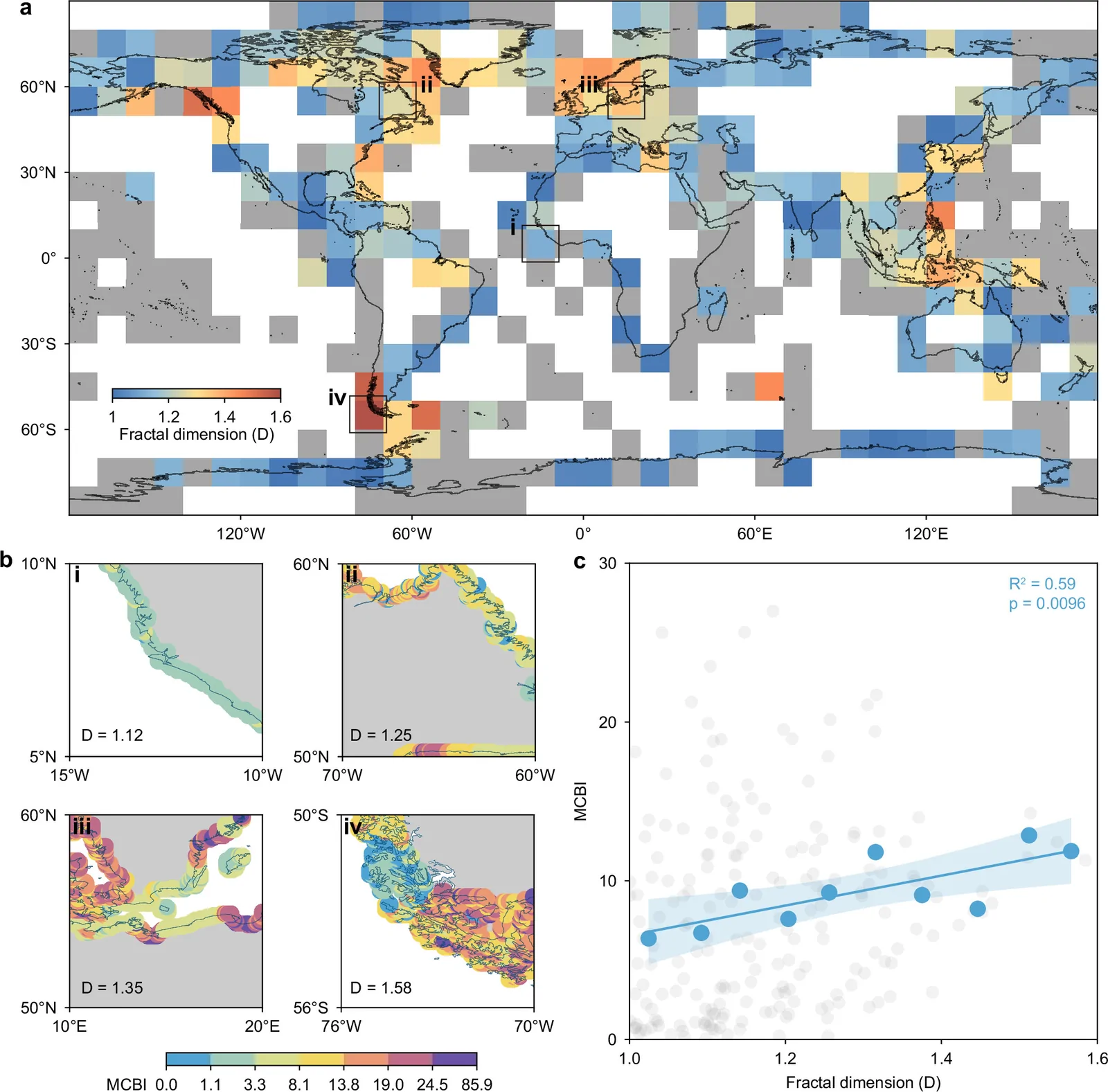
Coastline complexity as a predictor of Macroalgal Carbon Burial Index (MCBI). **a** Global distribution of coastline fractal dimension (D), calculated within 10° × 10° grid cells. Higher D values indicate greater coastline complexity. Gray grid cells indicate areas without valid fractal-dimension estimates. **b** Representative examples illustrating different levels of coastline complexity: (**i**) simple, (**ii**) moderately complex, (**iii**) complex, and (**iv**) ultra-complex coastlines. **c** Relationship between coastline fractal dimension (D) and MCBI. Blue dots represent mean MCBI values within 0.06-wide fractal-dimension bins, and the solid line represents the linear regression fitted to these binned values (R² = 0.59, *p* = 0.0096, *n* = 10). Gray dots represent the underlying unbinned data (n = 202). Base map data are from Natural Earth.

The global distribution of MCBI shows a geometric dependency, with high-favorability sinks preferentially located along structurally complex coastlines (Fig. 3a, b). To quantify this geomorphological control, we evaluated the relationship between MCBI and coastline complexity, measured by the fractal dimension (D)^58^. Our analysis demonstrates a robust positive correlation globally (R² = 0.59, *p* = 0.0096, *n* = 10; Fig. 3c), identifying coastline complexity as an informative predictor of macroalgal carbon burial. This implies that the geometric intricacy of the shoreline itself is an important determinant of a region’s favorability for retaining macroalgal carbon.

Mechanistically, coastline complexity enhances macroalgal carbon burial by exerting controls on nearshore hydrodynamics and sediment retention, while simultaneously expanding macroalgal habitat availability. Geomorphologically complex coastlines, such as fjords, semi-enclosed bays, or coastlines fringed by islands and spits, can act as hydrodynamic barriers that dissipate wave energy and reduce current velocities. These conditions increase water residence time, thereby enhancing particle-trapping efficiency and increasing the likelihood that suspended particulate organic matter, including macroalgal-derived POC, settles locally rather than being advected offshore^4,59,60^. In contrast to rooted blue carbon ecosystems such as mangroves and seagrasses, where in situ burial is promoted through sediment stabilization^61^, the unrooted nature of macroalgae renders their long-term sequestration fate particularly sensitive to external geomorphic and hydrodynamic constraints^10^. Within geomorphologically complex, low-energy systems, such as fjord networks exemplified by Greenland, dislodged macroalgae behave less as transient export vectors and more as retained particles, facilitating effective local burial^62^. Beyond physical retention, coastline complexity also exerts an indirect control on carbon burial by increasing the extent and heterogeneity of suitable macroalgal habitat. Complex shorelines increase shoreline length and microhabitat diversity, supporting higher standing stocks of macroalgal biomass per unit linear coastline^63^. Consistent with geometric principles linking structural complexity to total abundance^63^, coastlines with high fractal dimensions therefore combine enhanced organic matter production with favorable physical conditions for sedimentary retention. Together, these coupled biological and physical effects enable geomorphologically complex coastlines to function as efficient and self-reinforcing sedimentary carbon sinks.

Furthermore, coastline complexity serves as a practical, low-cost proxy for identifying high-value carbon sinks, particularly in data-poor regions where direct measurements of SAR or NPP are unavailable. By integrating this geomorphic indicator into spatial planning, conservationists can prioritize regions possessing high natural macroalgal carbon burial favorability. This offers a geophysically grounded framework for designing MPAs and siting restoration projects, ensuring that interventions are targeted where the landscape itself maximizes the return on blue carbon investment.

### National disparities and differentiated management strategies

**Fig. 4:**
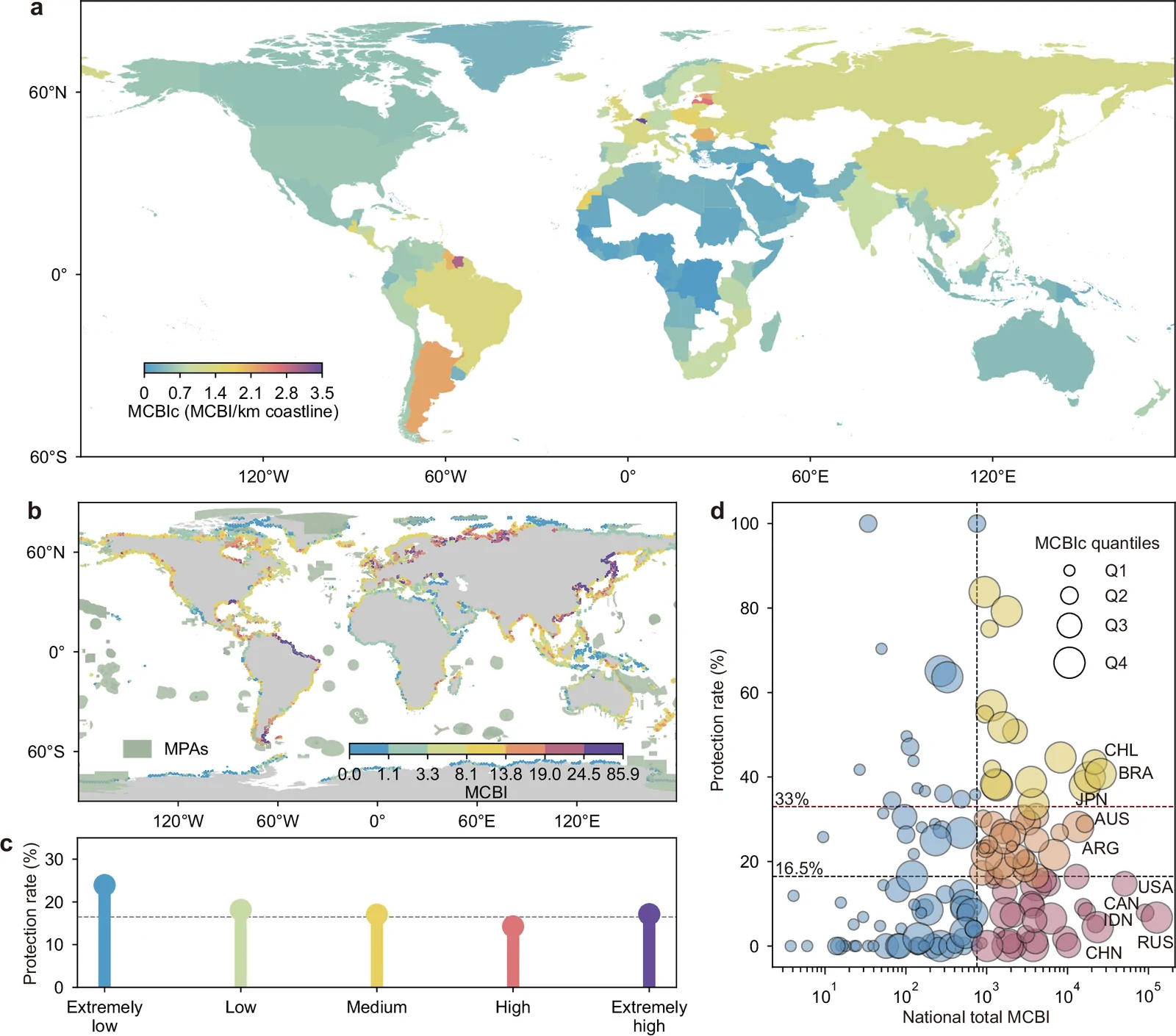
National disparities in macroalgal carbon burial favorability and functional mismatches in marine protection. **a** Average Macroalgal Carbon Burial Index (MCBI) per kilometer of coastline (MCBIc) for coastal nations and regions. **b** Overlap between Marine Protected Areas (MPAs) and MCBI. **c** MPA protection rate across MCBI quintiles (Extremely low, below the 20th percentile; Low, 20–40th; Medium, 40–60th; High, 60–80th; Extremely high, above the 80th percentile). **d** Relationship between national total MCBI and MPA protection rate. Bubble sizes are proportional to MCBIc. Q1–Q4 denote the four quartiles of national MCBIc, from the lowest (Q1) to the highest (Q4). Country codes denote CHL, Chile; BRA, Brazil; JPN, Japan; AUS, Australia; ARG, Argentina; USA, United States; CAN, Canada; IDN, Indonesia; RUS, Russia; and CHN, China. The red dashed line marks a 33% protection-rate reference level; the two black dashed lines denote the median national total MCBI and the global average protection rate, respectively. Base map data are from Natural Earth. MPA boundaries are from the World Database on Protected Areas (WDPA; IUCN and UNEP-WCMC, 2024; www.protectedplanet.net).

The burial favorability quantified by MCBI is distributed with pronounced geographical asymmetry. At the continental scale, summed MCBI is concentrated in Asia and North America, which together account for approximately 31.5% and 28.0% of the global MCBI total, respectively, whereas Europe, South America, and Africa contribute comparatively less (19.8, 11.6, 3.5%). This concentration persists at the national scale: the top five nations (Russia, 19.0%; Canada, 13.6%; the United States, 7.6%; Brazil, 3.8%; and China, 3.5%) together account for nearly half of the global MCBI total. We emphasize that these shares reflect the geographical distribution of coastlines favorable for burial, not estimates of the carbon actually buried, which additionally depends on realized macroalgal extent and detrital supply. To separate this pattern from coastline length, we normalized MCBI by coastline length (MCBI per kilometer, MCBIc). This per-coastline metric reveals even greater heterogeneity, spanning a > 281-fold range across nations (0.012 to 3.5; Fig. 4a). The highest-ranking jurisdictions by MCBIc, including Belgium, Suriname, Latvia, Estonia, and Argentina, have coastlines with high per-kilometer burial favorability, where targeted interventions such as habitat conservation or macroalgal aquaculture could yield substantial carbon-burial benefit relative to the length of coastline involved.

Our framework can also inform active interventions such as macroalgal aquaculture. MCBI is well suited to identifying candidate coastlines at broad scales, as it captures both the environmental suitability for macroalgal growth (a prerequisite for productive farming) and the depositional conditions required for the resulting carbon to be buried. Within environments already suitable for macroalgae, SAR becomes the key factor distinguishing sites where farmed biomass and its detritus are most efficiently trapped and buried, since aquaculture directly supplements biomass supply^4^. Even if the primary macroalgal biomass is ultimately harvested, the POC detached and the DOC released during the growth phase represent a substantial and persistent carbon flux to the surrounding environment^4^. Identifying coastlines that combine high macroalgal suitability with high SAR thus provides a grounded basis for siting macroalgal aquaculture so that farm-derived carbon is efficiently buried beneath and adjacent to the farm. Notably, a substantial share of the coastlines identified here as combining high MCBI with high sedimentation lies within Small Island Developing States (e.g., Suriname, Guyana, Jamaica and Haiti) and low- to lower-middle-income nations (e.g., the Philippines, Tanzania and Mozambique; Fig. 1a, b). For these countries, this represents a domestic natural asset: macroalgal aquaculture is already recognized as an accessible, low-capital livelihood^5^, and coastlines where the underlying sediments efficiently retain the resulting carbon offer an opportunity to couple that livelihood with a verifiable, nationally accountable climate benefit. Asia currently offers the clearest working example of this coupling, where major producers (e.g., China, Indonesia, Japan and South Korea) farm in coastal settings whose high sedimentation rates facilitate the incidental burial of farm-derived biomass^4,5^. Translating this potential into a verifiable climate solution, however, requires standardized frameworks for Monitoring, Reporting and Verification (MRV)^64^. The dispersive nature of macroalgal carbon makes it inherently harder to attribute to a given farm than the in situ burial of rooted blue carbon habitats: quantifying the net sequestration efficiency of exported detrital POC and tracking DOC export and its conversion to refractory DOC remain open challenges. Closing this gap will require pairing fine-scale field measurements with high-resolution local hydrodynamic models to resolve the ultimate fate of farm-derived carbon.

### Aligning marine protection with macroalgal carbon burial hotspots

We examined the coherence between marine protection and coastlines favorable for macroalgal carbon burial. The results showed that protection coverage is disproportionately skewed towards regions of minimal burial favorability (Fig. 4b). Specifically, the ‘Extremely low’ MCBI quintile has the highest protection rate (23.9%), whereas regions categorized as ‘High’ and ‘Extremely high’ receive lower protection (14.3% and 17.1%, respectively), aligning closely with the global weighted average of ~16.5% (Fig. 4c). This indicates that current MPA networks are functionally carbon-blind, at least for macroalgal carbon burial. While vast stretches of coastline with limited burial favorability are safeguarded to prioritize other conservation objectives, the primary macroalgal carbon burial hotspots characterized by the highest MCBI remain no better protected than the global baseline, leaving key climate assets vulnerable to anthropogenic disturbance.

To identify the jurisdictional drivers of this gap, we mapped national total MCBI against MCBI-weighted protection rate (Fig. 4d). We adopt a 33% protection rate level as a diagnostic reference, approximately twice the global weighted average (16.5%; Fig. 4d). Among nations whose total MCBI exceeds the global median, we distinguish three groups. A cluster of 30 nations, including Russia, Canada, the United States, China, and Indonesia, falls into a critical-action zone, with protection rates at or below the global weighted average (16.5%) and thus far short of the 33% reference level, despite these nations collectively accounting for the majority of the global MCBI total. An intermediate cohort of 27 nations, typified by Australia and Argentina, exceeds the global average but has yet to reach the 33% target. Only 17 jurisdictions (e.g., Japan, Chile, and Brazil) sit above the reference level, showing that substantial burial favorability and effective conservation coverage can coexist. For the protection-deficit jurisdictions, our high-resolution MCBI assessment provides a spatially explicit basis for directing future conservation expansion toward the coastlines where conditions are most favorable.

While the primary objective of MPAs has been to conserve biodiversity and critical habitats^65,66^, the integration of carbon sequestration as a conservation criterion remains inconsistent across marine ecosystems. Macroalgal habitats receive disproportionately less targeted protection compared to often-degraded coastal wetlands, such as salt marshes, mangroves, and seagrasses^67–69^, despite evidence of their comparable or superior carbon storage capacity^4,9^. As the international community mobilizes to meet the ‘30×30’ target (protecting 30% of the ocean by 2030)^70^, a strategic realignment is essential. Under a scenario in which 30% of potential macroalgal habitat is protected, directing new protection toward the most favorable unprotected coastlines would raise the mean MCBI of the protected network to 13.5, compared with ~7.5 under a random expansion of identical extent, and 4.6 were the least favorable coastlines selected instead (Supplementary Methods 8, Supplementary Table 5). Strategic siting therefore offers a ~ 1.8-fold gain relative to random expansion, and up to ~2.9-fold relative to least-favorable siting. Such a targeted realignment must proactively navigate socioeconomic trade-offs, particularly in the densely populated coastal zones of the critical action nations. However, by utilizing MCBI as a precision siting tool, policymakers can maximize climate mitigation returns per unit of protected area, transforming macroalgal conservation from a passive co-benefit into a deliberate nature-based climate solution.

## Methods

### Sediment accumulation rate (SAR) data compilation

We compiled a comprehensive global database of marine SAR through a multi-stage systematic synthesis. The historical foundation of the database (prior to 2020) was underpinned by records from Restreppo et al.^22^. and our previously published research on coastal blue carbon ecosystems^71^. To ensure the database reflected the most current observations, we conducted a systematic literature review for the 2020-2025 period. We queried the Web of Science using a targeted Boolean string: (‘sedimentation rate’ OR ‘sediment accumulation’ OR ‘sediment deposition’ OR ‘SAR’ OR ‘carbon accumulation’ OR ‘CAR’ OR ‘carbon deposition’) AND (‘sea’ OR ‘coastal’ OR ‘coast’ OR ‘ocean’ OR ‘oceanic’ OR ‘marine’ OR ‘shore’ OR ‘estuary’ OR ‘tidal’ OR ‘pelagic’). From an initial pool of 9,986 records, we identified 46 key publications that yielded 906 recently published SAR entries. Following rigorous quality control and the removal of duplicates, the final consolidated database comprised 1,617 unique entries. This extensive dataset captures a highly diverse range of depositional environments globally, including a substantial volume of records specifically from coastal habitats (51.6%), supplemented by observations from estuaries, marginal seas, and the open ocean. Conventional radioisotope methods (specifically ^210^Pb, ^137^Cs, and ^14^C) constitute a significant proportion (92.0%) of the SAR measurement techniques in our database (see Data availability). Furthermore, some SAR data for which specific methods were not provided in the source literature were primarily derived as byproducts of long-term organic carbon burial estimations. Consequently, these SAR records predominantly characterize long-term average depositional states spanning decadal to centennial timescales.

### Prediction of the global SAR

To spatially resolve SAR across global macroalgal habitats, we implemented a Gradient Boosting Regression (GBR) framework^36^, a robust machine learning approach capable of capturing complex, non-linear environmental interactions. The modeling procedure consisted of four sequential stages: (i) Feature compilation and association, in which each compiled SAR record was linked to a suite of spatially explicit environmental predictors; (ii) Target transformation, in which the strongly right-skewed SAR observations were modeled in log space as ln(1 + SAR), stabilizing the variance and preventing a small number of high-magnitude records from dominating the squared-error loss^22^; (iii) Model training, wherein the GBR algorithm was fitted to the transformed dataset (*n* = 1617); (iv) Parameter tuning and evaluation, aimed at optimizing hyperparameters and rigorously assessing predictive performance (Supplementary Methods 1).

We compiled 11 potential environmental drivers to parameterize the global depositional environment. Geographic coordinates (latitude and longitude) were incorporated to capture broad-scale spatial gradients not resolved by local variables. The remaining predictors were categorized into three forcing mechanisms: (i) Climate change parameters, specifically relative sea-level rise rates and tropical cyclone effects^51,71^; (ii) Sediment supply factor, including total suspended matter^22^; and (iii) Physical oceanographic controls, encompassing water depth, mean tidal range, and current velocities/directions (surface and seafloor)^51^. Collinearity among predictors was screened using the variance inflation factor (VIF); all eleven predictors showed no substantial collinearity (VIF < 2). Detailed sources for all variables are provided in Supplementary Table 2.

Model performance was evaluated using the coefficient of determination (R²) and mean squared error (MSE) on a held-out test set (10%), with five-fold cross-validation on the training partition employed to maximize generalization ability. To ensure methodological robustness, we benchmarked the GBR against five alternative algorithms: Support Vector Regression (SVR), K-Nearest Neighbors (KNN), Multilayer Perceptron (MLP), Random Forest (RF), and Extreme Gradient Boosting (XGB), all trained and tuned under identical conditions. The three tree-based ensembles clearly outperformed the distance-based models, with GBR performing marginally best; GBR was therefore selected as the final model (Supplementary Table 3). After subsequent hyperparameter optimization, the tuned GBR achieved R² = 0.64 (log space; Supplementary Fig. 1). To further evaluate model stability under data perturbation, we conducted a Monte Carlo cross-validation with 100 iterations (Supplementary Methods 1, Supplementary Figs. 2, 3).

Given the scarcity of empirical SAR measurements specifically within macroalgal beds, our model was trained on a broad spectrum of depositional environments (e.g., salt marshes, mangroves, estuaries, and shelves). By parameterizing SAR based on fundamental physical drivers (such as hydrodynamics and sediment supply) rather than specific habitat labels, we assume that the physical controls on sedimentation are broadly transferable across these settings. This assumption necessarily has limits: as with all gglobally trainedmodels, the environmental products used here carry greater uncertainty in nearshore than in open-ocean environments, which may introduce local biases in coastal SAR predictions.

### Calculation of the Macroalgal Carbon Burial Index (MCBI) and geostatistics

We computed the MCBI at a spatial resolution of 5 arc-minutes. The metric was constructed by coupling the biological carbon source (NPP) with the geological sink potential (SAR). NPP for each grid cell was derived from the niche-model-based potential NPP of three macroalgal functional groups (red algal turfs, intertidal brown algae, and subtidal brown algae) reported by Pessarrodona et al.^35^. To avoid double-counting productivity within the brown-algal canopy, we took the maximum of the intertidal and subtidal brown-algal values rather than their sum, and combined this with the red algal turf value. To mitigate the influence of extreme model artifacts and ensure conservative estimates, NPP values were capped at the 95^th^ percentile, with all values exceeding this threshold reassigned to the 95th-percentile value^10^. The resulting quantity represents the maximum potential macroalgal productivity attainable under environmentally suitable conditions, rather than an estimate of realized, co-occurring biomass; it therefore does not account for shading interactions between canopy and understory groups where these physically co-occur^72^. This potential-based definition is consistent with the purpose of MCBI as a relative index of burial favorability. Separate analyses for individual functional groups are provided in Supplementary Results 3. Predicted SAR was likewise capped to limit the influence of extreme values: in log space, predictions exceeding four standard deviations above the mean (z > 4) were reassigned to that threshold, corresponding to ~13.2 mm yr⁻¹ on the original scale and affecting 0.09% of predicted cells. This step is important because MCBI is constructed through Min-Max normalization, so that the upper bound of the SAR layer sets the scale for the entire index; capping prevents a small number of extreme predictions, most likely to arise in environmentally or geographically extrapolated regions, from determining the normalization of all coastal cells. Values were capped rather than removed, preserving complete global coastal coverage, and the uncapped SAR predictions are provided in the deposited dataset (Data availability).

To integrate these variables with distinct dimensionalities, we first normalized the macroalgal NPP and modeled SAR using Min-Max scaling (Eqs. 1 and 2). This standardization rescaled all values to a dimensionless range of [0, 1], thereby ensuring that both biological supply (NPP) and geological burial (SAR), as key co-limiting factors, contribute equally to the MCBI without bias from their original magnitude scales or units.

1 $${{{{\rm{NPP}}}}.{{{\rm{nom}}}}}_{i}\,=\,\frac{{{{{\rm{NPP}}}}}_{i}\,-\,{{{\rm{NPP}}}}.\min }{{{{\rm{NPP}}}}.\max -{{{\rm{NPP}}}}.\min }$$

2 $${{{{\rm{SAR}}}}.{{{\rm{nom}}}}}_{i}\,=\,\frac{{{{{\rm{SAR}}}}}_{i}\,-{{{\rm{SAR}}}}.\min }{{{{\rm{SAR}}}}.\max -{{{\rm{SAR}}}}.\min }$$

MCBI was then derived as the coupled product of these normalized factors, scaled by 100 to facilitate interpretability (Eq. 3):

3 $${{{{\rm{MCBI}}}}}_{i}\,=\,{{{{\rm{NPP}}}}.{{{\rm{nom}}}}}_{i}\,\times \,{{{{\rm{SAR}}}}.{{{\rm{nom}}}}}_{i}\,\times 100$$

Because MCBI is Min-Max normalized, any globally uniform rescaling of NPP, such as applying a constant detrital-to-NPP conversion factor (0.71) to represent the fraction of production available for transport^10^, leaves the normalized values and the relative spatial ranking unchanged, and is therefore not applied explicitly.

It is important to clarify the biogeochemical nature of the carbon pools represented in our framework. The modeled macroalgal NPP estimates utilized here represent total primary production, which inherently encompasses both particulate organic carbon (POC; fragmented macroalgal particles and larger, stochastically dislodged macroalgal biomass) and dissolved organic carbon (DOC) exuded during growth. However, our MCBI metric is fundamentally constrained by the physical trapping mechanism of SAR, which primarily acts upon the settling particulate fraction. In contrast to DOC, whose long-term sequestration primarily relies on export below the mixed layer or transformation into refractory DOC (RDOC), the MCBI effectively represents the favorability of coastal environments to trap and sequester the particulate fraction (detritus and dislodged biomass) of the local macroalgal production.

To enable equitable cross-national comparisons independent of geographic scale, we calculated the MCBI per kilometer of coastline (MCBIc) for each jurisdiction (Supplementary Table 7). This metric neutralizes the bias arising from vast disparities in coastline lengths (e.g., small island states vs. continental nations).

To assess the alignment between marine protection and macroalgal carbon burial favorability, we performed a spatial overlap analysis between the global MCBI raster and Marine Protected Area (MPA) polygons obtained from the World Database on Protected Areas (WDPA; IUCN and UNEP-WCMC, 2024; www.protectedplanet.net). First, the global MCBI dataset was stratified into five quintile-based categories (ranging from ‘Extremely low’ to ‘Extremely high’) to establish functional baselines. We then quantified the protection rate using a value-weighted approach rather than a simple area-based count. Specifically, we calculated the cumulative sum of MCBI values falling within MPAs' boundaries for each quintile category and divided this by the total MCBI sum of the respective category. This procedure provides a functional assessment of how well the carbon sequestration favorability, rather than just the physical area, is represented within the existing conservation network (Supplementary Table 4). All spatial analyses were conducted using ArcGIS Pro 3.0 and Python environments.

### Assessment of biotic-abiotic coupling (co-hotspots analysis)

To spatially resolve the coupling between biological supply and geological burial, we identified joint hotspots and coldspots of macroalgal NPP and SAR. We employed the Getis-Ord Gi* statistic to delineate statistically significant spatial clusters of high values (hotspots) and low values (coldspots) for both NPP and SAR^42^. To ensure rigorous spatial calculations, all raster layers were reprojected from WGS84 to a Cylindrical Equal-Area projection (EPSG:6933) to minimize areal distortion. We constructed the spatial weight matrix using a k-nearest neighbors approach (k = 8), which ensures robust connectivity even for isolated coastal features. The analysis was conducted at a 95% confidence level (*p* < 0.05). Finally, we defined co-hotspots as the spatial intersection of significant NPP hotspots and SAR hotspots, and co-coldspots where both variables formed significant NPP coldspots and SAR coldspots. This intersection mask delineates regions with theoretically optimal (or minimal) macroalgal carbon burial favorability.

### Assessment of coastline complexity

To quantify the geometric control on carbon burial, we calculated the fractal dimension (D) of coastlines using the standard box-counting method^58,73^. The fractal dimension is a well-established metric in geophysical and ecological research for characterizing shoreline irregularity^74,75^. The global coastline (sourced from Natural Earth) was tessellated into a 10°×10° grid system (Fig. 3a). For each grid cell, we computed D to characterize shoreline irregularity, utilizing a reproducible automated workflow (Supplementary Methods 6). To ensure statistical robustness, grid cells with negligible coastline segments or resolution artifacts yielding theoretically invalid dimensions (D < 1) were excluded. The final dataset comprised 202 valid grid cells (Supplementary Table 6).

To investigate the relationship between complexity and carbon sequestration, we paired the computed D with the mean MCBI value for each grid. To mitigate spatial noise and enhance trend interpretability, we applied an equal-interval binning strategy based on the fractal dimension range and performed regression analysis on the binned averages (R²; Supplementary Methods 7). We further evaluated the robustness of this relationship using sensitivity analyses across alternative binning schemes (Supplementary Methods 7).

## Supplementary information

Supplementary Information

Transparent Peer Review file

## Source data

Source Data

## Data Availability

The comprehensive global database of marine sediment accumulation rates (SAR) compiled in this study, the predicted global SAR raster, the generated Macroalgal Carbon Burial Index (MCBI) datasets (encompassing distributions for total macroalgae, brown algal forests, and red algal turfs), and the centennial-scale MCBI have been deposited in Figshare (https://doi.org/10.6084/m9.figshare.31042840). Data regarding coastline fractal dimensions and MPA protection rates for each jurisdiction are provided in the Supplementary Information accompanying this article. Source data for macroalgal NPP were derived from previously published work (ref. ^35^.), as cited in the Methods. Source data are provided with this paper.

## Code availability

The custom Python scripts developed for the Gradient Boosting Regression (GBR) model and coastline fractal dimension calculations have been deposited in Figshare (https://doi.org/10.6084/m9.figshare.31042840).
