# Supplementary material for: The global distribution of macroalgal carbon burial in coastal sediments
Source: Nat Commun. 2026 Aug 31;17:10372. doi: 10.1038/s41467-026-77215-7 (PMC13627689; doi:10.1038/s41467-026-77215-7)
Supplement: Supplementary file 1 [file 41467_2026_77215_MOESM1_ESM.pdf]

The Global Distribution of Macroalgal Carbon Burial in Coastal Sediments
Supplementary information

This supplementary document contains the following information:
Supplementary Methods 1 - 9
Supplementary Results 1 - 6
Supplementary Fig. 1 - 19
Supplementary Table 1 - 8

Supplementary Methods 1: Sediment Accumulation Rate (SAR) database and machine learning framework

1. SAR dataset and metadata

The compiled global marine sediment accumulation rate (SAR) database is publicly accessible via the Figshare repository (see Data Availability statement in the main text). To ensure transparency, reproducibility, and to facilitate cross-regional comparisons, we standardized the metadata for all 1,617 records. The structure and detailed description of the metadata fields are outlined in Supplementary Table 1. Missing or unclear metadata were retained as unspecified rather than inferred, except for sampling year, for which the publication year was used when the exact sampling date was unavailable. SAR values were standardized to consistent units (mm yr^{-1}) before database integration. Records were subsequently subjected to quality control and duplicate removal.

Supplementary Table 1 | Description of metadata fields in the compiled global SAR database.

Column Header	Description
ID	Unique identifier for each SAR record in the database.
Latitude	Latitude of the sampling site (decimal degrees).
Longitude	Longitude of the sampling site (decimal degrees).
Ecosystem	The specific coastal or marine ecosystem type where the core was collected (e.g., mangrove, estuary, continental shelf).
SAR	The measured Sediment Accumulation Rate (mm yr^{-1}).
SAR method	The specific geochronological technique used to quantify the SAR (e.g., ^{210}Pb, ^{137}Cs, ^{14}C).
Year	The year the sediment core was collected, or the publication year of the source literature when the exact sampling date was not reported.
Reference	The original source literature from which the data point was extracted.

2. Feature association and collinearity screening

Each SAR record was spatially linked to thirteen candidate environmental predictors (variable names and data sources are provided in Supplementary Table 2). Water depth was derived from the GEBCO 2026 global bathymetric grid, which offers improved accuracy in nearshore and coastal environments where sediment accumulation is concentrated. Tidal parameters were derived from the FES2022b global tidal atlas, a finite-element hydrodynamic tidal model that assimilates satellite altimetry and tide-gauge observations. The atlas is distributed at 1/30° resolution and includes a coastally extrapolated version in which the developers extend the solution across nearshore regions, providing continuous coverage of coastal cells that are typically unresolved in global tidal products[1]. We used eleven major and shallow-water constituents (M2, S2, N2, K2, K1, O1, P1, Q1, M4, MS4, MN4); the inclusion of the shallow-water constituents captures the non-linear tidal distortion characteristic of shelf and estuarine settings. For each location, a 15-day water-level series was reconstructed at 15-minute intervals using the PyFES prediction library, and mean tidal range was calculated as the difference between the 88.8th and 10.8th percentiles of the series, corresponding to mean high water and mean low water[1–3].

To avoid collinearity among predictors, we examined the Variance Inflation Factor (VIF). This analysis revealed strong collinearity among the tidal variables—mean tidal range, mean high water spring, and mean high water neap (VIF>10). To eliminate this redundancy, we retained mean tidal range as the representative variable and excluded the others. After this screening, all predictors used in the final model exhibited low collinearity (VIF<2), yielding a final set of eleven independent environmental predictors, each corresponding to a distinct physical process governing sediment accumulation (Supplementary Table 2).

Supplementary Table 2 | Data sources of environmental predictors.

Parameters	Sources	Timescale	Spatial resolution	Global coverage	Reference
Relative sea level rise rates	Permanent Service for Mean Sea Level (PSMSL)	Long-term trend	Point-based (nearest tide gauge stations)	Global tide gauge network	[4]
Tropical cyclone effects	Global Cyclone Hazard Frequency and Distribution, v1 by Socioeconomic Data and Applications Center (SEDAC) (1980−2000)	Long-term trend (1980-2000)	2.5 arc-min	Atlantic, Pacific, and Indian Oceans	
Water depth	GEBCO's global gridded bathymetric datasets	Static	15 arc-second	Global ocean and land	
Mean tidal range	FES 2022 Global Tide dataset	Long-term trend	1/30 degree	Global ocean	[1]
Mean high water springs	FES 2022 Global Tide dataset	Long-term trend	1/30 degree	Global ocean	[1]
Mean high water neaps	FES 2022 Global Tide dataset	Long-term trend	1/30 degree	Global ocean	[1]
Total suspended matter	Medium Resolution Imaging Spectrometer satellite data, processed in the framework of the GlobColour project	Long-term trend (2002-2012)	4 km	Global ocean	[3]
Surface current velocity	Bio-ORACLE v2.2	Long-term trend (2010-2020)	0.05 degree	Global ocean	[5,6]
Seafloor current velocity	Bio-ORACLE v2.2	Long-term trend (2010-2020)	0.05 degree	Global ocean	[5,6]
Surface seawater direction	Bio-ORACLE v3.0	Long-term trend (2010-2020)	0.05 degree	Global ocean	[5,6]
Seafloor seawater direction	Bio-ORACLE v3.0	Long-term trend (2010-2020)	0.05 degree	Global ocean	[5,6]

Note: Tropical cyclone effects were set to zero in regions outside major cyclone basins, consistent with the absence of tropical cyclone activity

3. Target Transformation

Observed SAR values span several orders of magnitude and exhibit a strongly right-skewed distribution. Following established practice in sediment-rate modelling[7], we applied a natural log transformation, ln(1 + SAR), to the target variable prior to model fitting. This transformation compresses the extreme right tail, stabilizes the variance, and prevents a small number of high-magnitude observations from dominating the squared-error loss. All model performance metrics reported below (including R^2) are computed in this log space unless otherwise stated; predictions were back-transformed to the original scale (via the inverse expm1 function) where absolute rate values are reported.

4. Model Training and Benchmarking

The final dataset (n = 1,617) was randomly partitioned into a training set (90%) for model fitting and a held-out test set (10%) for independent performance evaluation. To justify the choice of algorithm, we benchmarked the Gradient Boosting Regressor (GBR) against five alternative machine-learning models: Random Forest (RF), eXtreme Gradient Boosting (XGB), Support Vector Regression (SVR), k-Nearest Neighbors (KNN), and a Multilayer Perceptron (MLP). All models were trained and evaluated under identical conditions (log-transformed target, identical train/test split, 5-fold cross-validation), with each model independently tuned via randomized search. Distance- and gradient-based models (SVR, KNN, MLP) were preceded by feature standardization, whereas tree-based ensembles (GBR, RF, XGB), which are invariant to monotonic feature scaling, were not. The three tree-based ensembles clearly outperformed the distance- and kernel-based models and performed comparably to one another (cross-validated R^2 = 0.55–0.57), with differences well within the variability observed across random data partitions (Supplementary Fig. 2). We therefore based the choice among them on generalization behaviour rather than on marginal differences in test scores: GBR showed the smallest gap between training and test performance (0.77 versus 0.60), indicating the least overfitting to the training data. GBR was accordingly selected as the final model (Supplementary Table 3).

Supplementary Table 3 | Cross-validated, training, and held-out test performance of the six benchmarked algorithms: Support Vector Regression (SVR), k-Nearest Neighbors (KNN), Multilayer Perceptron (MLP), Random Forest (RF), eXtreme Gradient Boosting (XGB), and Gradient Boosting Regression (GBR). Cross-validated R^2 is the mean over five folds of the training partition; training and test R^2 are computed on the training and held-out test sets, respectively. All metrics are reported in log space, on the ln(1 + SAR) scale used for model fitting.

	**GBR**	**SVR**	**KNN**	**MLP**	**RF**	**XGB**
Cross-validated R^2	0.55	0.50	0.50	0.49	0.57	0.55
Train R^2	0.77	0.61	1	0.60	0.87	0.81
Test R^2	0.60	0.59	0.54	0.50	0.65	0.63
MSE	0.44	0.45	0.48	0.50	0.42	0.43

5. Hyperparameter Tuning

The GBR architecture was optimized using randomized search with 5-fold cross-validation, followed by a localized grid search around the best configuration. The search space covered the learning rate, maximum tree depth, subsample fraction, minimum samples per leaf, and maximum features per split; the number of boosting stages was governed by early stopping (validation-based, with a patience of 60 iterations). The optimal configuration was: learning rate = 0.01, maximum tree depth = 9, subsample = 0.8, minimum samples per leaf = 1, and maximum features = 0.3. Following this additional hyperparameter optimization, the final tuned GBR achieved $R^2 = 0.85$ on the training set and $R^2 = 0.64$ on the held-out test set (log space; Supplementary Fig. 1).

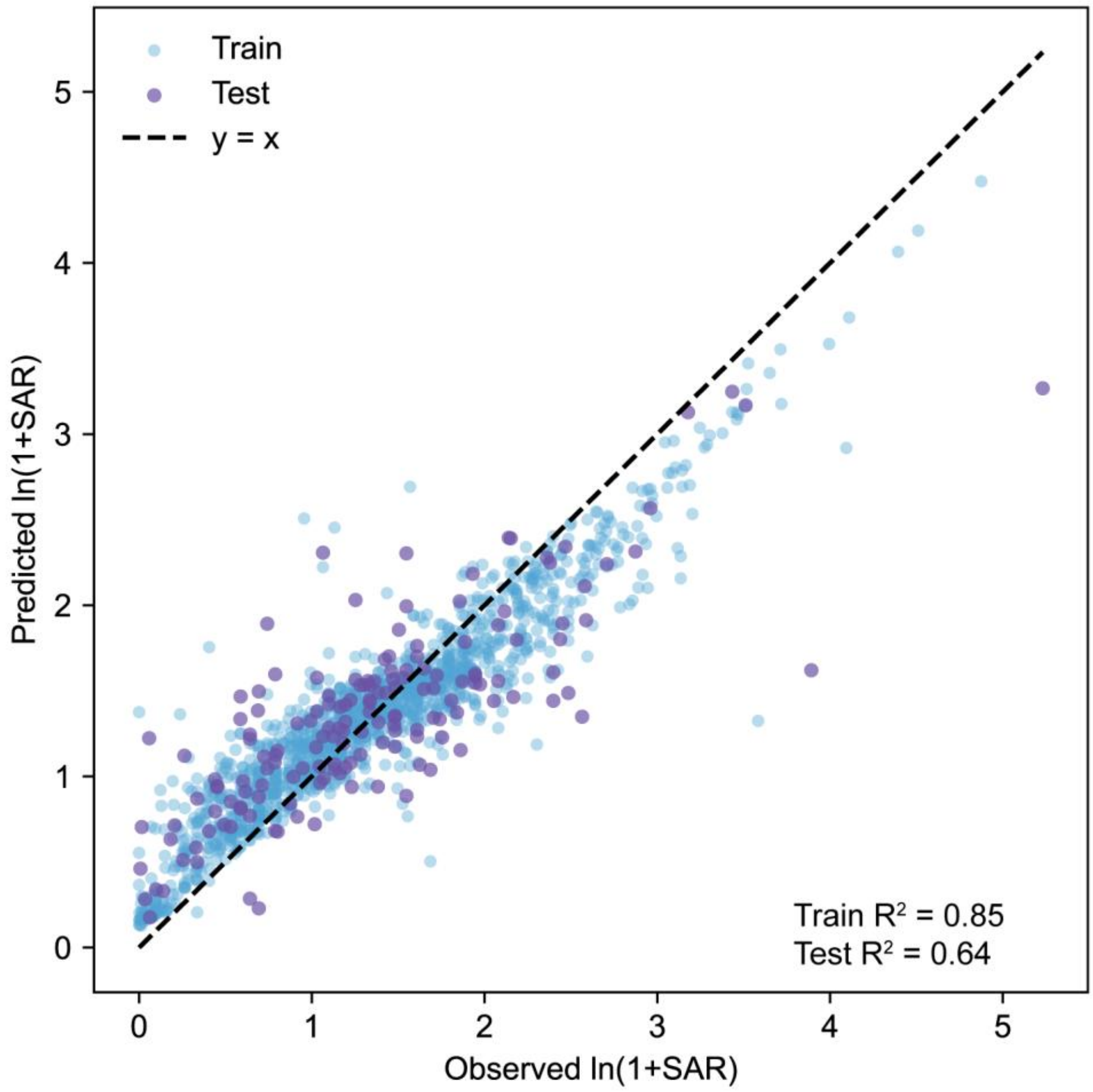

Supplementary Fig. 1 | The relationship between observed ln(1 + SAR) and predicted ln(1 + SAR).

6. Robustness Assessment (Monte Carlo Cross-Validation)

To evaluate model robustness and the stability of the identified environmental drivers under data perturbation, we performed a Monte Carlo cross-validation comprising 100 iterations. In each iteration, the dataset was randomly re-partitioned (90% training /10% testing) and evaluated under the same honest protocol (test set unmodified). The model maintained a mean test R^2 of 0.57 ± 0.07 across all iterations (Supplementary Fig. 2). This distribution-based estimate provides a conservative measure of expected performance and reflects the high spatial heterogeneity of global coastal environments, including partitions containing intrinsically hard-to-predict observations. Critically, the feature-importance ranking remained stable across all 100 iterations (Supplementary Fig. 3). Total suspended matter, water depth, and tropical-cyclone influence—three physically interpretable drivers—emerged as key predictors, together with a longitudinal spatial gradient. This indicates that sediment accumulation is primarily controlled by sediment supply, hydrodynamic setting, and episodic disturbance modulated by a broad-scale east–west spatial structure.

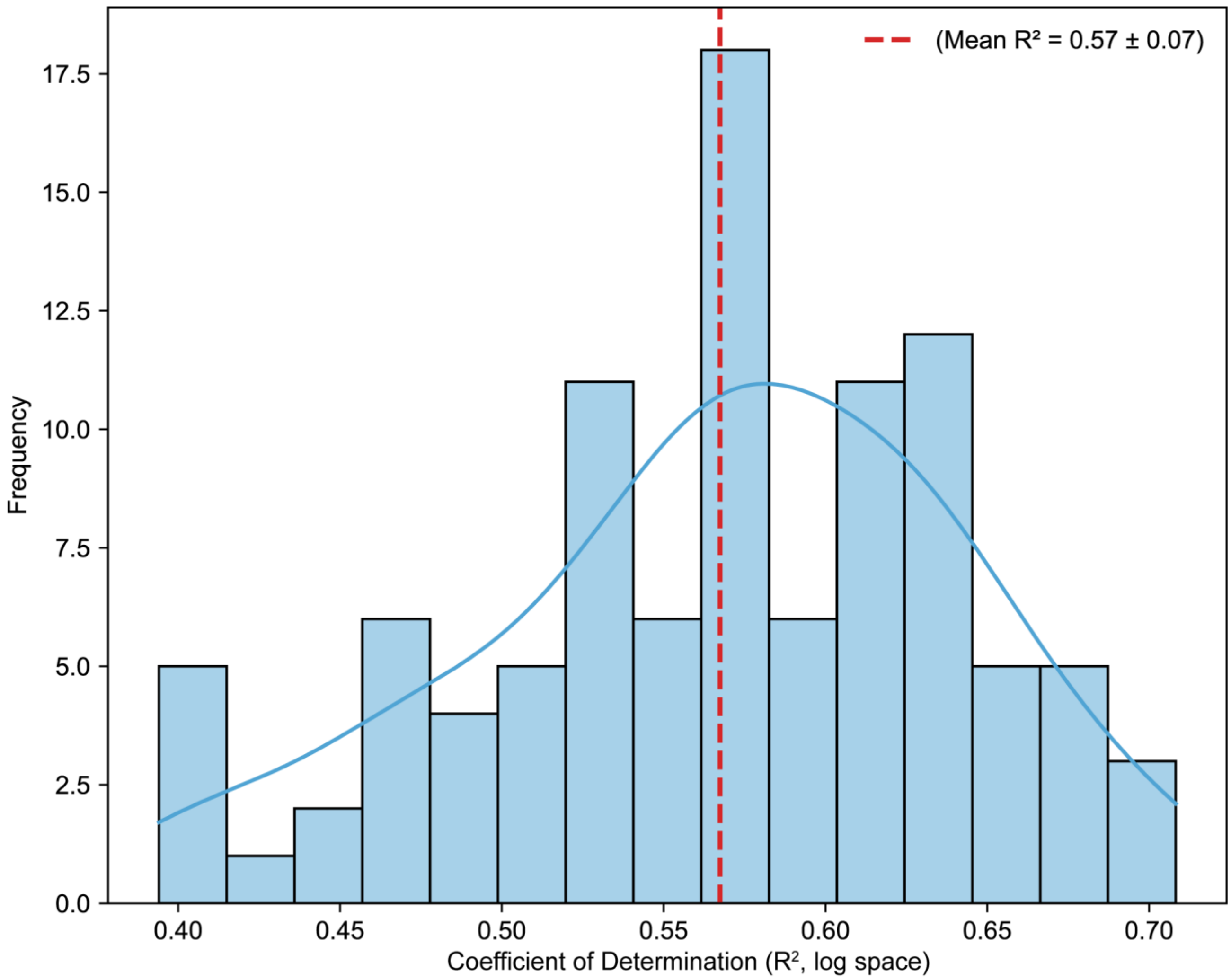

Supplementary Fig. 2 | Distribution of the coefficient of determination (R^2) across 100 random iterations.

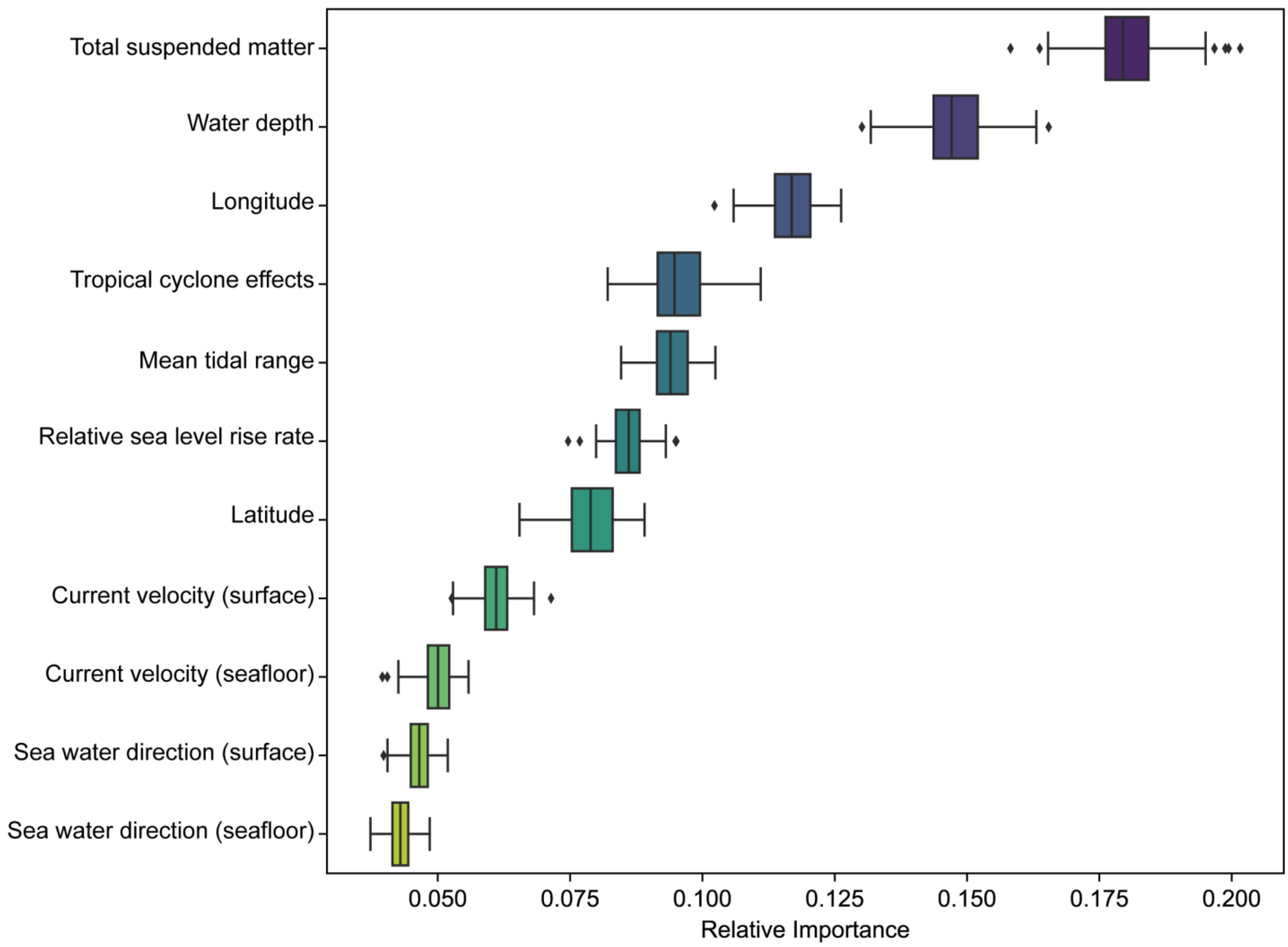

Supplementary Fig. 3 | Feature importance of predictors for coastal SAR across 100 random iterations.

Supplementary Methods 2: SAR prediction uncertainty, geographic transferability, and applicability domain
To characterize the reliability of our global SAR predictions and to identify where they are well constrained by data versus where they rely on extrapolation, we combined three complementary analyses: bootstrap prediction uncertainty, spatial block cross-validation, and two applicability-domain coverage metrics. Together these follow the framework for assessing machine-learning-based spatial predictions of Meyer & Pebesma[8].

1 Bootstrap prediction uncertainty
To quantify the internal predictive uncertainty of the Gradient Boosting Regression (GBR) model, we performed 100 bootstrap iterations of model training. In each iteration, the model was refitted on a sample drawn with replacement from the empirical dataset (n = 1,617) and applied to predict SAR across the global 5-arcminute grid. For each grid cell we computed the mean (μ) and standard deviation (σ) of the 100 predictions, and derived the coefficient of variation ($CV = \sigma/\mu$) as a dimensionless measure of relative uncertainty (Supplementary Fig. 4). Predictions were stable across the majority of temperate and tropical coastlines ($CV < 0.3$ for 98.5% of coastlines), with elevated uncertainty concentrated in data-sparse polar regions.

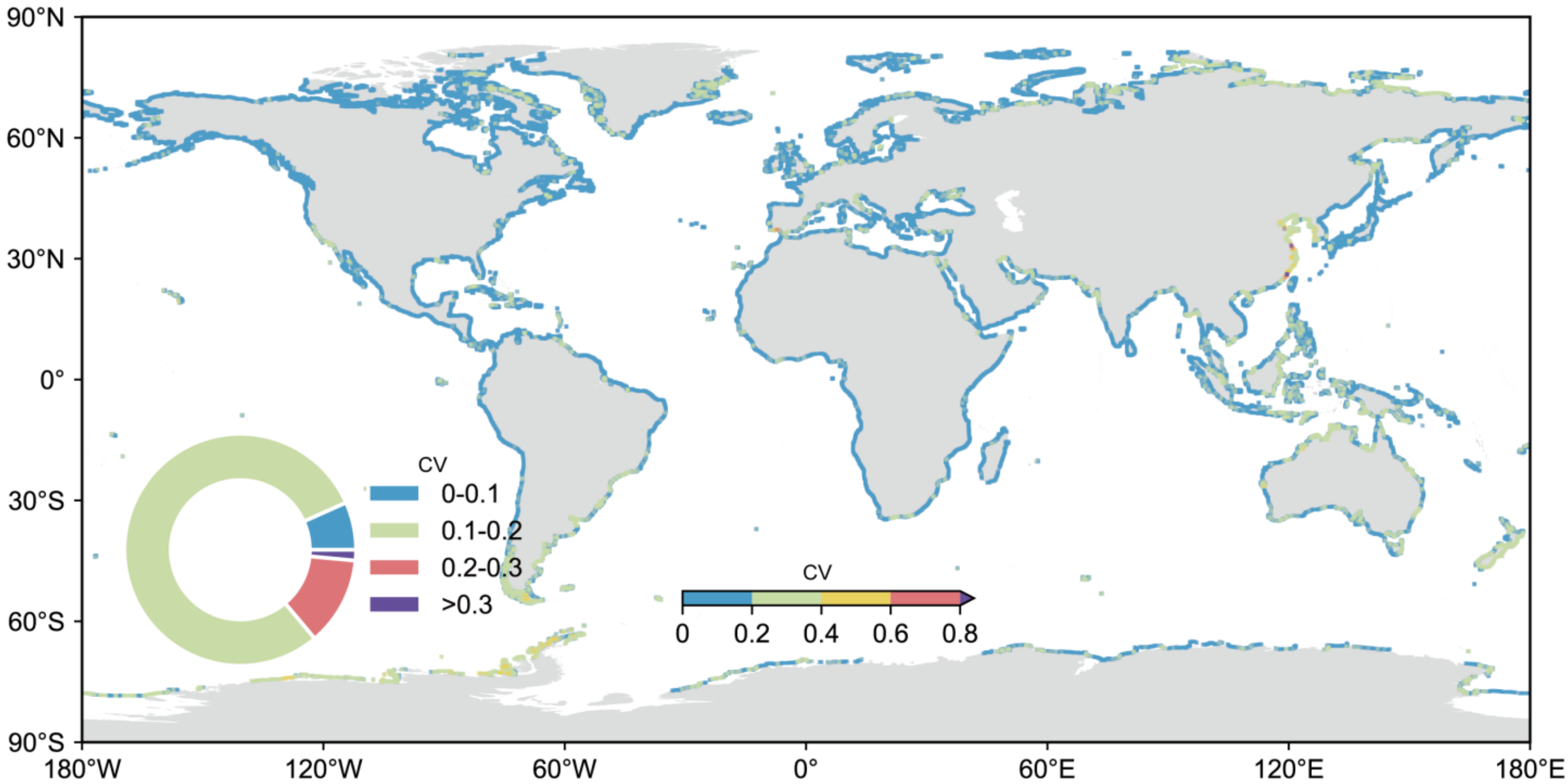

Supplementary Fig. 4 | Spatial uncertainty of the global coastal sediment accumulation rate (SAR) predictions, expressed as the coefficient of variation (CV) across 100 bootstrap iterations. The inset shows the frequency distribution of CV values across all predicted coastal grid cells. Basemap data are from Natural Earth.

2 Spatial block cross-validation

Bootstrap uncertainty quantifies the stability of predictions under resampling but does not test the model's ability to predict geographically unseen regions. We therefore performed spatial block cross-validation using a leave-one-block-out scheme. Sampling locations were assigned to 5° × 5° spatial blocks; each block containing at least 10 observations (42 blocks in total) was held out in turn, with the model retrained on the remaining data and used to predict the held-out block. Predictions were aggregated across all held-out blocks and evaluated in log space.

As expected for spatially autocorrelated data, absolute predictive skill decreased under spatial blocking relative to random 5-fold cross-validation of the final tuned ($R^2 = 0.30$ versus 0.59), confirming that extrapolation of absolute SAR values across regions is inherently limited. The relative spatial ranking of predictions across held-out blocks nonetheless remained moderately preserved (overall Spearman $\rho = 0.51$). Because MCBI is a relative index—defined by the ranking of burial favorability rather than by absolute SAR values—this preserved aggregate ranking supports its intended global-scale application, while confirming that absolute regional SAR values should be interpreted with greater caution.

3 Applicability-domain coverage metrics

To distinguish regions where predictions are supported by environmentally similar training data from those that rely on extrapolation, we computed two coverage metrics for every predicted grid cell. The feature-space distance was defined as the mean Euclidean distance to the five nearest training observations in the standardized environmental-predictor space (the nine environmental predictors, excluding geographic coordinates; standardized using training-set statistics). The geographical distance was defined as the mean great-circle distance to the five nearest training observations. A small feature-space distance indicates that a cell's environmental conditions are represented within the training data, whereas a large geographical distance indicates the absence of nearby local observations (Supplementary Fig. 5, 6).

These two metrics decouple two forms of predictive support that bootstrap uncertainty alone conflates. Regions with small feature-space but large geographical distance are environmentally familiar to the model yet lack local observational validation: their predictions may appear precise (low CV) but should be interpreted with corresponding caution. Regions distant in both senses represent true extrapolation and carry the highest predictive risk. We present these coverage metrics alongside the bootstrap uncertainty (Supplementary Fig. 4–6) to guide interpretation of the global predictions.

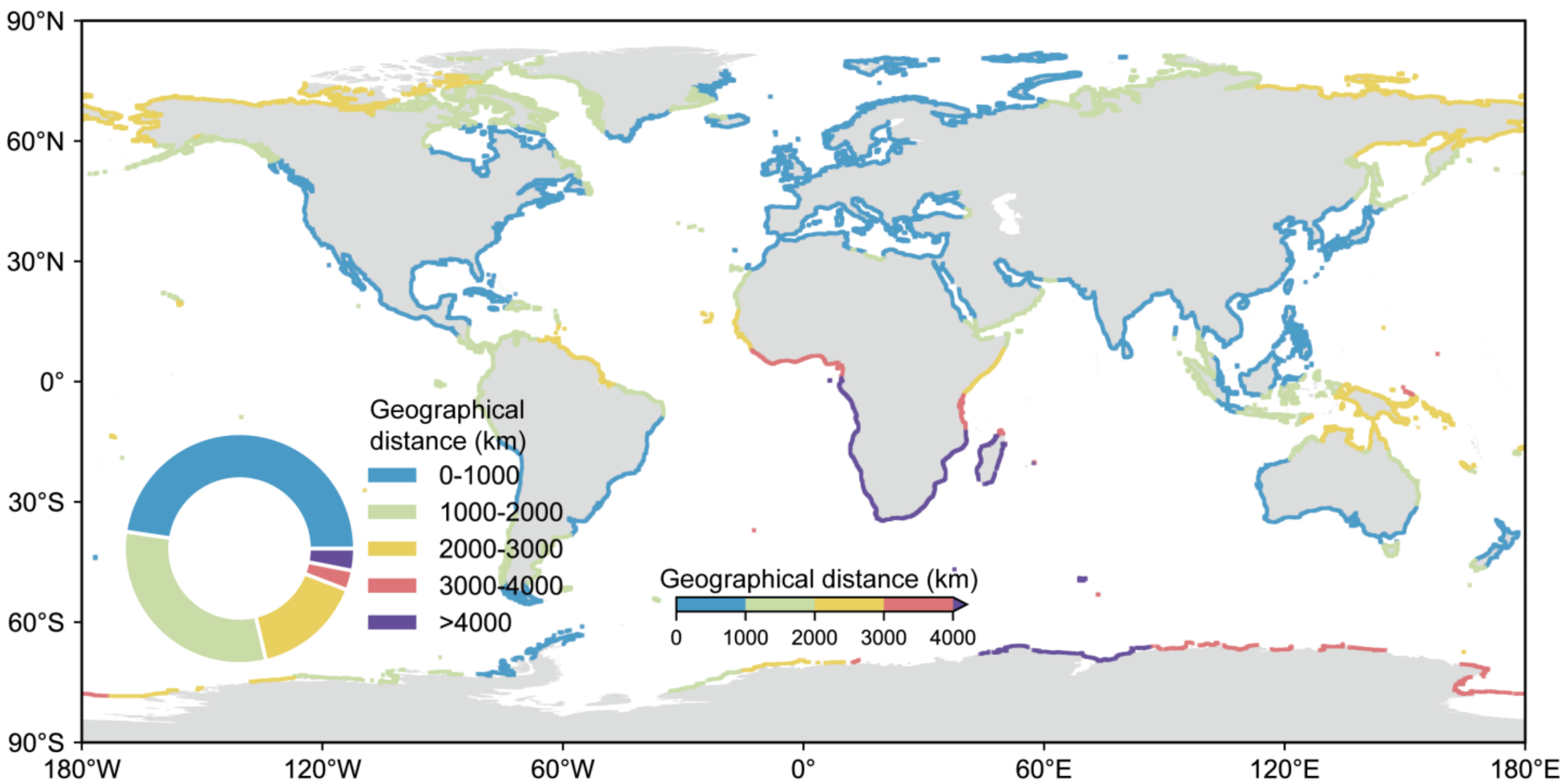

Supplementary Fig. 5 | Spatial distribution of geographical distance for the global coastal sediment accumulation rate (SAR) predictions. The inset shows the frequency distribution of geographical distance across all predicted coastal grid cells. Basemap data are from Natural Earth.

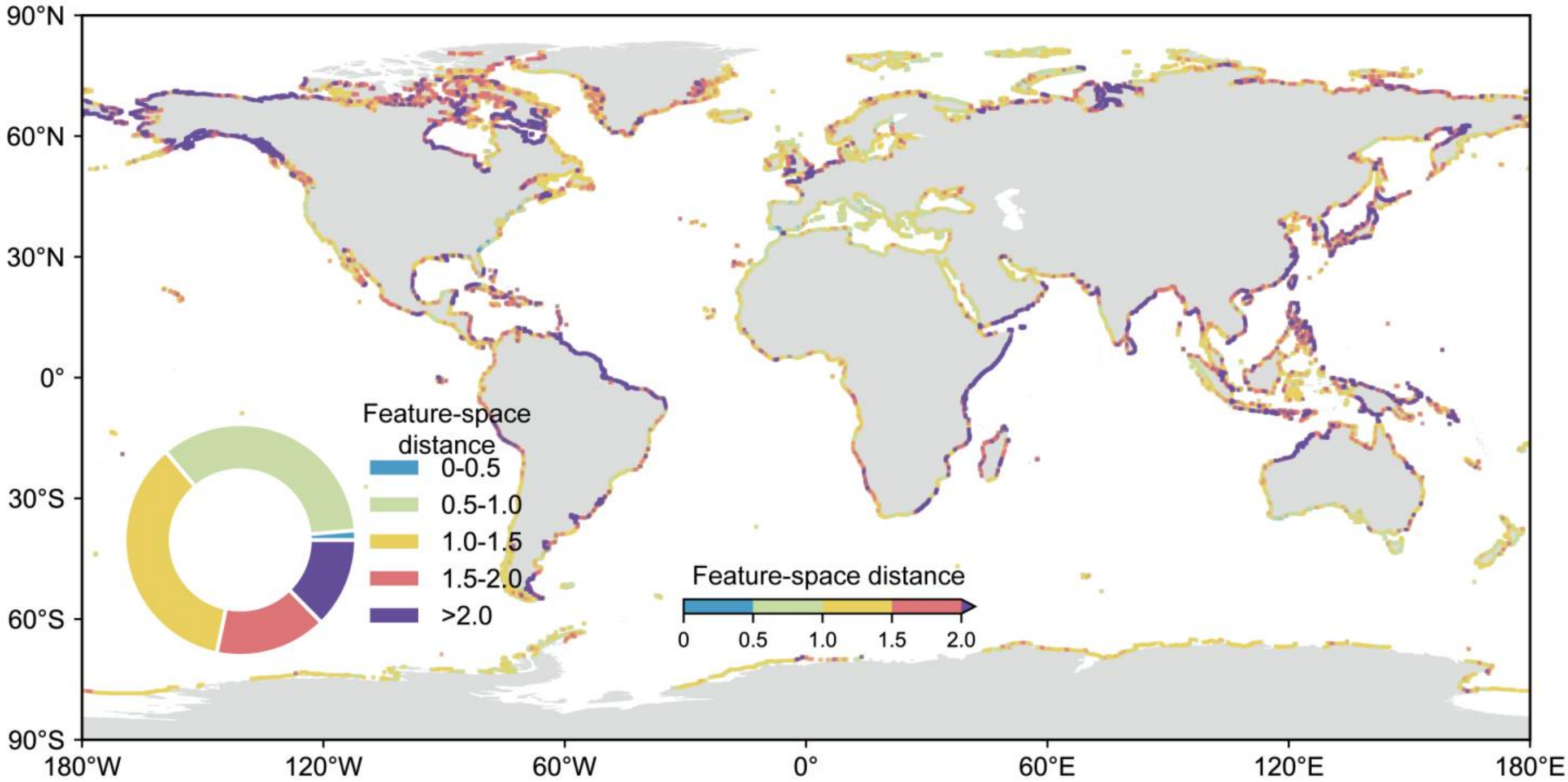

Supplementary Fig. 6 | Spatial Feature-space distance of the global coastal Sediment Accumulation Rate (SAR) predictions. The inset shows the frequency distribution of Feature-space distance across all predicted coastal grid cells. Basemap data are from Natural Earth.

Supplementary Methods 3: Handling of NPP uncertainty and data conservatism

The biological layer draws on the global macroalgal net primary productivity (NPP) estimates of Pessarrodona et al.[9], derived from ecological niche models for three functional groups—red algal turfs, intertidal brown algae, and subtidal brown algae. To avoid double-counting productivity within the brown-algal canopy, we took the maximum of the intertidal and subtidal brown-algal values rather than their sum, and combined this with the red algal turf value to obtain total potential macroalgal NPP for each grid cell.

Because spatially explicit, pixel-wise error bounds are not available for this third-party dataset, we adopted a conservative data-handling protocol to limit the propagation of extreme model artifacts: NPP values were truncated at the 95th percentile, with all values above this threshold reassigned to the 95th-percentile value. The resulting conservative biological supply layer is shown in Supplementary Fig. 7.

We emphasize that this layer represents the maximum potential macroalgal productivity attainable under environmentally suitable conditions, rather than realized, co-occurring biomass; it therefore does not resolve shading interactions between canopy and understory groups where these physically co-occur[10]. This potential-based definition is consistent with the purpose of MCBI as a relative index of burial favorability.

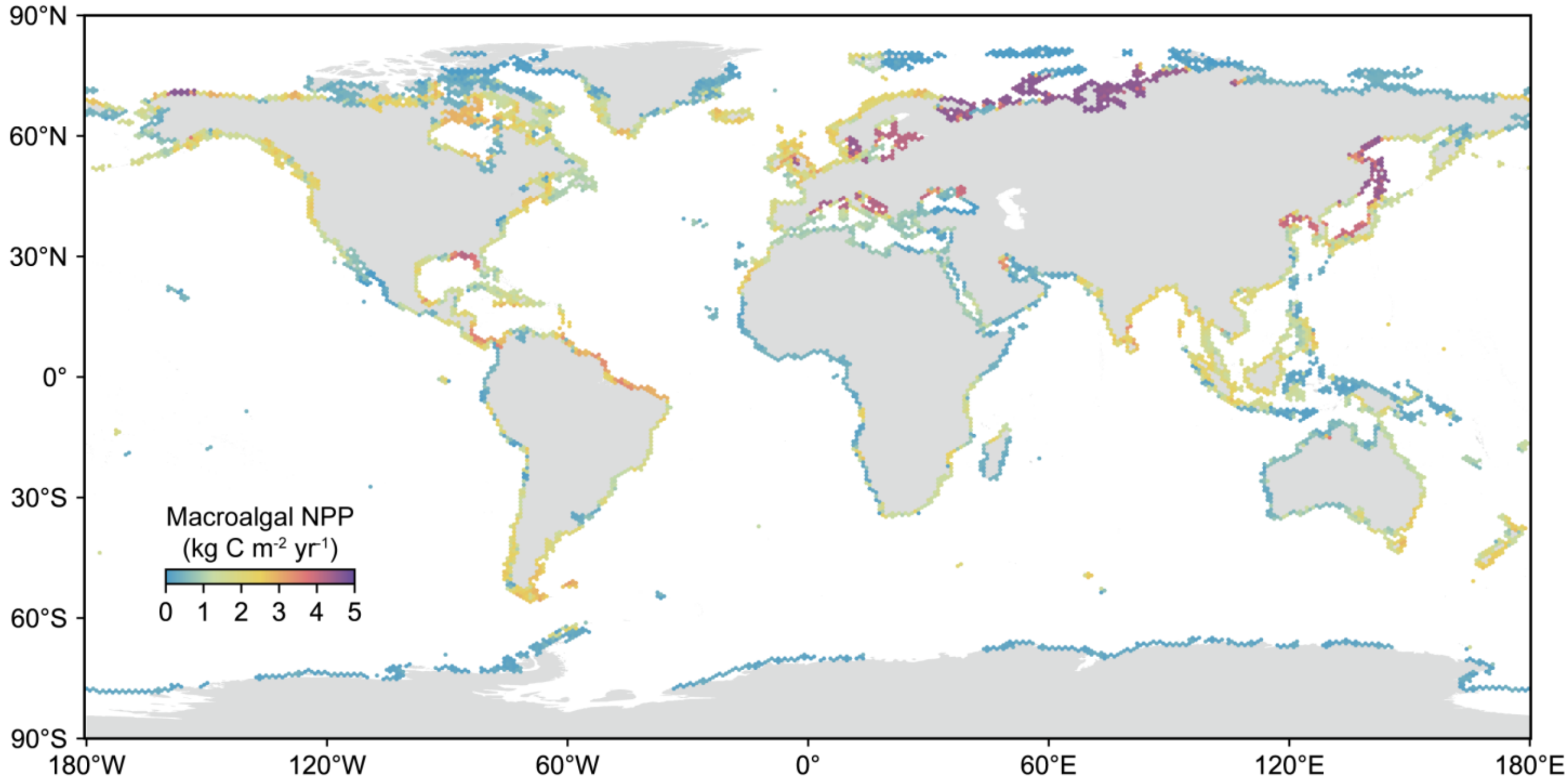

Supplementary Fig. 7 | Spatial distribution of the modeled global macroalgal Net Primary Productivity (NPP). Basemap data are from Natural Earth.

Supplementary Methods 4: Centennial-scale MCBI

To extend the MCBI toward longer-term carbon storage, we incorporated the 100-year organic-carbon transfer efficiency (T_{eff}) of Bradley et al.[11], which quantifies the fraction of organic carbon deposited at the sediment–water interface that survives 100 years (0.1 ka) of post-depositional degradation (their $T_{eff,age}$, SWI→0.1 ka). This transfer-efficiency field is derived from a spatially resolved global reaction-transport model in which organic-carbon degradation is represented mechanistically.

The global T_{eff} field, distributed at 0.25° resolution, was sampled to our 5-arcminute coastal grid by nearest-neighbour assignment. For each coastal grid cell we defined the centennial-scale MCBI as:

$$\text{Centennial-scale MCBI} = \text{MCBI} \times (T_{eff} / 100)$$

where T_{eff} is expressed as a percentage (0–100%). This weights the physical burial favorability captured by MCBI by the modelled long-term preservation efficiency at each location, yielding an index that reflects both the capacity for burial and the durability of the buried carbon.

The centennial-scale index remained strongly rank-correlated with the base MCBI across all coastal grid cells (Spearman $\rho = 0.76$, $p < 0.001$, $n = 82{,}134$; Supplementary Fig. 8), confirming that incorporating transfer efficiency preserves the principal spatial patterns of burial favorability while additionally resolving differences in long-term preservation.

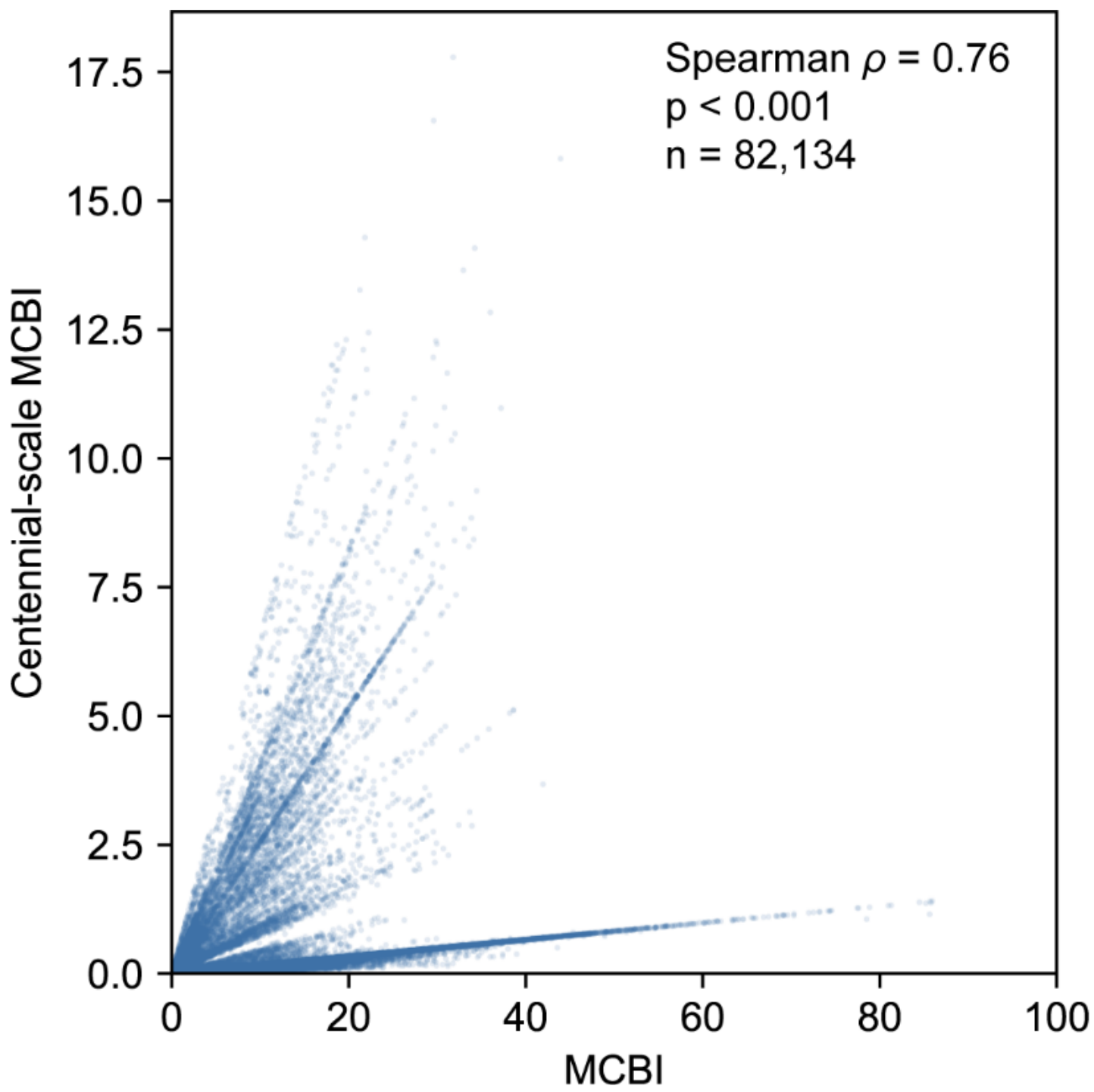

Supplementary Fig. 8 | Relationship between the MCBI and the centennial-scale MCBI across all coastal grid cells (Spearman $\rho = 0.76$, $p < 0.001$, $n = 82{,}134$).

Supplementary Methods 5: Sensitivity analyses

The primary Macroalgal Carbon Burial Index (MCBI) quantifies the geophysical boundary conditions for burial; the realized net sink is additionally modulated by biological loss pathways prior to ultimate sequestration. To explore the spatial heterogeneity introduced by temperature-dependent microbial decomposition—without over-parameterizing the dimensionless index—we conducted a theoretical sensitivity analysis.

Large-scale field evidence (Filbee-Dexter et al.[12], across 35 Northern Hemisphere sites) shows that macroalgal detritus decomposition is strongly temperature-dependent. However, those data were fitted with a linear mixed model over a narrow temperature range (~5–20 °C); extrapolating a linear relationship to sub-zero polar and >30 °C tropical waters would produce biologically unrealistic artifacts (e.g., negative decomposition rates). We therefore represented the global thermodynamics of remineralization using Arrhenius-type kinetics, applying a standard biological temperature coefficient ($Q_{10} = 2.0$)—i.e., decomposition rate doubling per 10 °C—consistent with global soil and marine sediment organic-carbon decomposition models[13,14].

We defined a relative thermal retention factor *f*, bounded between 0 and 1, as an exponential decay driven by sea bottom temperature (SBT; 5-arcminute, Bio-ORACLE[5,6]; Supplementary Fig. 9). To respect mass conservation (retained carbon cannot exceed initial production), a freezing baseline ($T_{ref} = 0$ °C) was set as the thermodynamic floor: at or below 0 °C, remineralization is assumed minimal and retention maximal ($f = 1$). SBT values below 0 °C were clamped to zero, giving

$$f = 2.0^{-\frac{\max(SBT,0)}{10}}$$

The temperature-adjusted MCBI (MCBI × *f*) was compared with the original index (main-text Fig. 2c–e). The two remained strongly correlated (Spearman $\rho = 0.86$, $p < 0.001$), with the adjustment concentrated in the equatorial zone and high-latitude hotspots largely unaffected, indicating that the relative ranking of burial hotspots is robust to this thermal modifier. Because rapid burial associated with high SAR can shield detritus from temperature-driven remineralization—a trade-off that cannot yet be robustly parameterized at the global scale—we retain the unadjusted MCBI for the main analyses and present the temperature adjustment as a sensitivity analysis.

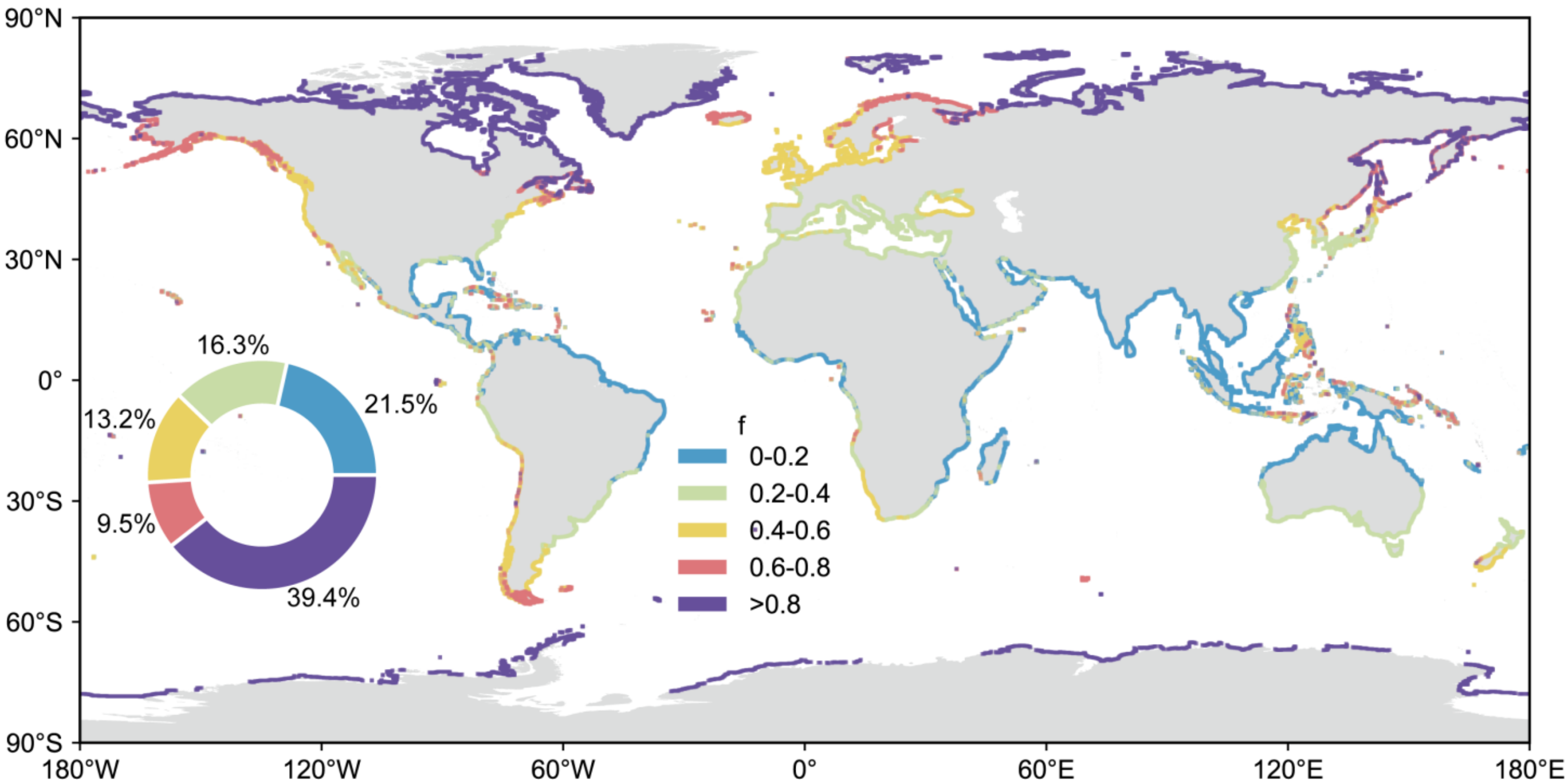

Supplementary Fig. 9 | Spatial sensitivity analysis of the theoretical thermal retention factor (*f*) for macroalgal carbon. Basemap data are from Natural Earth.

Supplementary Methods 6: Automated workflow for fractal dimension (D) calculation

To quantify the geometric complexity of global coastlines, we developed an automated computational workflow based on the standard box-counting method (Supplementary Fig. 10). High-resolution coastline vector data (1:10m scale) were acquired from the Natural Earth dataset. To facilitate spatially explicit analysis, we generated a global tessellation grid (10° ×10°) using ArcGIS Pro 3.0. The global coastline was clipped and segmented according to this grid system. To ensure consistent spatial calculations, all vector segments were projected to the Web Mercator coordinate system (EPSG:3857) using Python scripting prior to analysis.

The vector segments were subsequently rasterized to enable box-counting calculations. To determine the optimal raster resolution that balances computational efficiency with geometric fidelity, we conducted a rigorous resolution sensitivity analysis (Supplementary Fig. 11). We iteratively calculated the mean fractal dimension across a spectrum of raster cell sizes ranging from 200 m to 4000 m (step size = 100 m). Our analysis revealed a convergence threshold at a cell size of 2200 m. Below this threshold (i.e., finer resolutions), the calculated fractal dimension stabilized, indicating that further increases in resolution yielded negligible gains in geometric detail capture. Conversely, coarser resolutions (cell sizes > 2200 m) resulted in a marked loss of coastline irregularity features (undersampling). Consequently, we selected 2200 m as the optimal rasterization resolution for the final global assessment.

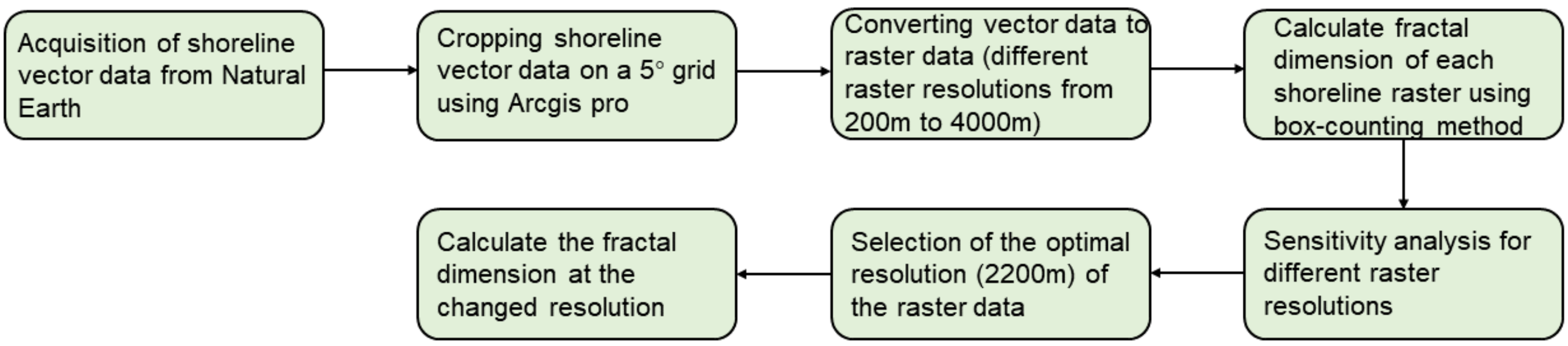

Supplementary Fig. 10 | Schematic workflow for the calculation of coastline fractal dimension (D).

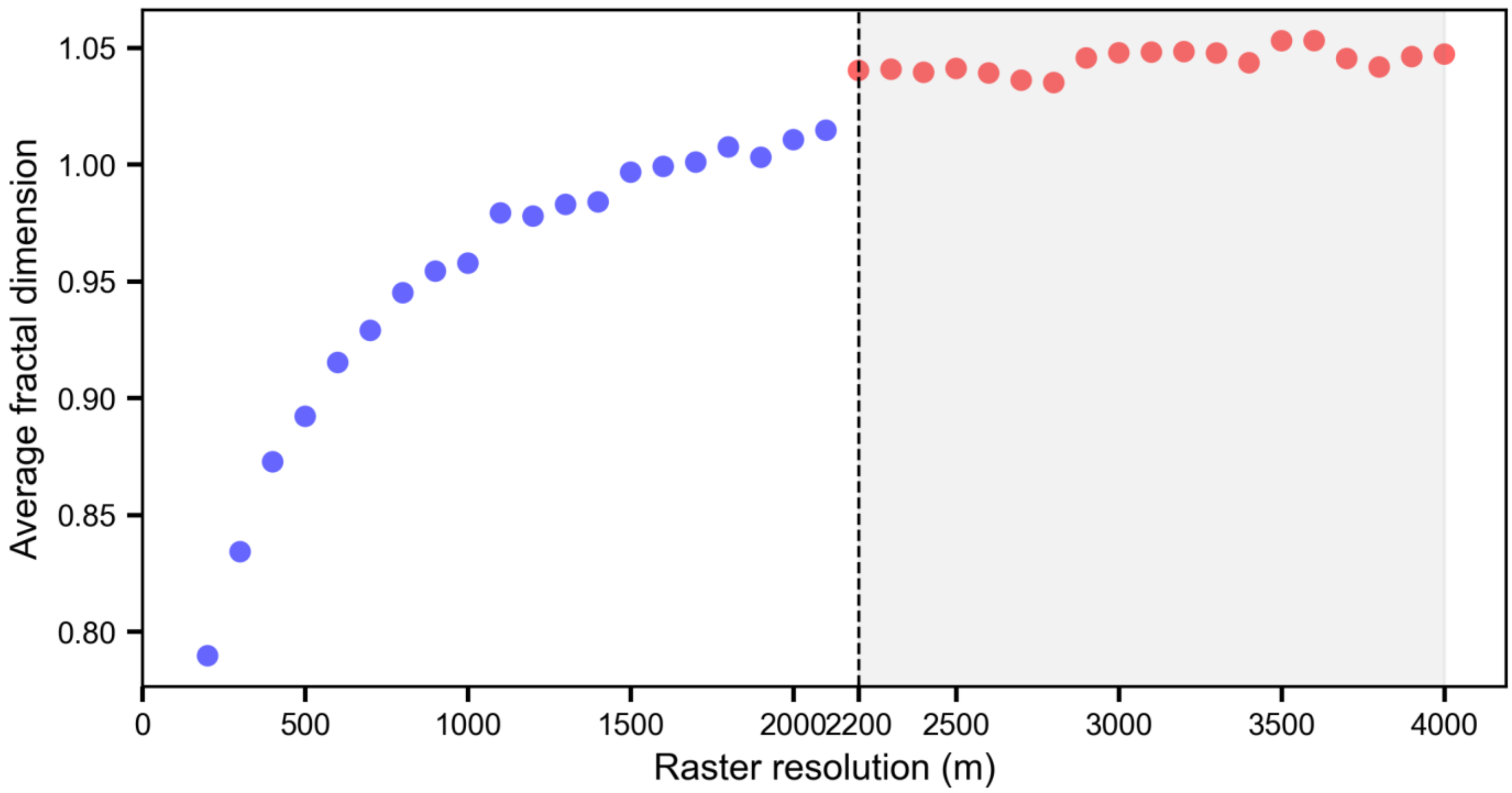

Supplementary Fig. 11 | Sensitivity analysis of fractal dimension estimates to raster resolution.

Supplementary Methods 7: Sensitivity analysis of binning strategies for coastline complexity

The primary rationale for adopting a binning strategy in our analysis of coastline complexity is to statistically extract the underlying macro-geomorphological signal from the inherent noise of global-scale gridded datasets. Ecological and geophysical data derived from high-resolution spatial models often exhibit significant dispersion due to localized heterogeneity and the multitude of interacting micro-scale factors. Binning is a widely accepted and standardized statistical technique in macroecology and geoscience designed to smooth out these stochastic local fluctuations. By aggregating data within defined intervals, this approach reduces the influence of localized noise, thereby allowing for a more stable and robust quantification of the dominant, broad-scale relationship between coastline fractal dimension (D) and MCBI.

To ensure that our findings are robust and not artifacts of a specific data-grouping choice, we conducted a comprehensive sensitivity analysis testing various bin widths and grouping intervals. Specifically, we evaluated the robustness of the linear regression across a range of bin numbers (from 5 to 15 bins) using two distinct statistical approaches:

1. Equal-interval binning: The range of fractal dimensions was divided into equal-width intervals (the approach utilized in the main text for its straightforward interpretability of the x-axis).

2. Quantile (equal-frequency) binning: The dataset was divided such that each bin contained an equal number of grid cells, thereby accounting for potential biases caused by uneven data distribution along the x-axis.

For each iteration, we calculated the coefficient of determination (R^2) and the corresponding p-value to assess statistical significance. The results of this sensitivity analysis are presented in Supplementary Fig. 12.

The analysis demonstrates that while the exact R^2-value fluctuates slightly depending on the specific number of bins and the grouping method, it generally remains robust at approximately 0.50 or higher across the vast majority of iterations. These results indicate that the positive relationship between coastline complexity and MCBI is generally robust across alternative binning strategies, although its statistical strength varies with the specific binning parameters.

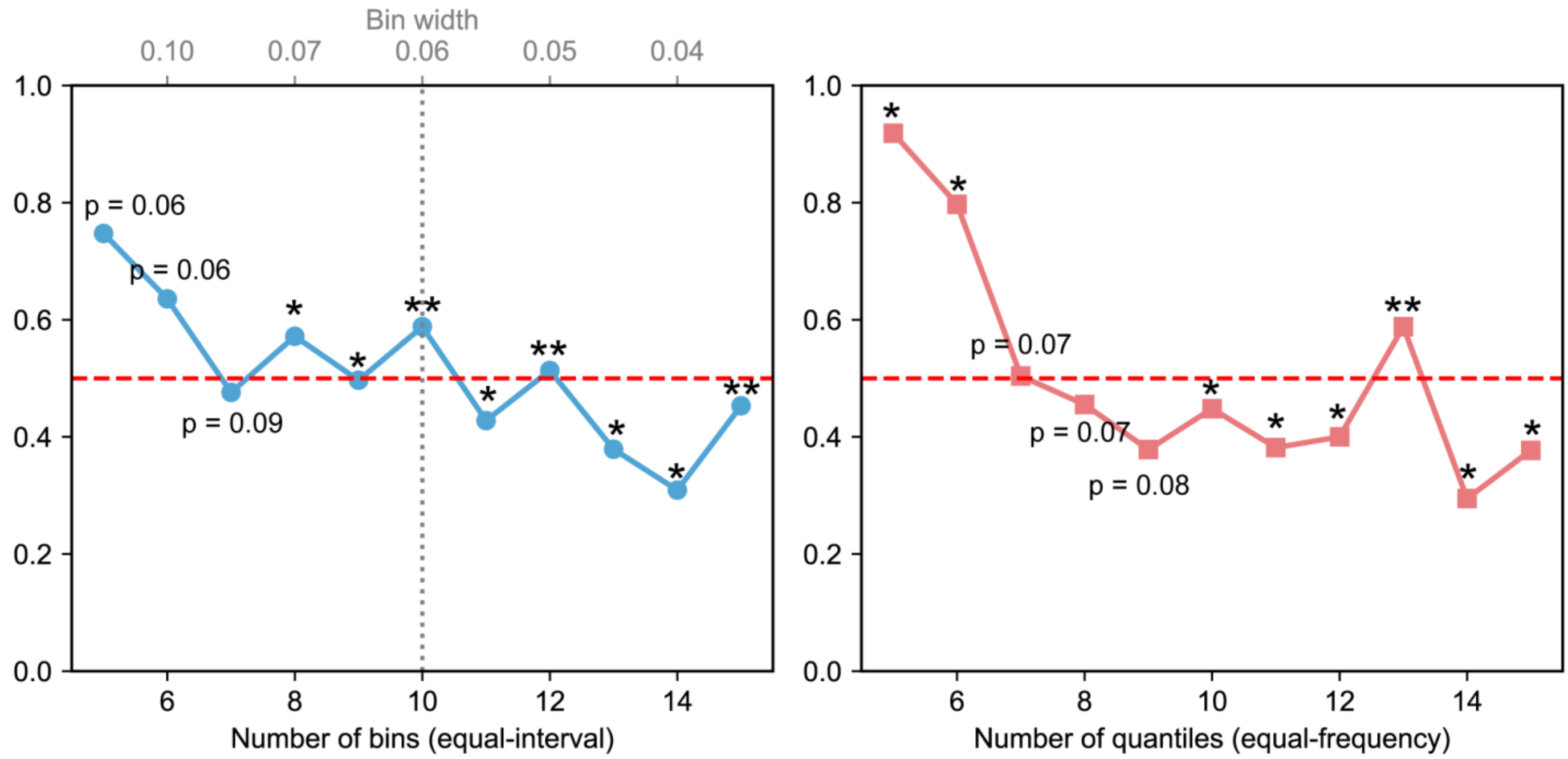

Supplementary Fig. 12 | Sensitivity analysis of the correlation between coastline fractal dimension (D) and MCBI to different binning strategies. The robustness of the linear regression was evaluated across varying numbers of bins (ranging from 5 to 15) using two distinct grouping approaches: a, Equal-interval binning (left), and b, Quantile (equal-frequency) binning (right). The y-axis represents the coefficient of determination (R^2) for each iteration.

Supplementary Methods 8: Assessment of conservation coverage and future scenario modeling

To evaluate the extent to which MPAs currently overlap with coastlines favourable for macroalgal carbon burial, we stratified the global MCBI into five quantile-based categories: Extremely Low (0–20th percentile), Low (20–40th), Medium (40–60th), High (60–80th), and Extremely High (80–100th). We then quantified the protection rate for each category using a value-weighted rather than a simple area-based approach. Specifically, using ArcGIS Pro 3.0 and Python, we summed the MCBI values falling within existing MPA boundaries for each quantile category and divided this by the total MCBI of that category. This provides a functional assessment of how much burial favourability is captured by the existing network, avoiding biases associated with grid-cell area variation across latitudes (Supplementary Table 4).

To assess how the spatial allocation of new protection would affect the burial favourability captured by the network, we conducted a scenario analysis anchored on the "30×30" target (protecting 30% of the ocean by 2030)[15]. The analysis was restricted to the 82,134 grid cells of MCBI. Of these cells, 15,720 (19.1%) currently overlap with MPAs; reaching 30% coverage (24,640 cells) would require protecting a further 8,920 cells. We evaluated the mean MCBI of the resulting 24,640-cell network under three allocation strategies:

(i) Strategic expansion (high-value targeting): the 8,920 unprotected cells with the highest MCBI are selected, representing the upper bound of achievable performance.

(ii) Random expansion (carbon-blind): 8,920 unprotected cells are drawn at random, repeated over 1,000 iterations, with the mean and 95% confidence interval reported. This represents a continuation of current planning practice, in which burial favourability is not considered.

(iii) Low-value expansion: the 8,920 unprotected cells with the lowest MCBI are selected, representing a theoretical lower bound.

Mean MCBI was computed across the full expanded network (existing plus newly protected cells) in all three cases, so that the strategies are compared at identical spatial cost (Supplementary Table 5). Note that the 30% threshold applied here is areal, consistent with the 30×30 framing, and is distinct from the MCBI-weighted protection rates reported in the first part of this section and in main-text Fig. 4c, d.

Supplementary Table 4 | Statistical summary of MCBI quantile segments and assessment of current conservation coverage.

MCBI segment	Percentile range	MCBI range	Total MCBI	Total MCBI protected by MPAs	Protection rate
Extremely Low	Below 20th percentile	0 – 1.13	8511.53	2037.83	23.9%
Low	20th to 40th percentile	1.13 – 3.26	33114.46	6003.41	18.1%
Medium	40th to 60th percentile	3.26 – 8.07	92995.45	15839.47	17.0%
High	60th to 80th percentile	8.07 – 13.80	177624.03	25342.81	14.3%
Extremely High	Above 80th percentile	13.80 – 85.93	355787.16	60910.73	17.1%

Supplementary Table 5 | Projected average MCBI of the conservation network under future spatial planning scenarios.

Protection Scenarios	Average MCBI (current)	Average MCBI (2030)
Strategic expansion	7.0	13.5
Random expansion	7.0	7.5 [7.45 7.57]
Low-value expansion	7.0	4.6

Supplementary Methods 9: Literature screening and compilation of the SAR database

To improve the transparency of the literature screening and data compilation process described in the Methods, we summarized the workflow using a PRISMA flow diagram (Supplementary Fig. 13). The Web of Science search was conducted in July 2025 and covered studies published during the 2020–2025 period. The search identified 9,986 records, all of which were screened for eligibility. Studies were considered eligible for SAR data extraction if they reported empirically measured sediment accumulation rates (SAR), provided sufficient geographic information to locate the sampling sites, contained extractable quantitative SAR values, and were accessible in full text for data extraction. Studies that did not meet these criteria were excluded during screening. A total of 46 studies were retained for SAR data extraction. All records were screened by two reviewers according to these eligibility criteria, without the use of automated screening tools. Data extraction was subsequently divided between the two reviewers, and no automated data extraction tools were used. All extractable empirical SAR measurements meeting the eligibility criteria were collected from each included study, including measurements from multiple sampling sites where available.

SAR records extracted from these studies were combined with historical SAR records obtained from two sources. For Restrepo et al.[7], historical records were reconstructed by tracing the original studies cited therein, and only records for which quantitative SAR values and associated geographic information could be reliably retrieved were included. For Wang et al.[16], SAR records were obtained directly from the published dataset. The combined dataset was subjected to quality control and duplicate removal, resulting in a final global database of 1,617 unique SAR records. The complete literature screening and database compilation workflow is summarized in Supplementary Fig. 13.

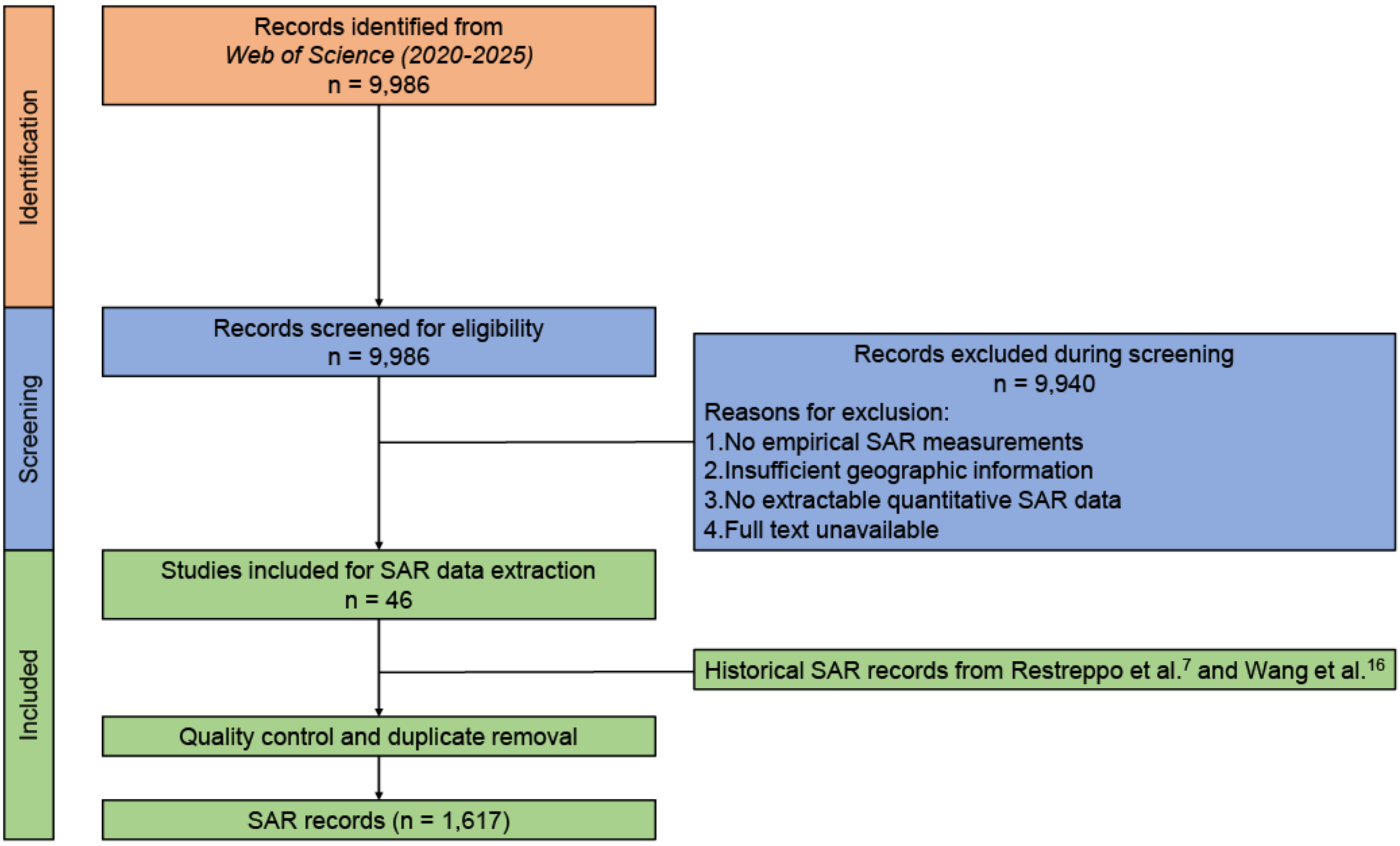

Supplementary Fig. 13 | PRISMA flow diagram of the literature screening and compilation of the global sediment accumulation rate (SAR) database. Records identified from Web of Science for 2020–2025 were screened according to predefined eligibility criteria. Eligible studies were used for SAR data extraction and integrated with historical SAR records from Restreppo et al.[7] and Wang et al.[16]. The combined dataset was subjected to quality control and duplicate removal to generate the final global SAR database.

Supplementary Results 1: The remaining global regions with high MCBI

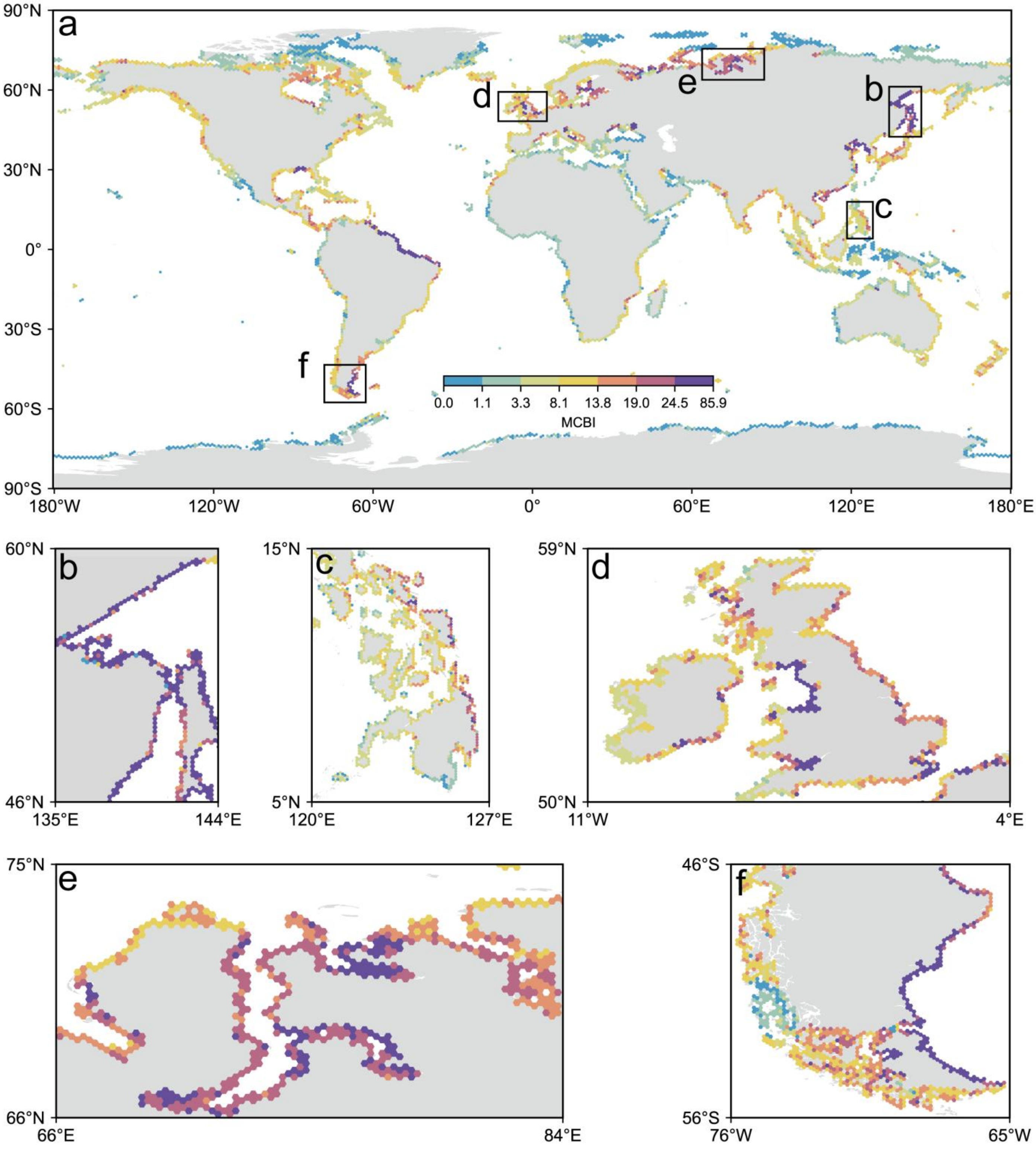

Supplementary Fig. 14 | Regional distributions of MCBI in representative high-value areas. a Global MCBI distribution, with boxes indicating the regions shown in **b–f**. **b** The Okhotsk–Sakhalin region, Russia. **c** Eastern coast of the Philippines. **d** Coastal United Kingdom. **e** Kara Sea coast, Russia. **f** The southern Patagonian fjords, Chile. Basemap data are from Natural Earth.

Supplementary Results 2: Latitudinal distributions of NPP, SAR, and MCBI

To examine how the biological and geophysical components combine across latitude, we computed zonal means of macroalgal NPP, predicted SAR, and MCBI (Supplementary Fig. 15).

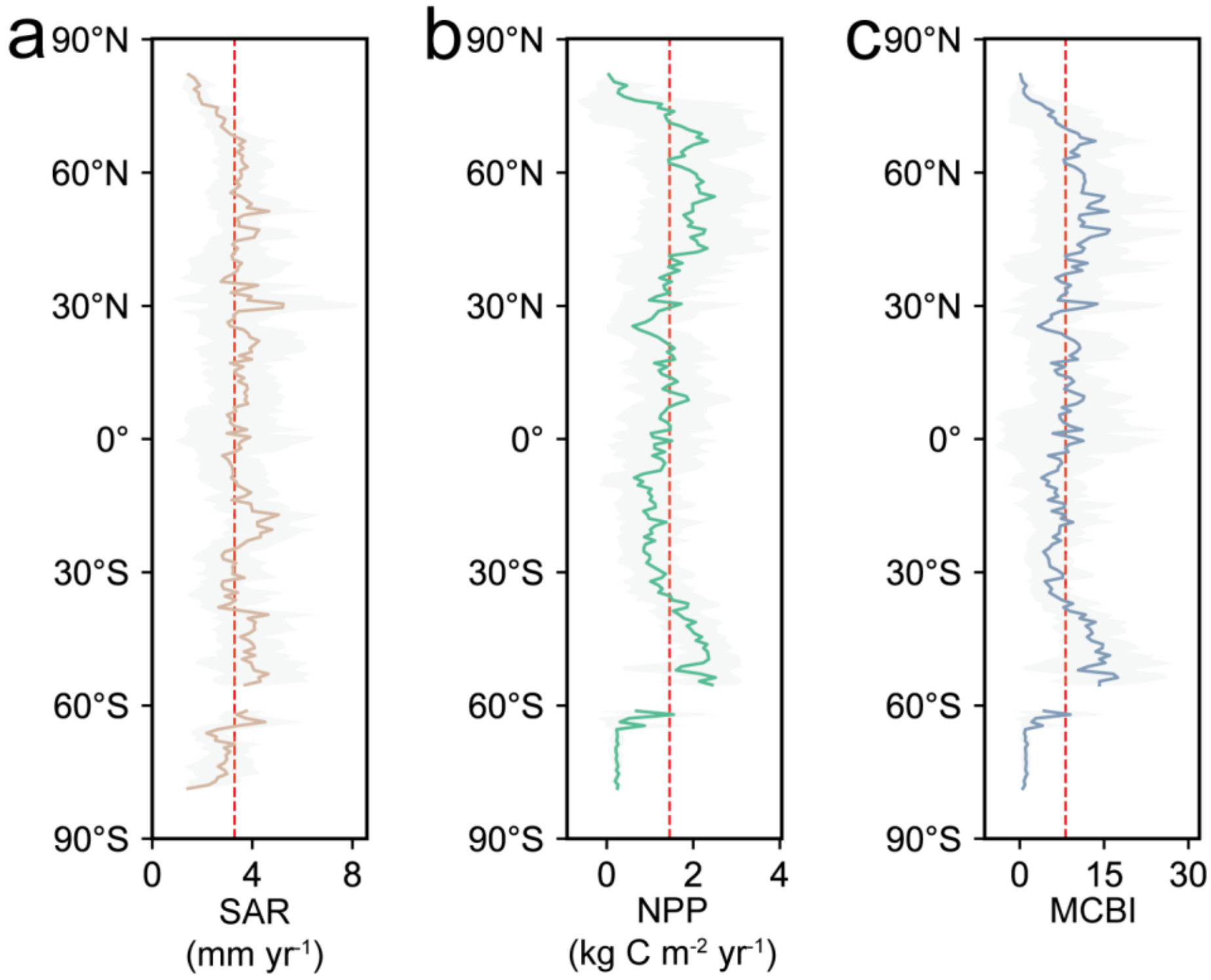

Supplementary Fig. 15 | Latitudinal distributions of the components of MCBI. a Zonal mean predicted sediment accumulation rate (SAR)**. b** Zonal mean macroalgal net primary productivity (NPP)**. c** Zonal mean MCBI. Solid lines show the mean of all valid coastal grid cells within each latitudinal band; shading indicates ±1 standard deviation; red dashed lines denote the global mean.

Supplementary Results 3: Habitat-specific assessment of burial favorability for brown algal forests and red algal turfs

Using habitat-specific net primary productivity (NPP) data, we evaluated MCBI separately for brown algal forests and red algal turfs, in order to delineate the distinct hotspot regions associated with each functional group (Supplementary Fig. 16-19). Note that NPP was normalized independently for each habitat type to maximize spatial contrast within that habitat. Consequently, these habitat-specific MCBI values characterize relative spatial variability within a given functional group only; they do not permit quantitative comparison of absolute magnitude between brown algal forests and red algal turfs.

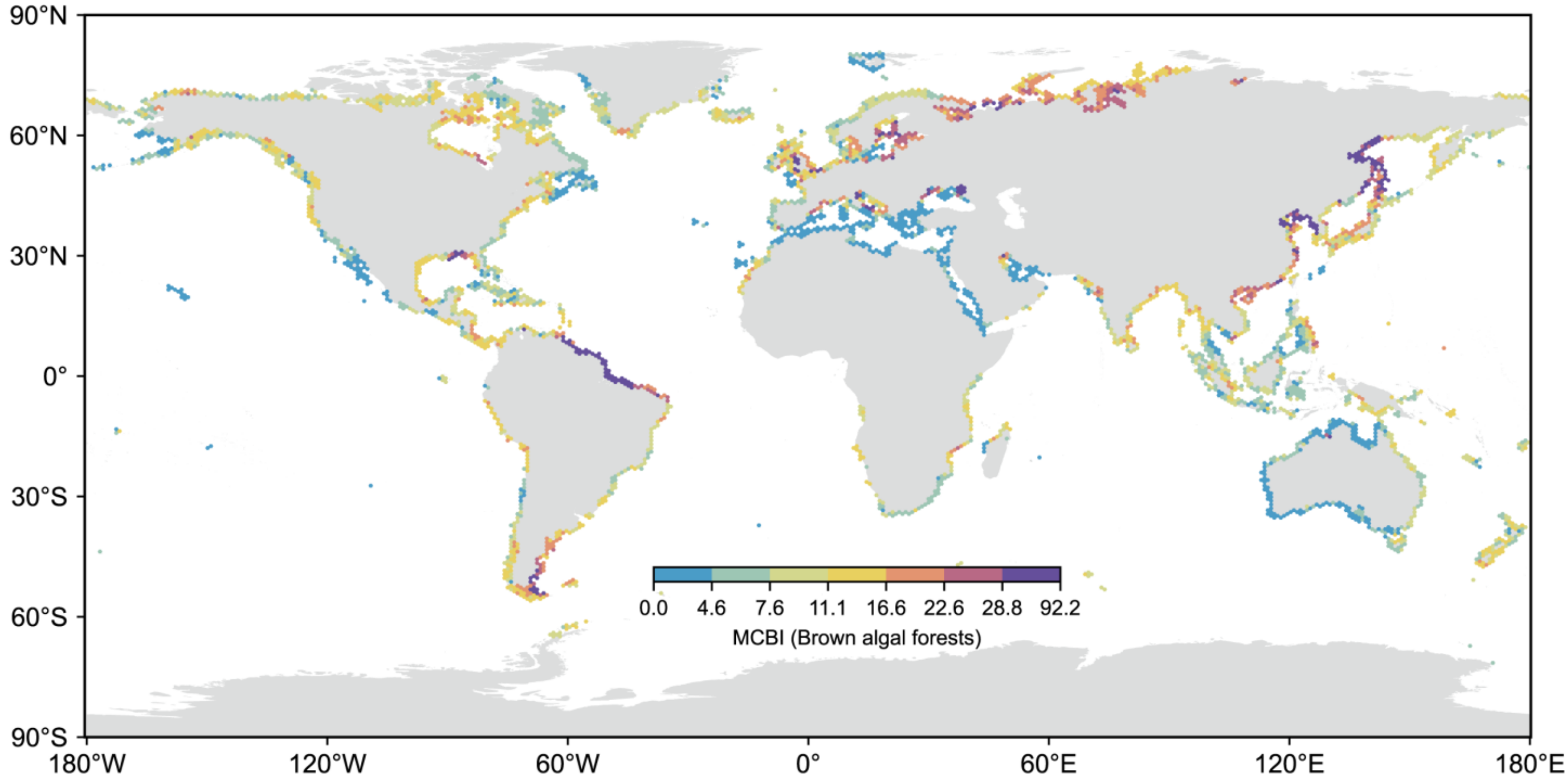

Supplementary Fig. 16 | Global distribution of MCBI for brown algal forests. Basemap data are from Natural Earth.

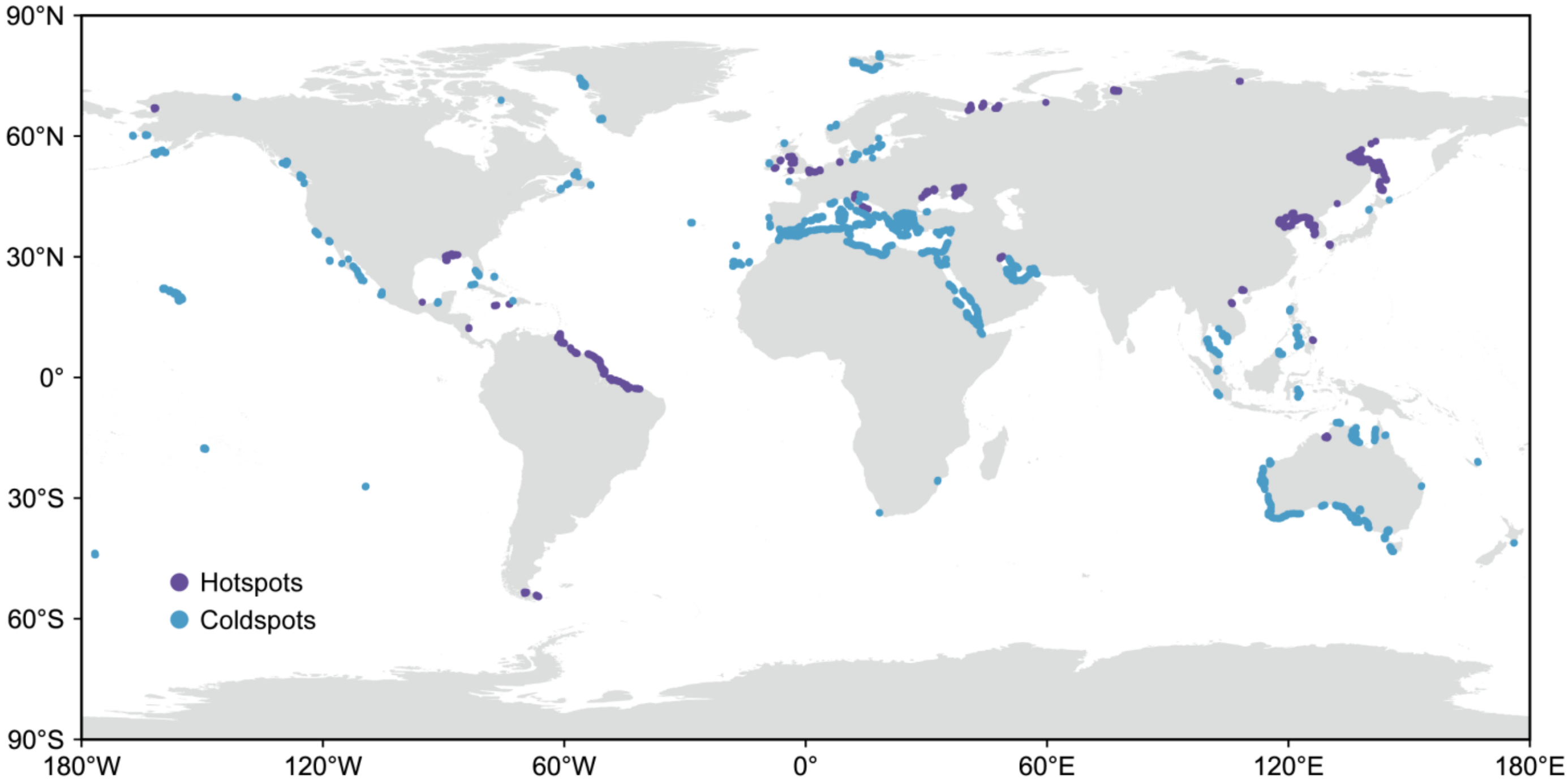

Supplementary Fig. 17 | Hotspots and coldspots of MCBI for brown algal forests, identified as co-hotspots and co-coldspots of brown-algal NPP and SAR (Getis-Ord Gi*; 95% confidence level). Basemap data are from Natural Earth.

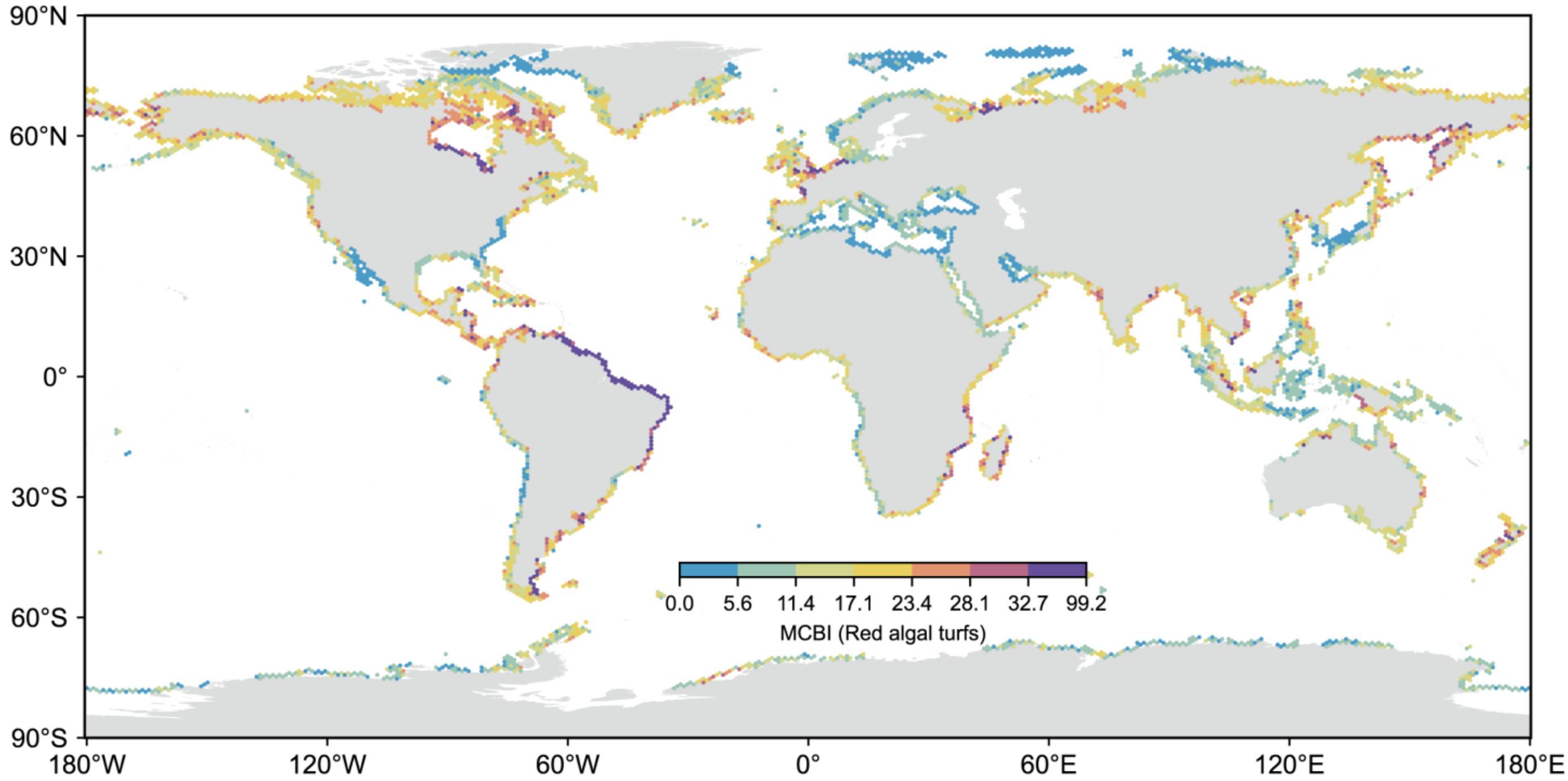

Supplementary Fig. 18 | Global distribution of MCBI for red algal turfs. Basemap data are from Natural Earth.

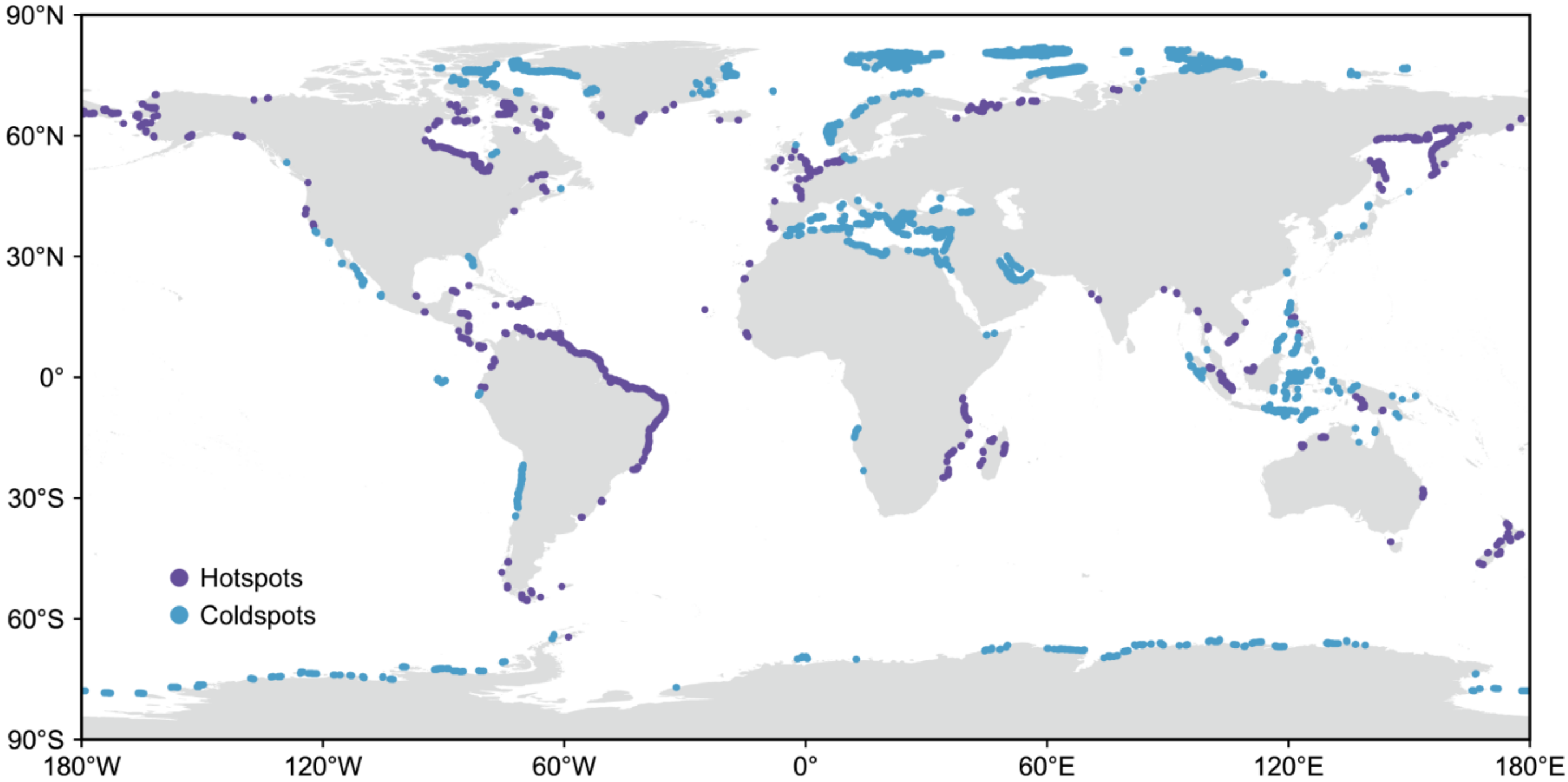

Supplementary Fig. 19 | Hotspots and coldspots of MCBI for red algal turfs, identified as co-hotspots and co-coldspots of red-algal NPP and SAR (Getis-Ord Gi*; 95% confidence level). Basemap data are from Natural Earth.

Supplementary Results 4: Relationship between coastline fractal dimension and MCBI

To quantify the geomorphological control on burial favorability, we examined the relationship between coastline fractal dimension (D) and mean MCBI within each grid cell. Although the theoretical fractal dimension of a coastline lies between $1 \leq D < 2$, initial calculations yielded values below 1 in some grid cells. This artifact arises from the rasterization resolution (2,200 m), which does not fully resolve fine-scale features (e.g., small islets) in cells containing minimal landmass. Grid cells with theoretically invalid dimensions ($D < 1$) were therefore excluded, leaving 202 valid grid cells for analysis (Supplementary Table 6).

To mitigate inherent spatial noise and extract the underlying trend, we applied an equal-interval binning strategy prior to model fitting. The observed fractal dimension range was segmented into 10 bins with a width of 0.06. We then calculated the mean D and mean MCBI for each bin. Linear regression performed on these bin-averaged values revealed a robust positive correlation (R^2=0.59, p=0.0096), confirming that geometric complexity serves as a strong predictor of carbon burial favorability.

Supplementary Table 6 | The geographic extent of each coastline grid, its fractal dimensions, and the mean MCBI.

ID	Fractal dimension	Average MCBI	Longitude(min)	Longitude(max)	Latitude(min)	Latitude(max)
1	1.126093	0.307046	-150	-140	-80	-70
2	1.116135	0.748488	-130	-120	-80	-70
3	1.097028	0.918912	-120	-110	-80	-70
4	1.009343	0.959886	-110	-100	-80	-70
5	1.068142	0.540709	-100	-90	-80	-70
6	1.020715	0.590305	-90	-80	-80	-70
7	1.001165	1.174511	-80	-70	-80	-70
8	1.035922	1.147496	-10	0	-80	-70
9	1.033085	1.254096	0	10	-80	-70
10	1.095838	1.04426	160	170	-80	-70
11	1.318866	2.344465	-70	-60	-70	-60
12	1.265798	2.835604	-60	-50	-70	-60
13	1.103173	0.853119	40	50	-70	-60
14	1.084327	1.089706	50	60	-70	-60
15	1.007824	0.740813	60	70	-70	-60
16	1.037512	0.871832	90	100	-70	-60
17	1.006607	0.738907	100	110	-70	-60
18	1.015009	0.601041	110	120	-70	-60
19	1.028165	0.756518	120	130	-70	-60
20	1.099819	1.163627	140	150	-70	-60
21	1.584522	11.2982	-80	-70	-60	-50
22	1.316583	21.70879	-70	-60	-60	-50
23	1.514176	14.2563	-60	-50	-60	-50
24	1.210925	2.393207	-40	-30	-60	-50
25	1.5483	12.43869	-80	-70	-50	-40
26	1.100781	19.90837	-70	-60	-50	-40
27	1.452246	9.335581	60	70	-50	-40
28	1.29811	9.043822	140	150	-50	-40
29	1.127216	12.70692	160	170	-50	-40
30	1.101695	11.65318	170	180	-50	-40
31	1.133424	14.60524	-70	-60	-40	-30
32	1.038498	7.716322	-60	-50	-40	-30
33	1.01295	7.432269	20	30	-40	-30
34	1.097411	1.941118	110	120	-40	-30
35	1.161203	4.406685	130	140	-40	-30
36	1.029976	5.882274	140	150	-40	-30
37	1.235498	10.44979	170	180	-40	-30
38	1.075859	9.337637	-50	-40	-30	-20
39	1.007567	2.878154	10	20	-30	-20
40	1.086139	2.816022	110	120	-30	-20
41	1.162327	9.117259	140	150	-30	-20
42	1.056535	8.700882	150	160	-30	-20
43	1.026953	6.660395	160	170	-30	-20
44	1.046278	13.50094	-40	-30	-20	-10

45	1.103163	8.341455	40	50	-20	-10
46	1.137691	6.314568	120	130	-20	-10
47	1.284428	3.426145	130	140	-20	-10
48	1.017836	6.351802	140	150	-20	-10
49	1.130746	8.679629	170	180	-20	-10
50	1.246216	7.100088	-100	-90	-10	0
51	1.021765	2.253304	-90	-80	-10	0
52	1.311776	33.62628	-60	-50	-10	0
53	1.299756	37.66935	-50	-40	-10	0
54	1.017338	3.444619	10	20	-10	0
55	1.226909	10.05954	100	110	-10	0
56	1.290238	4.457654	110	120	-10	0
57	1.388479	2.978512	120	130	-10	0
58	1.335756	4.831133	130	140	-10	0
59	1.190524	4.754581	140	150	-10	0
60	1.307677	2.046534	150	160	-10	0
61	1.105429	13.03907	-90	-80	0	10
62	1.20199	6.912473	-80	-70	0	10
63	1.184017	26.98934	-70	-60	0	10
64	1.145171	35.97866	-60	-50	0	10
65	1.115622	2.233169	-20	-10	0	10
66	1.121183	1.605278	0	10	0	10
67	1.13752	11.57429	70	80	0	10
68	1.0689	13.28463	80	90	0	10
69	1.211115	6.338226	90	100	0	10
70	1.240429	9.67286	100	110	0	10
71	1.192545	7.639728	110	120	0	10
72	1.327384	4.912032	120	130	0	10
73	1.000875	9.923111	-100	-90	10	20
74	1.099172	11.62869	-90	-80	10	20
75	1.117423	9.179544	-80	-70	10	20
76	1.238168	11.94275	-70	-60	10	20
77	1.028605	1.929179	-30	-20	10	20
78	1.204251	1.59951	-20	-10	10	20
79	1.199024	2.966603	40	50	10	20
80	1.000509	9.0593	70	80	10	20
81	1.020114	12.7861	80	90	10	20
82	1.208032	11.83379	90	100	10	20
83	1.162726	12.83	100	110	10	20
84	1.464716	8.581778	120	130	10	20
85	1.144338	1.126495	-160	-150	20	30
86	1.196756	3.325805	-120	-110	20	30
87	1.106478	0.679635	-110	-100	20	30
88	1.035683	11.46136	-100	-90	20	30
89	1.184178	10.19219	-90	-80	20	30
90	1.327576	7.991739	-80	-70	20	30
91	1.010046	9.108471	-20	-10	20	30

92	1.149803	2.374732	30	40	20	30
93	1.192669	2.057798	50	60	20	30
94	1.166929	6.016987	60	70	20	30
95	1.151476	16.0412	70	80	20	30
96	1.107116	15.85039	80	90	20	30
97	1.287789	12.79375	90	100	20	30
98	1.208628	18.94539	100	110	20	30
99	1.314913	19.41689	110	120	20	30
100	1.075331	7.320509	120	130	20	30
101	1.028764	10.22266	-130	-120	30	40
102	1.0993	6.417361	-120	-110	30	40
103	1.197807	19.53104	-90	-80	30	40
104	1.389305	6.543862	-80	-70	30	40
105	1.100451	5.614521	-10	0	30	40
106	1.04209	2.865758	0	10	30	40
107	1.119497	3.391886	10	20	30	40
108	1.334257	2.709295	20	30	30	40
109	1.105303	1.891763	30	40	30	40
110	1.059772	3.044047	50	60	30	40
111	1.041891	25.62199	110	120	30	40
112	1.305062	21.08463	120	130	30	40
113	1.320157	13.11544	130	140	30	40
114	1.30853	10.7041	-130	-120	40	50
115	1.099791	10.86626	-80	-70	40	50
116	1.306985	9.778387	-70	-60	40	50
117	1.314949	5.470035	-60	-50	40	50
118	1.135487	10.37714	-10	0	40	50
119	1.144897	12.10123	0	10	40	50
120	1.239177	13.39521	10	20	40	50
121	1.248858	6.347956	20	30	40	50
122	1.221343	12.10513	30	40	40	50
123	1.097941	0.05986	40	50	40	50
124	1.007269	18.12103	120	130	40	50
125	1.046964	16.73812	130	140	40	50
126	1.229493	16.27463	140	150	40	50
127	1.027516	4.75237	-180	-170	50	60
128	1.204312	5.021452	-170	-160	50	60
129	1.366503	10.01583	-160	-150	50	60
130	1.510689	11.47219	-140	-130	50	60
131	1.452243	8.024735	-130	-120	50	60
132	1.076637	10.06389	-100	-90	50	60
133	1.155873	3.625893	-80	-70	50	60
134	1.25473	9.217312	-70	-60	50	60
135	1.343642	5.717319	-60	-50	50	60
136	1.370831	13.99403	-10	0	50	60
137	1.28826	13.0396	0	10	50	60
138	1.354122	12.46462	10	20	50	60

139	1.257187	20.16027	20	30	50	60
140	1.235412	31.07726	130	140	50	60
141	1.147693	25.65436	140	150	50	60
142	1.134922	11.60113	150	160	50	60
143	1.07107	7.162124	160	170	50	60
144	1.154279	5.236505	-180	-170	60	70
145	1.151913	6.410531	-170	-160	60	70
146	1.024886	13.8268	-160	-150	60	70
147	1.245904	8.446129	-150	-140	60	70
148	1.232761	8.165483	-140	-130	60	70
149	1.049369	9.98611	-130	-120	60	70
150	1.190436	2.911635	-120	-110	60	70
151	1.364175	10.29976	-110	-100	60	70
152	1.296961	9.231183	-100	-90	60	70
153	1.247126	12.61416	-90	-80	60	70
154	1.263369	10.08946	-80	-70	60	70
155	1.36774	6.967103	-70	-60	60	70
156	1.441739	8.033661	-60	-50	60	70
157	1.322912	10.52247	-50	-40	60	70
158	1.301305	4.190111	-40	-30	60	70
159	1.248101	9.365258	-30	-20	60	70
160	1.193198	9.871454	-20	-10	60	70
161	1.393633	8.972024	-10	0	60	70
162	1.422299	7.207592	0	10	60	70
163	1.382597	9.683801	10	20	60	70
164	1.254631	17.83651	20	30	60	70
165	1.098889	17.54829	30	40	60	70
166	1.107494	18.80112	40	50	60	70
167	1.157186	15.1786	50	60	60	70
168	1.117514	18.91138	60	70	60	70
169	1.103706	23.51437	70	80	60	70
170	1.122844	3.233329	160	170	60	70
171	1.086217	5.557604	170	180	60	70
172	1.069366	9.063026	-170	-160	70	80
173	1.132057	13.80771	-160	-150	70	80
174	1.062558	4.89642	-130	-120	70	80
175	1.181793	1.740887	-120	-110	70	80
176	1.174527	1.72008	-110	-100	70	80
177	1.225593	4.587645	-100	-90	70	80
178	1.246399	1.94467	-90	-80	70	80
179	1.20099	1.427045	-80	-70	70	80
180	1.110258	0.590148	-70	-60	70	80
181	1.268461	2.327465	-60	-50	70	80
182	1.254307	2.120627	-30	-20	70	80
183	1.141675	0.6439	-20	-10	70	80
184	1.205194	3.158097	10	20	70	80
185	1.218065	4.090566	20	30	70	80

186	1.173422	14.54289	50	60	70	80
187	1.010518	4.797808	60	70	70	80
188	1.079485	21.25221	70	80	70	80
189	1.12425	15.04609	80	90	70	80
190	1.116614	3.219323	90	100	70	80
191	1.069619	3.83786	100	110	70	80
192	1.109608	1.607724	110	120	70	80
193	1.253822	1.35862	120	130	70	80
194	1.11887	1.37864	130	140	70	80
195	1.106929	1.596386	140	150	70	80
196	1.064942	0.881816	-90	-80	80	90
197	1.012529	0.399533	-70	-60	80	90
198	1.134214	3.296976	10	20	80	90
199	1.192036	0.228572	20	30	80	90
200	1.070183	0.169816	40	50	80	90
201	1.254647	0.202616	50	60	80	90
202	1.053542	0.313494	90	100	80	90

Supplementary Results 5: National-level assessment of MCBI

To enable equitable cross-jurisdictional comparison, we quantified both the total national MCBI and the MCBI per kilometre of coastline (MCBIc) for each coastal nation. All spatial aggregations were performed at 5-arcminute resolution. To avoid artifacts arising from very limited coastal extent, jurisdictions represented by fewer than five grid cells were excluded from the comparative analysis; accordingly, Slovenia (n = 3 grid cells) and Iraq (n = 4 grid cells) were omitted (Supplementary Table 7).

Supplementary Table 7 | Number of MCBI grid cells intersecting each country's (or region's) coastline, MCBI per kilometre of coastline (MCBIc), and total MCBI, for each coastal nation or region.

Country/Region ISO code	MCBI count	MCBIc	Total MCBI
AGO	207	0.346888799	689.4692383
ALA	26	1.129657561	667.0966797
ALB	48	1.176569931	545.6578369
ARE	106	0.103567952	127.248764
ARG	707	2.178362241	13525.16797
ATF	78	0.454408252	757.666687
ATG	8	0.371382624	67.50993347
AUS	2937	0.446085078	16427.9707
BEL	13	3.519183958	326.2987366
BEN	15	0.220291807	24.26734543
BGD	89	0.366355177	1225.311523
BGR	39	0.33172585	112.7867889
BHR	14	0.063080337	15.93598557
BHS	115	0.188911337	721.803772
BLZ	44	0.358681798	306.3106689
BRA	1191	1.363456859	25718.17773
BRB	7	0.910944868	83.3059082
BRN	22	1.083640644	185.1833496
CAN	12902	0.512977233	91092.77344
CHL	1897	0.631468767	21604.9082
CHN	1200	1.236095771	23614.25
CIV	65	0.103119228	125.2393341
CMR	61	0.126830786	102.3334198
COD	8	0.046523418	9.488916397
COG	24	0.150579133	26.85729408
COL	278	0.59512403	2152.807617
CPV	87	0.197485048	167.8385925
CRI	139	1.300858283	1864.168945
CUB	432	0.577658534	3263.955566
CYP	64	0.054819937	29.6055069
DEU	187	0.829798334	2240.986572
DJI	39	0.317112634	100.6959457
DMA	14	1.085726771	143.6959381
DNK	379	0.940200665	4134.259766
DOM	128	1.027737463	1306.449585
DZA	176	0.341082717	406.1681213
ECU	255	0.411773828	1084.591675
EGY	386	0.275044073	1067.140747
ERI	152	0.329105181	486.3812561
ESH	129	1.682755393	1814.178589
ESP	580	0.816925334	4102.835938
EST	168	2.273435157	3534.987061
FIN	142	0.970696894	3560.12793

FJI	104	0.331289622	902.6813965
FLK	141	0.852920326	2195.775146
FRA	553	1.298869911	8192.972656
FRO	27	0.347361754	252.0873718
FSM	8	0.417864895	143.9335632
GAB	114	0.088835859	172.4837036
GBR	1020	1.369221626	16400.85156
GEO	45	0.012511092	4.081743717
GHA	72	0.247640082	138.2005005
GIN	48	0.139297313	125.3829041
GMB	18	0.046438693	22.63375473
GNB	69	0.081467057	157.5752106
GNQ	51	0.1739238	87.875
GRC	684	0.213410545	2042.328247
GRD	8	0.807397621	96.33868408
GRL	4022	0.334538857	17273.05078
GTM	49	1.515657623	554.6549072
GUM	8	0.620055121	79.60267639
GUY	68	2.043299189	1697.797729
HMD	12	0.306586092	34.2456665
HND	96	0.826424692	1194.175415
HRV	124	0.438876135	1233.610596
HTI	154	1.071322765	1503.397949
IDN	4069	0.424981189	22367.16797
IMN	16	1.954310619	233.911438
IND	853	0.910008812	10256.09961
IRL	306	0.845498603	2994.544678
IRN	283	0.200663284	692.5652466
ISL	576	1.144221082	6262.99707
ISR	26	0.198572898	39.68280792
ITA	727	1.127202315	6849.963379
JAM	72	1.644070518	1005.168274
JPN	1510	1.043284512	18831.52539
KEN	67	0.581446528	492.1072693
KHM	46	0.337686824	278.976593
KOR	249	0.751160099	4117.671875
KWT	30	0.730535135	364.1644592
LBN	29	0.070539575	15.69576073
LBR	77	0.290417689	170.6378174
LBY	258	0.292499238	519.902771
LCA	10	1.046560671	119.2346573
LKA	150	0.92469611	1886.509521
LTU	14	1.358392941	269.3829041
LVA	83	2.633990302	1345.310547
MAR	253	0.93795302	1675.765625
MDG	513	0.529846074	3028.070313
MEX	1415	0.510999939	7983.055664

MMR	338	0.602941742	4009.29126
MNE	17	1.540406916	266.7676697
MOZ	351	1.025388233	4081.773193
MRT	89	0.215885935	230.2574615
MUS	12	0.08986071	23.13463974
MYS	509	0.73287016	3912.258789
NAM	201	0.55980693	874.1889038
NCL	146	0.520106759	972.4176025
NGA	112	0.076901131	167.0246429
NIC	127	1.18299238	1668.516113
NIU	8	0.058197798	3.768889427
NLD	112	1.387068671	1608.2229
NOR	2894	0.542712998	15737.09766
NZL	829	0.704670742	9333.666992
OMN	279	0.414041542	949.4138184
PAK	124	0.34440609	499.9639893
PAN	243	0.894289293	3006.698975
PER	339	0.749395789	2091.556152
PHL	1572	0.719197093	12949.6543
PNG	920	0.275685284	3044.684814
POL	88	1.638442526	1145.812012
PRI	52	0.799923684	509.8313599
PRK	178	1.563799839	3745.206787
PRT	186	0.553442282	1169.838623
PSE	6	0.398066059	15.03097439
PYF	30	0.014669405	34.18983459
QAT	50	0.093528205	52.76674271
ROU	37	2.060444551	944.6314087
RUS	12247	1.232943367	127161.8828
SAU	322	0.221596921	933.9423828
SDN	71	0.199824065	154.9176025
SEN	60	0.041167088	52.50862122
SGP	5	0.612483508	57.02833939
SGS	52	0.126810494	124.4467468
SLB	318	0.195504013	866.6282349
SLE	66	0.145913653	139.5124207
SLV	37	1.14686135	489.0446167
SOM	376	0.234787944	749.3726807
STP	14	0.094888454	18.00793076
SUR	52	3.010374472	1770.852783
SWE	474	0.862004139	5725.224609
SYR	20	0.033096276	6.024184227
TCA	5	0.132854893	49.99993896
TGO	8	0.302550066	13.86586952
THA	310	0.585791291	2367.141602
TLS	73	0.06825342	47.98966217
TTO	44	1.998339622	1019.293091

TUN	162	0.397895408	550.961792
TUR	657	0.244022872	1428.009644
TZA	160	0.812321679	1603.81543
UKR	316	1.309886693	3800.033691
URY	86	0.331763592	291.9619141
USA	5340	0.577102894	50950.66016
VCT	8	0.567538229	79.35319519
VEN	359	0.678116534	3668.440918
VNM	350	0.852817347	5144.765625
WSM	33	0.603413703	211.4723663
YEM	263	0.279903117	696.9055786
ZAF	411	0.89224469	2802.504883

Supplementary Results 6: National assessment of conservation coverage

At the national scale, we computed the MCBI-weighted protection rate for each of the 149 coastal jurisdictions included in our analysis. Consistent with the criteria applied in Supplementary Results 5, jurisdictions represented by fewer than five grid cells (e.g., Slovenia and Iraq) were excluded. These national-level results (Supplementary Table 8) reveal a pronounced disparity in conservation coverage: a minority of jurisdictions exceed the 33% weighted-coverage reference level, whereas a distinct cluster of nations with high total MCBI remains well below both the global weighted average and this reference level.

Supplementary Table 8 | The total MCBI for each country (or region), total protected MCBI for each country (or region), and the protection rate.

Country/Region ISO code	Total MCBI	Total protected MCBI	Protection rate (%)
AGO	689.4692383	28.2188746	4.092840266
ALA	667.0966797	50.6138382	7.587181849
ALB	545.6578369	16.9978044	3.115103139
ARE	127.248764	12.48019199	9.807711757
ARG	13525.16797	3814.372552	28.20203461
ATF	757.666687	757.6666532	99.99999553
ATG	67.50993347	23.23776293	34.42124993
AUS	16427.9707	4750.687044	28.91828291
BEL	326.2987366	207.617095	63.62791875
BEN	24.26734543	0	0
BGD	1225.311523	192.6385803	15.72160031
BGR	112.7867889	53.17174077	47.14358948
BHR	15.93598557	0	0
BHS	721.803772	258.6439938	35.83300668
BLZ	306.3106689	37.4486649	12.22571353
BRA	25718.17773	10475.73131	40.73278992
BRB	83.3059082	0	0
BRN	185.1833496	15.94297409	8.609291345
CAN	91092.77344	7153.698493	7.853200889
CHL	21604.9082	9404.928667	43.5314447
CHN	23614.25	1052.110302	4.455404265
CIV	125.2393341	0	0
CMR	102.3334198	50.8064056	49.64791141
COD	9.488916397	2.447686851	25.7952199
COG	26.85729408	11.20819986	41.73242406
COL	2152.807617	572.9540063	26.61426881
CPV	167.8385925	0	0
CRI	1864.168945	324.2755933	17.39518267
CUB	3263.955566	952.6792025	29.18787291
CYP	29.6055069	2.032929835	6.866728687
DEU	2240.986572	1139.067125	50.82882419
DJI	100.6959457	26.55476356	26.37123408
DMA	143.6959381	2.614249229	1.819292364
DNK	4134.259766	1037.59143	25.09739322
DOM	1306.449585	499.9625846	38.26880044
DZA	406.1681213	1.414306402	0.348207141
ECU	1084.591675	814.4194426	75.08995888
EGY	1067.140747	256.3010672	24.01755044
ERI	486.3812561	169.0530305	34.75730784
ESH	1814.178589	0	0
ESP	4102.835938	1264.911116	30.83016565
EST	3534.987061	1373.656886	38.85889432
FIN	3560.12793	356.9558792	10.02649023

FJI	902.6813965	271.994246	30.1318103
FLK	2195.775146	0	0
FRA	8192.972656	3656.296401	44.62722572
FRO	252.0873718	0	0
FSM	143.9335632	0	0
GAB	172.4837036	63.2170077	36.65100318
GBR	16400.85156	6222.86401	37.94232261
GEO	4.081743717	0.485196859	11.88699958
GHA	138.2005005	0	0
GIN	125.3829041	27.31301129	21.78368055
GMB	22.63375473	1.157215238	5.112785092
GNB	157.5752106	44.26978469	28.09438396
GNQ	87.875	3.618692994	4.118000562
GRC	2042.328247	481.8581927	23.59357235
GRD	96.33868408	29.46308041	30.58281384
GRL	17273.05078	1418.951489	8.214828447
GTM	554.6549072	53.73625743	9.688232581
GUM	79.60267639	0	0
GUY	1697.797729	132.0600705	7.778315887
HMD	34.2456665	34.24566478	99.99999495
HND	1194.175415	345.3663063	28.9209024
HRV	1233.610596	203.9625981	16.53379104
HTI	1503.397949	294.177624	19.56751532
IDN	22367.16797	1165.901802	5.212558887
IMN	233.911438	58.57417679	25.04117682
IND	10256.09961	16.89295197	0.16471127
IRL	2994.544678	541.8447433	18.09439503
IRN	692.5652466	25.16553678	3.633670172
ISL	6262.99707	407.4111738	6.505051324
ISR	39.68280792	0	0
ITA	6849.963379	1478.328346	21.58155109
JAM	1005.168274	215.8897271	21.47796869
JPN	18831.52539	7525.37416	39.96157509
KEN	492.1072693	62.92635584	12.78712179
KHM	278.976593	76.8449465	27.54530252
KOR	4117.671875	683.4441543	16.59782943
KWT	364.1644592	0	0
LBN	15.69576073	1.613092661	10.27725058
LBR	170.6378174	15.51437426	9.091990565
LBY	519.902771	0	0
LCA	119.2346573	19.72300816	16.5413384
LKA	1886.509521	21.94424248	1.16321928
LTU	269.3829041	175.3412457	65.08996785
LVA	1345.310547	511.2212756	38.0002429
MAR	1675.765625	215.5980424	12.86564417
MDG	3028.070313	629.4546111	20.7873182
MEX	7983.055664	2142.41298	26.83700416

MMR	4009.29126	43.24714798	1.078673141
MNE	266.7676697	0	0
MOZ	4081.773193	589.1845876	14.43452538
MRT	230.2574615	66.46951652	28.86747559
MUS	23.13463974	0	0
MYS	3912.258789	238.3826092	6.093221896
NAM	874.1889038	151.3244906	17.31027355
NCL	972.4176025	223.9073181	23.02583967
NGA	167.0246429	0	0
NIC	1668.516113	425.1361824	25.47989672
NIU	3.768889427	0	0
NLD	1608.2229	832.4457474	51.76183893
NOR	15737.09766	1442.583284	9.16676833
NZL	9333.666992	161.3940872	1.729160547
OMN	949.4138184	521.0410755	54.88029197
PAK	499.9639893	10.29416037	2.058980364
PAN	3006.698975	589.9371647	19.62075917
PER	2091.556152	154.0846588	7.366986472
PHL	12949.6543	2125.146366	16.41083474
PNG	3044.684814	84.56204122	2.777366012
POL	1145.812012	652.4835851	56.94508161
PRI	509.8313599	45.55677128	8.935654976
PRK	3745.206787	0	0
PRT	1169.838623	490.5975667	41.93720031
PSE	15.03097439	0	0
PYF	34.18983459	0	0
QAT	52.76674271	7.601760209	14.40634729
ROU	944.6314087	791.59097	83.79892546
RUS	127161.8828	8560.755319	6.732170938
SAU	933.9423828	216.0022494	23.1280059
SDN	154.9176025	12.1538229	7.845346623
SEN	52.50862122	16.49221736	31.40858964
SGP	57.02833939	0	0
SGS	124.4467468	54.53167987	43.81928918
SLB	866.6282349	5.874927819	0.677906348
SLE	139.5124207	52.05486846	37.31199575
SLV	489.0446167	130.943985	26.77546803
SOM	749.3726807	0	0
STP	18.00793076	0	0
SUR	1770.852783	1402.843914	79.21855093
SWE	5725.224609	844.488728	14.75031611
SYR	6.024184227	0	0
TCA	49.99993896	35.1774292	70.35494428
TGO	13.86586952	0	0
THA	2367.141602	501.7260737	21.19543982
TLS	47.98966217	2.278989516	4.748917607
TTO	1019.293091	0	0

TUN	550.961792	43.91015053	7.969726969
TUR	1428.009644	44.92705163	3.146130829
TZA	1603.81543	435.5872138	27.15943529
UKR	3800.033691	1274.930637	33.55050876
URY	291.9619141	105.4042068	36.10203994
USA	50950.66016	7503.925646	14.72782811
VCT	79.35319519	10.56211185	13.31025402
VEN	3668.440918	349.5987444	9.529899819
VNM	5144.765625	800.0076758	15.54993432
WSM	211.4723663	0	0
YEM	696.9055786	27.5102379	3.947484243
ZAF	2802.504883	854.1576109	30.47836299

References

1. Lyard, F., Allain, D., Cancet, M. & Picot, N. FES2014 global ocean tide atlas: design and performance. *OCEAN. SCI.* **17,** 615–649 (2021).
2. Pickering, M. D. et al. The impact of future sea-level rise on the global tides. *Cont. Shelf Res.* **142**, 50–68 (2017).
3. Schuerch, M. et al. Future response of global coastal wetlands to sea-level rise. *Nature* **561**, 231–234 (2018).
4. Holgate, S. J. et al. New Data Systems and Products at the Permanent Service for Mean Sea Level. *J. Coast. Res.* **29**, 493–504 (2012).
5. Tyberghein, L. et al. Bio-ORACLE: a global environmental dataset for marine species distribution modelling. *Global Ecol. Biogeogr.* **21**, 272–281 (2012).
6. Assis, J. et al. Bio-ORACLE v2.0: Extending marine data layers for bioclimatic modelling. *Global Ecol. Biogeogr.* **27**, 277–284 (2018).
7. Restreppo, G. A., Wood, W. T. & Phrampus, B. J. Oceanic sediment accumulation rates predicted via machine learning algorithm: towards sediment characterization on a global scale. *Geo-Mar. Lett.* **40**, 755–763 (2020).
8. Meyer, H. & Pebesma, E. Machine learning-based global maps of ecological variables and the challenge of assessing them. *Nat. Commun.* **13**, 2208 (2022).
9. Pessarrodona, A. et al. Global seaweed productivity. *Sci. Adv.* **8**, eabn2465 (2022).
10. Miller, R. J., Reed, D. C. & Brzezinski, M. A. Partitioning of primary production among giant kelp (*Macrocystis pyrifera*), understory macroalgae, and phytoplankton on a temperate reef. *Limnol. Oceanogr.* **56**, 119–132 (2011).
11. Bradley, J.A., Hülse, D., LaRowe, D.E. & Arndt, S. Transfer efficiency of organic carbon in marine sediments. *Nat. Commun.* **13**, 7297 (2022).
12. Filbee-Dexter, K. et al. Kelp carbon sink potential decreases with warming due to accelerating decomposition. *PLOS Biol.* **20**, e3001702 (2022).
13. Davidson, E. A. & Janssens, I. A. Temperature sensitivity of soil carbon decomposition and feedbacks to climate change. *Nature* **440**, 165–173 (2006).
14. Arndt, S. et al. Quantifying the degradation of organic matter in marine sediments: A review and synthesis. Earth-Sci. Rev. **123**, 53–86 (2013).
15. Dinerstein, E. et al. A Global Deal For Nature: Guiding principles, milestones, and targets. *Sci. Adv.* **5**, eaaw2869 (2019).
16. Wang, F., Lu, X., Sanders, C. J. & Tang, J. Tidal wetland resilience to sea level rise increases their carbon sequestration capacity in United States. *Nat. Commun.* **10**, 5434 (2019).
