# Supplementary material for: The global distribution of macroalgal carbon burial in coastal sediments
Source: Nat Commun. 2026 Aug 31;17:10372. doi: 10.1038/s41467-026-77215-7 (PMC13627689; doi:10.1038/s41467-026-77215-7)
Supplement: Supplementary file 2 [file 41467_2026_77215_MOESM2_ESM.pdf]

nature portfolio

Peer Review File

The Global Distribution of Macroalgal Carbon Burial in Coastal Sediments

Corresponding Author: Professor Xi Xiao

This file contains all reviewer reports in order by version, followed by all author rebuttals in order by version.

Version 0:

Reviewer comments:

Reviewer #1

(Remarks to the Author)
This study employs a machine learning model to map the global coastal sedimentation accumulation rate (SAR) distribution, then couples it with net primary productivity (NPP) to estimate the global Macroalgal Carbon Burial Potential (MCBP) in coastal sediments, and finally provides constructive suggestions for marine protected area establishment and blue carbon management. The research demonstrates a novel perspective, a clear objective, and substantial effort, which is highly commendable. The introduction is well-written, and the results and discussion sections are generally solid and clearly presented. My review comments primarily focus on the methodology section, and several key methodological aspects may require further clarification and refinement.

Main Comments
1. Source and Temporal Resolution of SAR
I think that the manuscript does not provide sufficient metadata description for the SAR dataset: it does not specify the measurement methods or the original temporal resolution of the SAR data (e.g., whether it reflects decadal or annual averages), mentioning only the publication years of the source literature. This raises two concerns: First, does the calculated MCBP represent a contemporary potential or reflect an average state over decades? Second, if the SAR data from different regions correspond to inconsistent temporal scales, are the resulting MCBP values comparable?

2. Simulation of SAR
Line 374 mentions considering both hydrodynamics and sediment supply as drivers of SAR, yet the predictors listed in Lines 360-363 include only hydrodynamic drivers and do not incorporate sediment supply.

3. Calculation Methodology of MCPB
When reading the article, it appears that MCPB is primarily formulated as a coupling of SAR and NPP (Formula 3), which reduces the complex fate of organic carbon to two variables. However, certain regions with high SAR (e.g., high-energy mobile muds) may exhibit very low organic matter burial efficiency (Aller and Blair, 2006). The presentation of SAR as a proxy for “geological retention capacity” in Line 150 may lead to misinterpretation, since SAR is a measure of burial rate and may not be the preservation efficiency. Organic carbon also undergoes significant influences from other biological processes before final burial, beyond sedimentation rate alone. To further increase the confidence in these findings, I wonder if authors can validate their calculations against available in-situ data (e.g., from published studies) or discuss the potential uncertainties.

4. Equal-interval binning strategy
Figure 3c employs an equal-interval binning strategy. What is the rationale for adopting this grouping approach? The gray scatter points in the figure suggest no apparent correlation between shoreline complexity and MCBP. Would the results differ if different bin widths or grouping intervals were chosen?

Other minor comments
The content in Lines 108-112 of the Results section has already been described in the Methods section.
Line 93, “Results” or “Results and Discussion”?
Line 389: I am unclear about the meaning of “weighted equally” here. Does multiplying the two factors imply equal weighting in the subsequent formula?

I hope the authors find this review informative and helpful.

(Remarks on code availability)

Reviewer #2

(Remarks to the Author)
This study assesses the storage（burial）potential of organic carbon originated from macroalgal primary production in coastal areas around the globe at high resolution (5-arcmin) by model simulations. The results also provide some recommendations for national blue carbon management measures in coastal areas around the world. The study presents important concepts for establishing assessment methods to identify areas where macroalgal carbon storage is most effective for climate change mitigation, such as the creation and restoration of seaweed beds and the promotion of seaweed farming. However, I think that careful and humble attitudes are necessary when discussing the methodologies and results of this study.

This study is extremely challenging, but the results also have significant uncertainty. The calculation of MCBP in this study uses two parameters: a global-level estimated NPP from a previous study (Pessarrodona et al. 2021) and an original estimated SAR by the authors. However, both parameters are estimates and have significant uncertainties. Actually, I reside in a country rated as having a high MCBP in this study, but the results displayed in the figure do not necessarily match the results of our related research results and our own experiences. The manuscript also notes on the contradiction between that the results of this study suggesting that high-latitude regions are MCDP cold spots and previous literatures in which fjord areas are suggested to serve as a reservoir of macroalgal organic carbon (L159-163). Authors mention that this discrepancy is attributed to the resolution of this method, however, if there is any evidence in previous studies that could support or strengthen the results, please provide it as much as possible.

Furthermore, the fate of macroalgal primary production in coastal areas involves biological processes such as decomposition and being consumed by herbivorous animals. These processes are likely to be significantly affected by factors such as water temperature and local fauna, which varies greatly depending on latitude. Therefore, these factors are considered to affect the amount of macroalgal organic matter being deposited on the seafloor of coastal areas, and thus the MCDP. Although I acknowledge that it is currently very difficult to incorporate these factors into the model, discussing burial potential solely in terms of NPP and SAR is too simplistic and is considered to be a major source of uncertainty in the results obtained. Threfore, I think these parameters (decomposition and consumption) should be incorporated into future updates of this method. In order to improve the accuracy of the results of this study, I think there should be further discussion about what improvements and information are needed in the future.

As mentioned above, there is still a great deal of uncertainty in the results. So I recommend that assessing MCDP on a country-by-country basis as shown in Figure 4 should be reconsidered its suitability. Also, Figure 4 shows income levels, but is there any point in showing them in this study? At best, I think the results should be expressed in larger regional groups, such as East Asia or the Atlantic coast of North America (as examples), and discussion of country-by-country assessments should be scaled down.

(Remarks on code availability)

Reviewer #3

(Remarks to the Author)
Significance

This work, is from what I am aware, novel in its approach and conclusions and would be a great addition to the literature and community. The approach is interesting and provides a potentially valuable framework for identifying critical areas of potential macroalgal C burial. However, careful consideration should be made to the underlying assumptions made in conducting the modeling here as there seems to be sparse discussion/inclusion of error/uncertainties in their conclusions in the body of the text itself - I think it’s important to disclose and discuss as we move towards a better understanding of algal standing stocks, NPP, and C cycling/sequestration as a field - transparency is key.

Key results

This work presents an investigation of macroalgal carbon burial potential modeled globally, outlining the importance of macroalgae as a potential nature-based climate mitigation solution and the results draw attention to regions/nations where potential burial hot spots do not line up with marine protected areas - indicating room for improvement and suggesting this research might serve as a potential framework to make said improvements.

- Two main outcomes/products of the work are 1) a proxy built for identifying high potential macroalgal C burial areas using coastal complexity (fractal dimension) and 2) the identification of the mismatch between high potential C burial regions and

MPAs and suggestion for improved alignment.

Validity

The study seems to provide a robust analysis of the data collected and used for their models.

One very important point, which is made only in the methodology section of the paper is the data used especially for the macroalgal NPP underpinning the entire analysis. The authors are using a modeled product or modeled estimates of macroalgal NPP to determine their MCBP metric, which should - in my opinion - be outlined very clearly in the paper as these estimates come with high levels of uncertainty.

In addition, the definition of macroalgal NPP and carbon in general for that matter are not very well defined in the body of the text. How are the authors defining macroalgal NPP? Macroalgal NPP might very well include dissolved organic C production, does it in this case? If it does, how does that contribute to coastal carbon burial, when we know DOC will either be remineralized or be sequestered to the deep oceans? These distinctions and definitions should be more explicitly outlined in the main body of the text.

Data and methodology

The handling of data in general is appropriate, and from limited knowledge of the particular model used in the study, the rationale and execution seem well founded.

Suggested improvements

Improved transparency on the macroalgal NPP data underpinning the results should be carefully considered, especially as the authors are suggesting this work be used by policy-makers for designations of MPAs in the future that better encapsulate a co-benefit of MPA designation - macroalgal C burial/sequestration.

References

The references seem appropriate.

Line-by-Line:

Line 28: 5 arcmin is fine, however, might be misleading to the reader as higher res than it really is (10km) - maybe should be expressed in km for this reason?

Line 49: with respect to discussion on refractory DOC - yes and DOC is released throughout growth so would/should be considered in the calculation of any C sink —- where does DOC come into play in your macroalgal NPP dataset? Is it considered or excluded from these numbers? This should be made clear.

Line 66: not only does detrital C serve as a pool of C with sequestration potential, but the algal biomass itself is stochastically 'sunk' or transported as well.. unless you are looping this into your definition of detrital material - which in this case should be clearly defined

Line 70: what happens in low SAR ... could speak briefly on biodegradation of the POC in these settings and the prevention of sequestration?

Line 73: is there any ground-truthed data/areas in the literature that show this connection/process? if so could cite it here to solidify point

Line 114: since this is a big factor you should mention it earlier in the study and drive home that your modeling is accounting for it

Line 126: is this not well known that riverine estuaries are high depositional regions? your findings support that knowledge... should cite pioneering work on this

Line 142: do you have map of seaweed NPP based on your collection of modeled estimates? could include it in the SI

Line 179: should this read "… potential coastal carbon burial…" instead? personal preference

Line 212: can you include the sample number (n = ?) for this correlation

Line 220: are you just talking about POC (defined how?) in this study or also entire algal biomass? where is this defined if at all?

Line 271: consequences of all this extra seaweed burial from aquaculture? could include one line on this. If harvested... can we count DOC and POC during growth towards C burial?

Line 280: expand on this. it's important and a large barrier/constraint to progress

Line 382: this is a really important point that underlies all of your findings. The use of modeled algal NPP has large uncertainties ----- how do they bare out in your analysis... nothing has error bars

(Remarks on code availability)
I am an R user, so review of the python code was outside my expertise.

Version 1:

Reviewer comments:

Reviewer #1

(Remarks to the Author)
This version of the manuscript is greatly improved over the first version I looked at. It does a much better job of explaining what was done and resolving the issues I raised.

(Remarks on code availability)

Reviewer #4

(Remarks to the Author)
Please accept my apologies for a delayed review. See document attached

(Remarks on code availability)
I did have a glance at the data, looks like a good chunk of it comes from existing compilations but I do have some concerns about its quality control and whether they can be used in your model (see my comment)

Version 2:

Reviewer comments:

Reviewer #4

(Remarks to the Author)
I recognize this has been a long process for the authors and welcome their openness to take constructive feedback. I do have an outstanding concern that will leave the editor to decide on.

I don’t really agree that Bradley et al. parametrize transfer efficiency “largely based on SAR and water depth”. There is quite an effort to characterize post-depositional organic matter degradation dynamics in their metric (a key weakness of the MCBI), and I personally don’t buy the redundancy argument made by the authors in this revision. Incorporating transfer efficiency (e.g., Fig. 3a; the 100-year burial efficiency appears particularly relevant) into the MCBI metric would make it a much stronger and more valuable indicator - and that is coming from someone that is not even on that paper! Burial efficiency is already normalized on a 0–100 scale, and so incorporating it should not introduce any substantial numerical complications.

(Remarks on code availability)
Thanks for making the data accessible

Version 3:

Reviewer comments:

Reviewer #4

(Remarks to the Author)
The incorporation of the metric made the manuscript much stronger. Looking forward to seeing this published

(Remarks on code availability)

Open Access This Peer Review File is licensed under a Creative Commons Attribution 4.0 International License, which permits use, sharing, adaptation, distribution and reproduction in any medium or format, as long as you give appropriate credit to the original author(s) and the source, provide a link to the Creative Commons license, and indicate if changes were made.
In cases where reviewers are anonymous, credit should be given to 'Anonymous Referee' and the source.
The images or other third party material in this Peer Review File are included in the article's Creative Commons license, unless indicated otherwise in a credit line to the material. If material is not included in the article's Creative Commons license and your intended use is not permitted by statutory regulation or exceeds the permitted use, you will need to obtain permission directly from the copyright holder.
To view a copy of this license, visit https://creativecommons.org/licenses/by/4.0/

REVIEWER COMMENTS

Responses to comments from Reviewer #1:

Comment 1: This study employs a machine learning model to map the global coastal sedimentation accumulation rate (SAR) distribution, then couples it with net primary productivity (NPP) to estimate the global Macroalgal Carbon Burial Potential (MCBP) in coastal sediments, and finally provides constructive suggestions for marine protected area establishment and blue carbon management. The research demonstrates a novel perspective, a clear objective, and substantial effort, which is highly commendable. The introduction is well-written, and the results and discussion sections are generally solid and clearly presented. My review comments primarily focus on the methodology section, and several key methodological aspects may require further clarification and refinement.

Response: Thank you very much for your positive comments. In response to your specific suggestion, we have carefully revised of the manuscript, particularly the methodology section.

Comment 2: Source and Temporal Resolution of SAR: I think that the manuscript does not provide sufficient metadata description for the SAR dataset: it does not specify the measurement methods or the original temporal resolution of the SAR data (e.g., whether it reflects decadal or annual averages), mentioning only the publication years of the source literature. This raises two concerns: First, does the calculated MCBP represent a contemporary potential or reflect an average state over decades? Second, if the SAR data from different regions correspond to inconsistent temporal scales, are the resulting MCBP values comparable?

Response: Thank you. We have provided a comprehensive dataset (available via the Figshare repository; see Data Availability) that explicitly details the geographical coordinates, specific measurement methods, the period encompassed by SAR estimates, the corresponding ecosystems, and source literature for all compiled SAR records (Supplementary Table. 1).

Regarding your two concerns: The vast majority of our compiled records were quantified using standardized radioisotope geochronology methods (e.g., ^{210}Pb, ^{137}Cs, ^{14}C), which inherently capture average accumulation rates over decadal to centennial timescales. The period encompassed by these standardized methods integrates depositional anomalies that enhances comparisons of SAR data across different regions.

We have added a detailed metadata in the Methods section (Lines 372-377):

Conventional radioisotope methods (specifically ^{210}Pb, ^{137}Cs and ^{14}C) constitute a significant proportion (64.0%) of the SAR measurement techniques in our database (see Data availability). Furthermore, a portion of the SAR data for which specific methods were not provided in the source literature were primarily derived as byproducts of long-term organic carbon burial estimations. Consequently, these SAR records predominantly characterize long-term average depositional states spanning decadal to centennial timescales.

Comment 3: Simulation of SAR: Line 374 mentions considering both hydrodynamics and sediment supply as drivers of SAR, yet the predictors listed in Lines 360-363 include only hydrodynamic drivers and do not incorporate sediment supply.

Response: We have revised the classification of the predictors into four distinct forcing mechanisms instead of three (Lines 390-394):

(i) Climate change parameters, specifically relative sea-level rise rates and tropical cyclone effects;
(ii) Sediment supply factor, including total suspended matter;
(iii) Biological factor, specifically primary productivity;
(iv) Physical oceanographic controls, encompassing water depth, mean tidal range, mean high water springs/neaps, and current velocities/directions (surface and seafloor).

Comment 4: Calculation Methodology of MCPB: When reading the article, it appears that MCPB is primarily formulated as a coupling of SAR and NPP (Formula 3), which reduces the complex fate of organic carbon to two variables. However, certain regions with high SAR (e.g., high-energy mobile muds) may exhibit very low organic matter burial efficiency (Aller and Blair, 2006). The presentation of SAR as a proxy for "geological retention capacity" in Line 150 may lead to misinterpretation, since SAR is a measure of burial rate and may not be the preservation efficiency. Organic carbon also undergoes significant influences from other biological processes before final burial, beyond sedimentation rate alone. To further increase the confidence in these findings, I wonder if authors can validate their calculations against available in-situ data (e.g., from published studies) or discuss the potential uncertainties.

Response: Thank you. We agree that the MCBP is a first-order simplification and it inherently reduces the complex fate of organic carbon. We have addressed your comments point-by-point below:

Regarding your first concern, we fully acknowledge that in hyper-dynamic environments such as 'mobile muds', organic carbon preservation may be uncoupled from SAR. We have strengthened the discussion of this phenomenon (Lines 129-134):

However, while SAR serves as a robust proxy for physical trapping, it does not perfectly equate to net organic carbon preservation efficiency. In specific hyper-dynamic environments, such as high-energy mobile muds (e.g., the Amazon–Guianas mudbelt) and strongly resuspended large river estuaries, intense physical reworking exposes organic matter to prolonged oxic degradation. In such settings, high gross SAR is uncoupled from long-term preservation, leading to massive carbon remineralization. Consequently, our MCBP metric may overestimate the realized carbon sink in these specific high-energy regimes.

Regarding your second concern, we have revised this phrasing to "geological burial capacity" or "physical trapping potential".

Regarding your third concern, we agree that organic carbon is significantly influenced by other biological processes (e.g., decomposition) prior to final burial. We have further strengthened the quantification and discussion of these biological limitations: we quantified the global sensitivity of macroalgal biomass degradation driven by Sea Bottom Temperature (SBT). This revealed significant spatial heterogeneity in theoretical retention (Supplementary Methods 3; Supplementary Fig. 7).

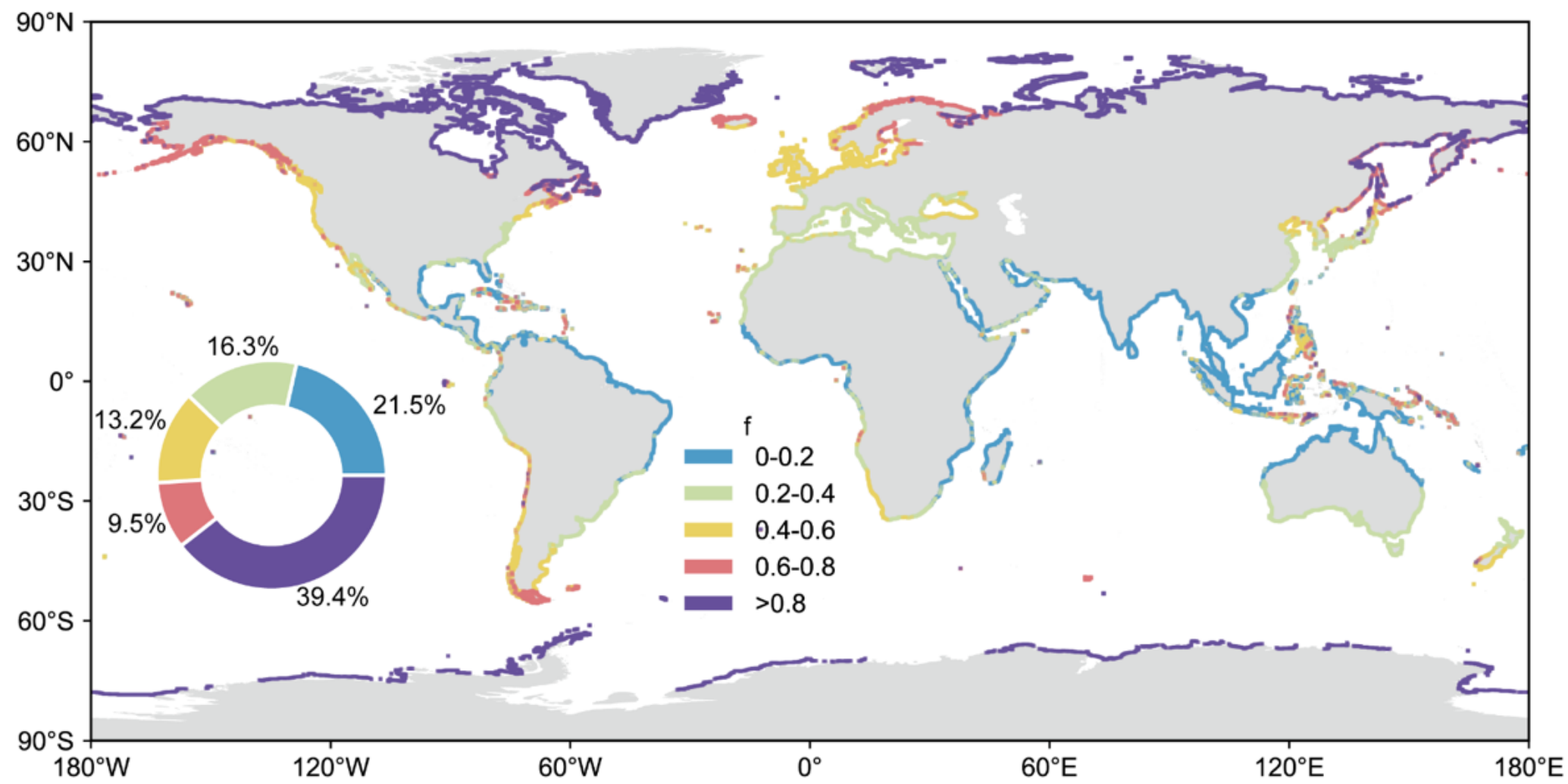

Supplementary Fig. 7. Spatial sensitivity analysis of the theoretical thermal retention factor (f) for macroalgal carbon.

Moreover, we have fully discussed this mechanism in the revised manuscript (Lines 219-232):

Finally, our current framework focuses on quantifying the physical burial potential and omits critical biological and environmental loss pathways prior to ultimate sequestration. The ultimate fate of macroalgal carbon is heavily modulated by consumption by local benthic fauna (e.g., grazing by sea urchins) and temperature-dependent microbial decomposition. Because spatially explicit global datasets for benthic grazing pressure do not currently exist, a full parameterization of these biological losses remains unfeasible. Our theoretical sensitivity analysis based on a standard Q_{10} *thermal degradation model (Supplementary Methods 3; Supplementary Fig. 7) demonstrates that tropical burial hotspots could experience substantial thermodynamic MCPB losses due to rapid remineralization. In contrast, high-latitude cold-water sinks can effectively reduce degradation rates, resulting in up to a tenfold difference in theoretical retention efficiency between polar and equatorial regions. Nevertheless, the high depositional environments characteristic of mid-to-low latitudes may effectively counterbalance this thermodynamic penalty. The rapid mineral input associated with high SAR facilitates the swift burial of detritus below the active oxic zone, thereby shielding organic matter from rapid microbial decay. Future iterations of global macroalgal blue carbon models must prioritize the integration of spatially resolved benthic food-web dynamics, local diagenetic processes, and high-resolution coastal hydrodynamics to transition from mapping physical burial potential to verifying precise carbon fluxes.*

Comment 5: Equal-interval binning strategy
Figure 3c employs an equal-interval binning strategy. What is the rationale for adopting this grouping approach? The gray scatter points in the figure suggest no apparent correlation between shoreline complexity and MCBP. Would the results differ if different bin widths or grouping intervals were chosen?

Response: The primary rationale for adopting a binning strategy is to statistically identify and emphasize the underlying robust trend between coastline fractal dimension (D) and MCBP. This macro-geomorphological trend might otherwise be obscured by the inherent noise and heterogeneity present in raw environmental data. By aggregating data within defined intervals, binning reduces the influence of local variations, thereby revealing the underlying robust trend that might be less visually apparent in a highly dispersed raw scatter plot. This approach is a widely accepted and standardized practice across various scientific disciplines for identifying and quantifying relationships in noisy datasets[1]. Moreover, we have

evaluated the robustness of our results to different binning strategies. We tested various bin widths (ranging from 5 to 15 bins) and distinct grouping intervals (e.g., equal-interval and equal-frequency/quantile binning). Our sensitivity analyses consistently show that while the R^2 value may fluctuate slightly depending on the specific binning parameter, it generally remains robust at approximately 0.50 or higher, and almost all iterations maintain strict statistical significance ($p < 0.05$). We have now included this sensitivity analysis in the Supplementary Information (Supplementary Methods 5; Supplementary Fig. 10).

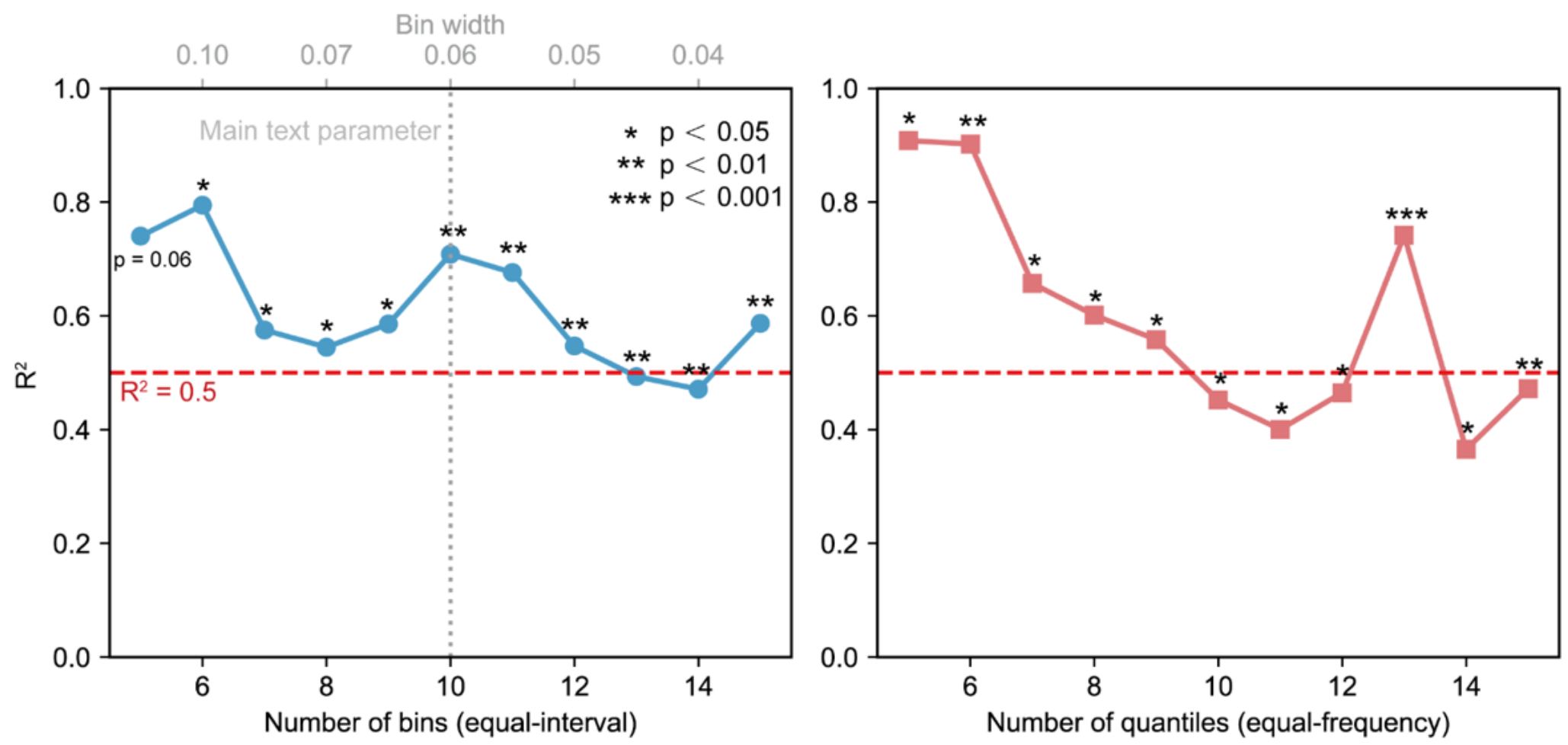

Supplementary Fig. 10. Sensitivity analysis of the correlation between coastline fractal dimension and MCBP to different binning strategies.

Comment 6: The content in Lines 108-112 of the Results section has already been described in the Methods section.

Response: Removed.

Comment 7: Line 93, "Results" or "Results and Discussion"?

Response: Changed to "Results and discussion".

Comment 8: Line 389: I am unclear about the meaning of "weighted equally" here. Does multiplying the two factors imply equal weighting in the subsequent formula?

Response: By using the phrase "weighted equally", we intended to express that both factors (NPP and SAR), after being normalized and multiplied, have an equivalent mathematical contribution to the final MCBP. To provide a more accurate explanation, we have refined this statement (Lines 421-423):

This standardization rescaled all values to a dimensionless range of [0, 1], thereby ensuring that both biological supply (NPP) and geological burial (SAR), as key co-limiting factors, have an equal contribution to the MCBP without bias from their original magnitude scales or units.

Comment 9: I hope the authors find this review informative and helpful.

Response: We are deeply grateful for your invaluable contribution that improved our work.

Responses to comments from Reviewer #2:

Comment 1: This study assesses the storage (burial) potential of organic carbon originated from macroalgal primary production in coastal areas around the globe at high resolution (5-arcmin) by model simulations. The results also provide some recommendations for national blue carbon management measures in coastal areas around the world. The study presents important concepts for establishing assessment methods to identify areas where macroalgal carbon storage is most effective for climate change mitigation, such as the creation and restoration of seaweed beds and the promotion of seaweed farming. However, I think that careful and humble attitudes are necessary when discussing the methodologies and results of this study.

Response: Thank you for the positive evaluation of our work. In response to your advice, we have more deeply integrated a careful and humble attitude into the revised manuscript, emphasizing the assumptions involved and its limitations.

Comment 2: This study is extremely challenging, but the results also have significant uncertainty. The calculation of MCBP in this study uses two parameters: a global-level estimated NPP from a previous study (Pessarrodona et al. 2021) and an original estimated SAR by the authors. However, both parameters are estimates and have significant uncertainties. Actually, I reside in a country rated as having a high MCBP in this study, but the results displayed in the figure do not necessarily match the results of our related research results and our own experiences. The manuscript also notes on the contradiction between that the results of this study suggesting that high-latitude regions are MCDP cold spots and previous literatures in which fjord areas are suggested to serve as a reservoir of macroalgal organic carbon (L159-163). Authors mention that this discrepancy is attributed to the resolution of this method, however, if there is any evidence in previous studies that could support or strengthen the results, please provide it as much as possible.

Response: Thank you. We have addressed the concerns point-by-point below:

1.We have implemented a comprehensive uncertainty analysis and adopted conservative data-handling strategies to explicitly address the uncertainties in both SAR and NPP parameters.

1) Quantifying SAR uncertainty: We conducted a rigorous uncertainty analysis using a 100-iteration Bootstrapping approach (detailed in Supplementary Methods 3) and then calculated the Coefficient of Variation (CV = standard deviation / mean) for every coastal grid cell to quantify the relative predictive uncertainty (Supplementary Fig. 6). The results validate the robustness of our model across the majority of global coastlines. Specifically, the majority of grid cells across most temperate and tropical coastlines exhibit low to moderate relative uncertainty, with the CV concentrated between 0 and 0.3 (77.6%). As expected, higher predictive uncertainty (CV > 0.3) is predominantly clustered in under-sampled, high-latitude regions (e.g., the Russian Arctic coast).

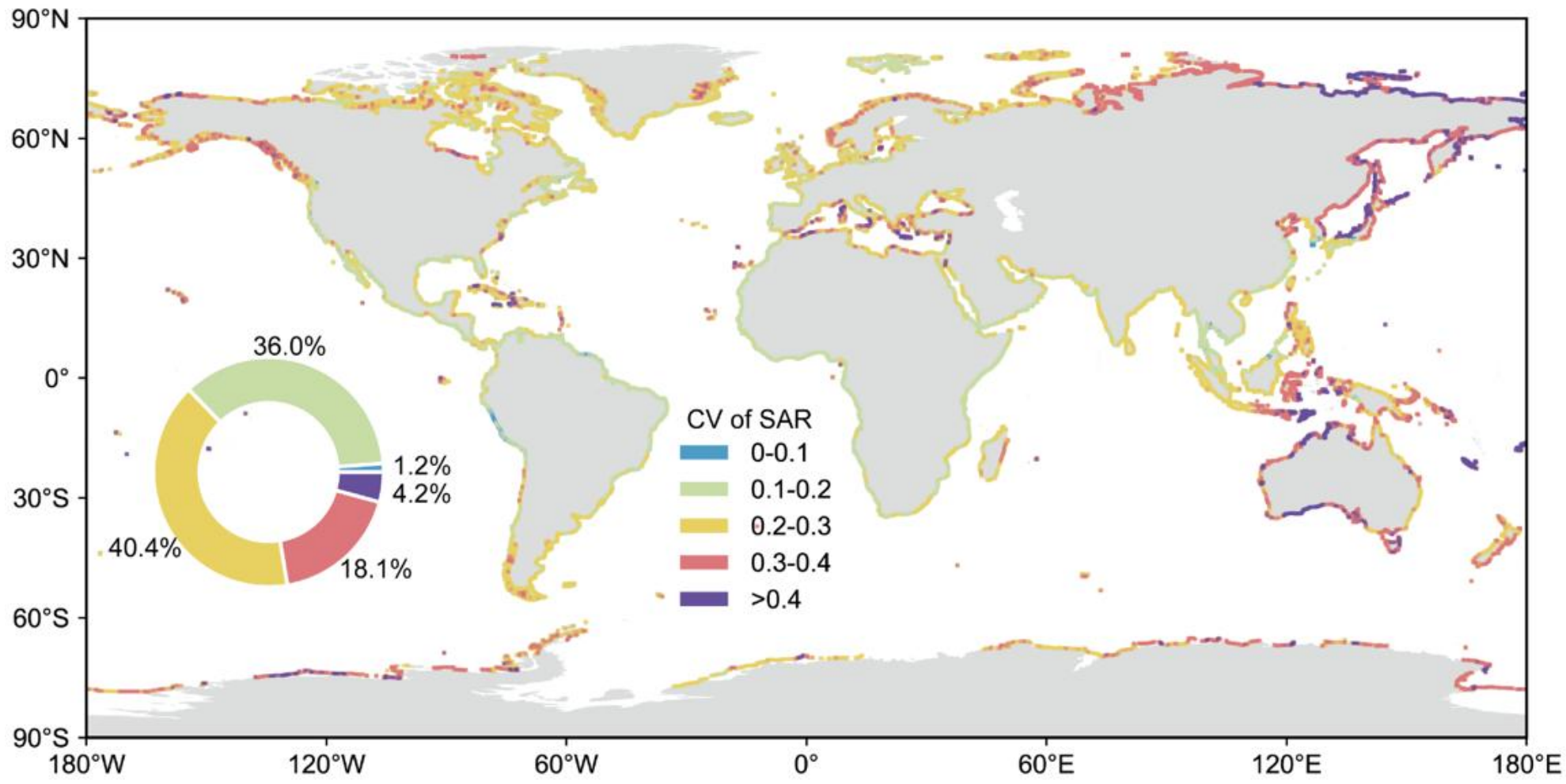

Supplementary Fig. 6. Spatial uncertainty of the global coastal Sediment Accumulation Rate (SAR) predictions.

2) Constraining NPP uncertainty: We fully acknowledge that the global NPP estimates (Pessarrodona et al., 2021) carry inherent uncertainties derived from their ecological niche models. Because this is a third-party product, we cannot perform a pixel-wise bootstrapping error propagation. Therefore, to strictly limit the propagation of extreme model artifacts, we adopted a data-handling approach raised up in recent macroalgal carbon assessments (e.g., Filbee-Dexter et al., 2025, Nature Geoscience[2]). Specifically, all predicted NPP values exceeding the 95th percentile were truncated to the value at the 95th percentile prior to calculation.

Crucially, the uncertainties associated with both the modeled SAR and NPP have now been comprehensively discussed in the revised manuscript (Lines 147-154):

However, the foundational data driving this macro-scale framework inevitably rely on global modeling and carry inherent uncertainties. These uncertainties in both biological and geological predictions are significantly amplified in data-sparse regions, particularly along polar coastlines and the African continent. Our spatial uncertainty analysis of the SAR predictions (Supplementary Methods 3; Supplementary Fig. 6) corroborates this: while SAR predictions are highly stable across most temperate and tropical coastlines (Coefficient of Variation, CV <0.3), significant uncertainties persist in under-sampled regions (e.g., the Russian Arctic coast). This highlights critical geographical data gaps where models are forced to rely on extrapolation, underscoring the need for future empirical sampling.

2. We believe the discrepancy between our national-level assessments and your observations may stem from the following two reasons:

1) Our national-level assessments (e.g., Figure 4) are designed to evaluate the aggregated average capacity of a nation's entire coastline. Due to the high heterogeneity of coastlines, even within a country defined as having a high MCBP, it does not preclude the existence of extensive local cold spots within that same jurisdiction.

2) The NPP we utilized (Pessarrodona et al., 2021) is based on ecological niche modeling, which calculates the potential productivity of macroalgae given suitable environmental conditions (e.g., light, temperature, nutrients), regardless of whether a dense kelp forest currently exists at that exact 5-arcminute pixel. Consequently, in certain grid cells, the actual standing stock of macroalgae might be currently depleted (e.g., due to local pollution or historical harvesting), yet our model still assigns a high MCBP value to indicate the

capacity of that area to act as a carbon sink if restored or utilized for aquaculture.

3. Regarding the model's performance in polar regions, we provide the following detailed explanation and have revised the manuscript, supported by literature:

1) On a global scale, high-latitude regions generally exhibit low MCBP. This is primarily driven by severe biological constraints: macroalgal NPP is heavily restricted by seasonal light availability, persistent low temperatures, and extensive sea ice cover[3-5]. Even though the SAR on polar open shelves is only slightly below the global average (Fig. 1b, 2c), the resulting coupled MCBP remains inherently low across these vast regions (Fig. 1a, 2c).

2) Conversely, fjords function as high efficient particulate matter sinks[6]. They operate through a dual mechanism: restricting the offshore export of in-situ macroalgal production[7], while simultaneously receiving massive allochthonous detrital subsidies funneled from external habitats[8]. This perfectly explains the strong macroalgal carbon and eDNA signatures found in deep fjord sediments[9,10]. However, we fully acknowledge two methodological constraints: First, due to the 5-arcminute spatial resolution and the scarcity of empirical data in polar regions, our model cannot fully decouple these micro-scale fjords from the surrounding vast open shelves to manifest them as distinct localized hotspots. Second, our methodology, which couples local in-situ NPP and SAR, does not explicitly account for the cross-pixel funneling of allochthonous subsidies. Importantly, our study does not classify widespread, fjord-dense coastlines as cold spots. As shown in Fig. 2a, regions characterized by extensive fjord networks (such as coastal Norway and coastal Greenland) are actually identified by our model as areas of moderate burial potential (Fig. 1a).

We have revised the Results and discussion section (Lines 174-187) as follows:

Conversely, high-latitude regions (60°–90° N/S) generally manifest as co-coldspots (Fig. 2a, 2b). On a global scale, this macro-regional pattern is primarily driven by severe biological constraints (e.g., seasonal light limitation, persistent low temperatures and extensive sea ice cover) that restrict the spatial extent and annual productivity of macroalgae coupled with open glacial shelves where the SAR remains below the global average. However, it is critical to distinguish these vast open shelves from localized topographic traps such as fjords[44]. Fjords function as highly efficient macroalgal particulate sinks through a dual mechanism: they restrict the offshore export of in-situ macroalgal biomass while simultaneously receiving massive allochthonous detrital subsidies funneled from external coastal habitats. This dual input mechanism explains the strong macroalgal carbon and eDNA signatures frequently observed in polar fjord sediments. Importantly, our spatial model does not uniformly classify widespread, fjord-dense coastlines (e.g., Norway and coastal Greenland; Fig. 2a) as cold spots. Nevertheless, we acknowledge that due to the 5-arcminute resolution and empirical data scarcity, our framework cannot fully decouple micro-scale fjords from surrounding open shelves to highlight them as distinct regions. Furthermore, our local-coupling methodology does not explicitly route cross-pixel allochthonous subsidies. Therefore, future high-resolution hydrodynamic models are required to accurately resolve these sub-grid depositional dynamics.

Comment 3: Furthermore, the fate of macroalgal primary production in coastal areas involves biological processes such as decomposition and being consumed by herbivorous animals. These processes are likely to be significantly affected by factors such as water temperature and local fauna, which varies greatly depending on latitude. Therefore, these factors are considered to affect the amount of macroalgal organic matter being deposited on the seafloor of coastal areas, and thus the MCDP. Although I acknowledge that it is currently very difficult to incorporate these factors into the model, discussing burial potential solely in

terms of NPP and SAR is too simplistic and is considered to be a major source of uncertainty in the results obtained. Therefore, I think these parameters (decomposition and consumption) should be incorporated into future updates of this method. In order to improve the accuracy of the results of this study, I think there should be further discussion about what improvements and information are needed in the future.

Response: Thank you. To address these potential uncertainties, we have conducted feasible quantifications and provided detailed discussions.

1. Quantitative sensitivity analysis of temperature-driven decomposition

To quantitatively explore the uncertainty introduced by microbial decomposition, we conducted a theoretical sensitivity analysis (Supplementary Methods 3 and Supplementary Fig. 7).

Recent empirical evidence, such as the field experiment by Filbee-Dexter et al.[11] across 35 northern hemisphere sites, reveals that macroalgal detritus decomposition is highly temperature-dependent. Therefore, to robustly capture the global non-linear thermodynamics of microbial remineralization—which fundamentally follows Arrhenius kinetics as acknowledged by Filbee-Dexter et al.[11]—we applied the standard biological temperature coefficient ($Q_{10} = 2.0$) to formulate our relative thermal retention factor (*f*). Simply put, this assumes that the decomposition rate doubles for every 10 °C increase, a parameterization widely adopted in global soil and marine sediment organic carbon decomposition models[12,13].

The resulting map (Supplementary Fig. 7) shows that tropical MCBP hotspots face severe thermodynamic discounting due to rapid benthic remineralization, whereas high-latitude cold-water environments act as thermal refuges that maximize preservation efficiency.

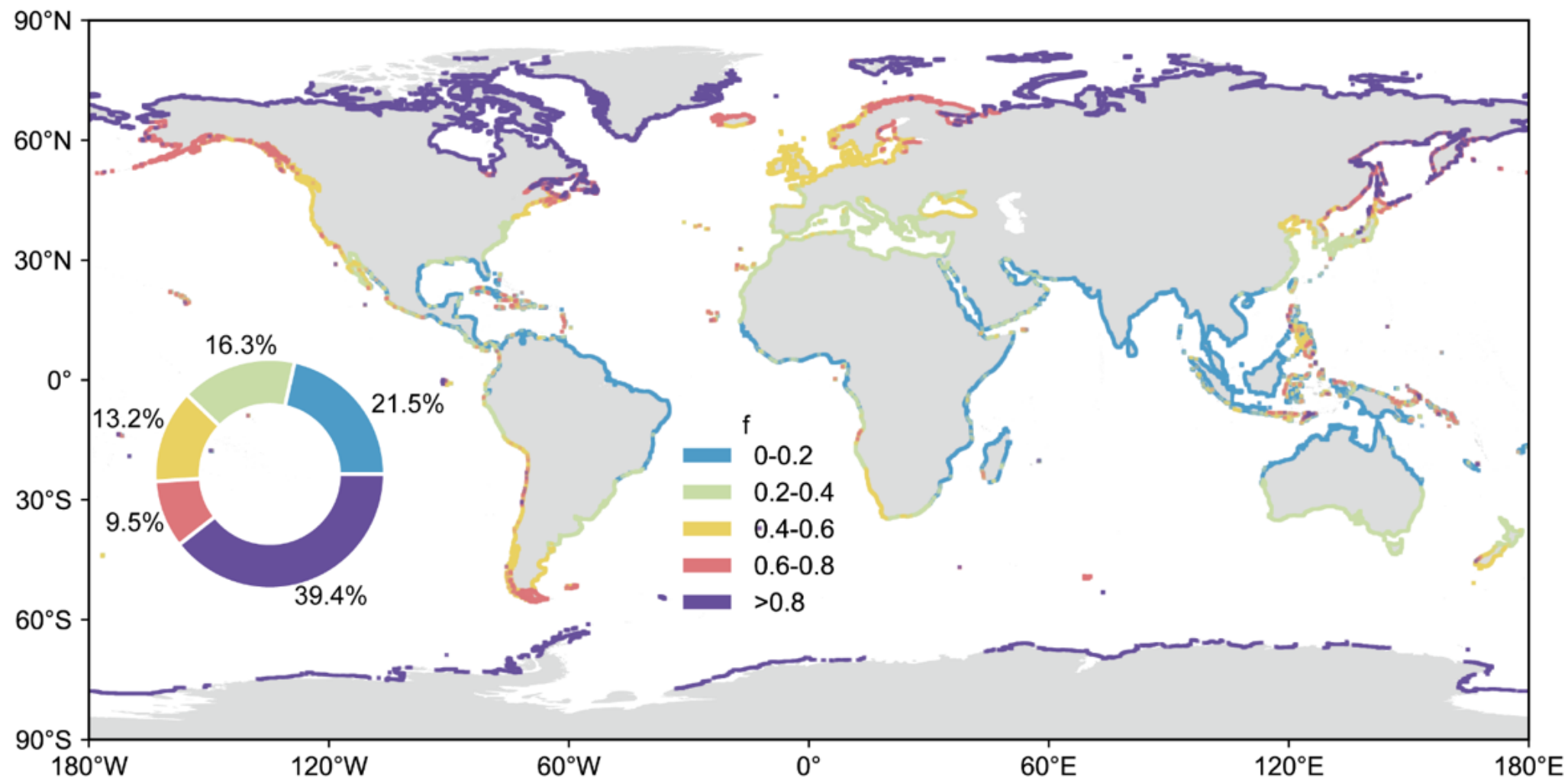

Supplementary Fig. 7. Spatial sensitivity analysis of the theoretical thermal retention factor (f) for macroalgal carbon.

2. The role of herbivory and future data needs

Regarding consumption by local fauna (e.g., grazing by sea urchins), we fully acknowledge this as a major source of uncertainty and incorporating this into the current model is unfeasible because global-scale datasets do not currently exist.

We have provided a detailed discussion in the revised manuscript (Lines 219-232):

Finally, our current framework focuses on quantifying the physical burial potential and omits critical biological and environmental loss pathways prior to ultimate sequestration. The ultimate fate of macroalgal carbon is heavily modulated by consumption by local benthic fauna (e.g., grazing by sea urchins) and temperature-dependent microbial decomposition. Because spatially explicit global datasets for benthic grazing pressure do not currently exist, a full parameterization of these biological losses remains unfeasible. Our theoretical sensitivity analysis based on a standard Q_{10} thermal degradation model (Supplementary Methods 3; Supplementary Fig. 7) demonstrates that tropical burial hotspots could experience substantial thermodynamic MCBP losses due to rapid remineralization. In contrast, high-latitude cold-water sinks can effectively reduce degradation rates, resulting in up to a tenfold difference in theoretical retention efficiency between polar and equatorial regions. Nevertheless, the high depositional environments characteristic of mid-to-low latitudes may effectively counterbalance this thermodynamic penalty. The rapid mineral input associated with high SAR facilitates the swift burial of detritus below the active oxic zone, thereby shielding organic matter from rapid microbial decay. Future iterations of global macroalgal blue carbon models must prioritize the integration of spatially resolved benthic food-web dynamics, local diagenetic processes, and high-resolution coastal hydrodynamics to transition from mapping physical burial potential to verifying precise carbon fluxes.

Comment 4: As mentioned above, there is still a great deal of uncertainty in the results. So I recommend that assessing MCDP on a country-by-country basis as shown in Figure 4 should be reconsidered its suitability. Also, Figure 4 shows income levels, but is there any point in showing them in this study? At best, I think the results should be expressed in larger regional groups, such as East Asia or the Atlantic coast of North America (as examples), and discussion of country-by-country assessments should be scaled down.

Response: Thank you. We agree that extending the implications of our geophysical model into complex socio-economic typologies is not appropriate.

We have completely removed the socio-economic analysis (former Figure 4c) and its associated discussion. Furthermore, we have scaled down the discussion on country-by-country assessments and incorporated a continent-scale assessment of MCBP (Lines 283-286):

The global capability for macroalgal carbon burial reveals a stark geopolitical and geographical asymmetry. At the continental scale, macroalgal blue carbon capacity is highly unevenly distributed, with Asia and North America collectively accounting for approx. 42.0% and 21.2% of the global total MCBP, respectively, while regions such as Europe, South America and Africa contribute comparatively less (15.4%, 11.3% and 4.1%).

Responses to comments from Reviewer #3:

Comment 1: **Significance**

This work, is from what I am aware, novel in its approach and conclusions and would be a great addition to the literature and community. The approach is interesting and provides a potentially valuable framework for identifying critical areas of potential macroalgal C burial. However, careful consideration should be made to the underlying assumptions made in conducting the modeling here as there seems to be sparse discussion/inclusion of error/uncertainties in their conclusions in the body of the text itself - I think it's important to disclose and discuss as we move towards a better understanding of algal standing stocks, NPP, and C cycling/sequestration as a field - transparency is key.

Response: Thank you for the positive evaluation of our work. Addressing your comment regarding transparency, we have enhanced the discussion of underlying assumptions, errors, and inherent uncertainties throughout the revised manuscript, as explained below.

Comment 2: **Key results**

This work presents an investigation of macroalgal carbon burial potential modeled globally, outlining the importance of macroalgae as a potential nature-based climate mitigation solution and the results draw attention to regions/nations where potential burial hot spots do not line up with marine protected areas - indicating room for improvement and suggesting this research might serve as a potential framework to make said improvements.

- Two main outcomes/products of the work are 1) a proxy built for identifying high potential macroalgal C burial areas using coastal complexity (fractal dimension) and 2) the identification of the mismatch between high potential C burial regions and MPAs and suggestion for improved alignment.

Response: Thank you.

Comment 3: **Validity**

The study seems to provide a robust analysis of the data collected and used for their models.

One very important point, which is made only in the methodology section of the paper is the data used especially for the macroalgal NPP underpinning the entire analysis. The authors are using a modeled product or modeled estimates of macroalgal NPP to determine their MCBP metric, which should - in my opinion - be outlined very clearly in the paper as these estimates come with high levels of uncertainty.

In addition, the definition of macroalgal NPP and carbon in general for that matter are not very well defined in the body of the text. How are the authors defining macroalgal NPP? Macroalgal NPP might very well include dissolved organic C production, does it in this case? If it does, how does that contribute to coastal carbon burial, when we know DOC will either be remineralized or be sequestered to the deep oceans? These distinctions and definitions should be more explicitly outlined in the main body of the text.

Response: Thank you. Regarding your first point, we have now explicitly mentioned the origin of NPP data, and comprehensively discussed the uncertainty inherent in the modeled NPP data in the revised manuscript

(Lines 147-150):

However, the foundational data driving this macro-scale framework inevitably rely on global modeling and carry inherent uncertainties. These uncertainties in both biological and geological predictions are significantly amplified in data-sparse regions, particularly along polar coastlines and the African continent.

Regarding your second point, total macroalgal NPP encompasses both the particulate fraction (POC/detritus) and the dissolved fraction (DOC). In the context of coastal sedimentary burial (which is governed by the SAR), the relevant carbon pool is almost exclusively the particulate fraction (POC and stochastically detached whole biomass). While macroalgae exude substantial DOC, the vast majority of this fraction is rapidly remineralized in the water column or exported offshore, and thus does not primarily contribute to the coastal sedimentary sink we modeled.

We have refined the terminology in the Introduction section (Lines 63-67):

The spatial distribution of macroalgal sedimentary carbon sequestration in coastal sediments is governed by a sequential, biogeochemical framework comprising three primary factors: (i) the initial production of macroalgal-derived detrital particulate organic carbon (POC; herein defined broadly to encompass both fragmented macroalgal particles and larger, stochastically dislodged macroalgal biomass) at the source, (ii) the efficiency of adjacent depositional environments as long-term sinks, and (iii) the hydrodynamic transport pathways that connect them.

Moreover, we have emphasized it in the Methods section (Lines 429-436):

It is important to clarify the biogeochemical nature of the carbon pools represented in our framework. The modeled macroalgal NPP estimates utilized here represent total primary production, which inherently encompasses both particulate organic carbon (POC; fragmented macroalgal particles and larger, stochastically dislodged macroalgal biomass) and dissolved organic carbon (DOC) exuded during growth. However, our MCBP metric is fundamentally constrained by the physical trapping mechanism of SAR, which primarily acts upon the settling particulate fraction. In contrast to DOC, whose long-term sequestration primarily relies on export below the mixed layer or transformation into refractory DOC (RDOC), the MCBP index effectively represents the physical favorability of coastal environments to trap and sequester the particulate fraction (detritus and dislodged biomass) of the local macroalgal production.

Comment 4: **Data and methodology**

The handling of data in general is appropriate, and from limited knowledge of the particular model used in the study, the rationale and execution seem well founded.

Response: Thank you.

Comment 5: **Suggested improvements**

Improved transparency on the macroalgal NPP data underpinning the results should be carefully considered, especially as the authors are suggesting this work be used by policy-makers for designations of MPAs in the future that better encapsulate a co-benefit of MPA designation - macroalgal C burial/sequestration.

Response: Thank you. To improve the transparency of the macroalgal NPP data underpinning our results, we have made the following explicit clarifications in the revised manuscript:

1. The data are derived from global model estimates based on ecological niche modeling (Pessarrodona et al., 2021). This approach is now explicitly stated (Lines 144-147):

Because the underlying macroalgal NPP estimates (Pessarrodona et al.) are derived from ecological niche models predicting potential productivity based on environmental suitability, high-MCBP regions effectively identify optimal environments for burial, even if current biomass is locally depleted due to e.g., historical harvesting or overgrazing.

2. To prevent extreme model artifacts, we applied the same conservative data-handling procedure to this dataset as used in previous macroalgal carbon sequestration estimates (Filbee-Dexter et al., 2025, Nature Geoscience[2]). This operation is now clearly outlined and clarified in the Methods section (Lines 414-416):

To mitigate the influence of extreme model artifacts and ensure conservative estimates, NPP values were capped at the 95th percentile prior to analysis; specifically, all predicted values exceeding this threshold were reassigned to the value at the 95th percentile.

3. Regarding the uncertainty of these data, we have discussed the uncertainties of the model outputs in data-sparse regions within the revised manuscript. For detailed information, please refer to our response to your Comment 3.

Comment 6: **References**

The references seem appropriate.

Response: Thank you.

Comment 7: Line 28: 5 arcmin is fine, however, might be misleading to the reader as higher res than it really is (10km) - maybe should be expressed in km for this reason?

Response: Expression of km added.

Lines 27-29: *Here, we present a high-resolution (5-arcmin, approx. 9.2 km at the equator)...*

Comment 8: Line 49: with respect to discussion on refractory DOC - yes and DOC is released throughout growth so would/should be considered in the calculation of any C sink — where does DOC come into play in your macroalgal NPP dataset? Is it considered or excluded from these numbers? This should be made clear.

Response: Thank you. Please refer to our response to your Comment 3 for details.

Comment 9: Line 66: not only does detrital C serve as a pool of C with sequestration potential, but the algal biomass itself is stochastically 'sunk' or transported as well.. unless you are looping this into your definition of detrital material - which in this case should be clearly defined

Response: Thank you. The stochastic dislodgement and subsequent sinking of whole algal biomass is indeed an important pathway for macroalgal carbon export and burial, particularly following storm events. We have now explicitly defined "detritus" in the revised manuscript to encompass both fragmented algal particles and larger dislodged algal biomass.

The specific revisions addressing this definition have been incorporated into the Introduction section (Lines 63-67). For the detailed text of this modification, please refer to our response to your Comment 3.

Comment 10: Line 70: what happens in low SAR ... could speak briefly on biodegradation of the POC in these settings and the prevention of sequestration?

Response: Thank you. We have now added a brief discussion (Lines 76-78):

Conversely, in low SAR environments, macroalgal POC remains exposed at the sediment-water interface for extended periods, leading to rapid microbial biodegradation and remineralization, which effectively reduces long-term sequestration.

Comment 11: Line 73: is there any ground-truthed data/areas in the literature that show this connection/process? if so could cite it here to solidify point

Response: Thank you. We have incorporated several key field studies that demonstrate the massive production of macroalgal detritus and its subsequent capture by adjacent depositional environments. These studies serve as empirical validations of our spatial coupling framework across diverse geographical areas, including: South Africa[15], The United Kingdom[16], Australia[17], Greenland[7]. Furthermore, we have cited a foundational review that comprehensively describes this detrital production and fate mechanism[18]. Finally, to provide direct evidence that favorable depositional environments form substantial long-term macroalgal carbon sinks, we cited a recent global empirical study on seaweed aquaculture, which confirms that carbon burial in sediments below seaweed farms matches that of traditional blue carbon habitats[19].

Comment 12: Line 114: since this is a big factor you should mention it earlier in the study and drive home that your modeling is accounting for it

Response: Thank you. We have moved the explanation of this factor into the Introduction section (Lines 68-71):

Because macroalgae predominantly colonize hard substrates where in situ sedimentation is negligible, their long-term burial relies entirely on this spatial coupling. Therefore, accurately mapping this carbon sink requires a framework that evaluates the capacity of nearby soft-sediment systems to trap detritus exported from proximal hard-substrate habitats.

Comment 13: Line 126: is this not well known that riverine estuaries are high depositional regions? your findings support that knowledge... should cite pioneering work on this

Response: Thank you. We have incorporated citations to pioneering works and revised the phrasing in the manuscript (Lines 127-129).

These findings align with foundational marine geological paradigms, confirming that favorable depositional

environments for blue carbon are not uniformly distributed but are highly concentrated in specific river-dominated estuaries, semi-enclosed bays, and extensive continental shelves[20-22].

Comment 14: Line 142: do you have map of seaweed NPP based on your collection of modeled estimates? could include it in the SI

Response: Thank you. We have now generated a global map of the macroalgal NPP used in our analysis. This map has been included in the Supplementary Information (Supplementary Fig. 5).

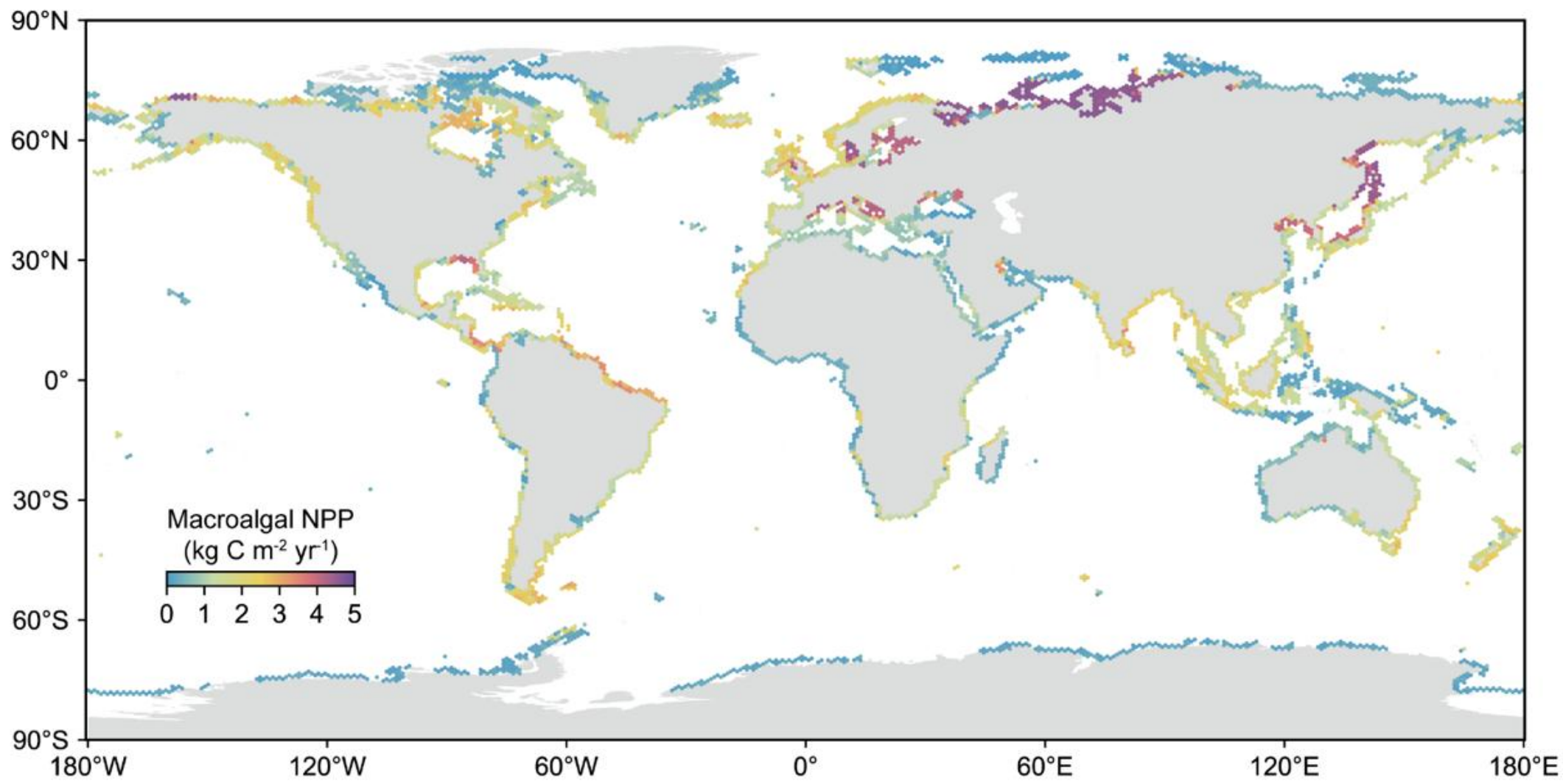

Supplementary Fig. 5. Spatial distribution of the modeled global macroalgal Net Primary Productivity (NPP).

Comment 15: Line 179: should this read "… potential coastal carbon burial…" instead? personal preference

Response: Corrected.

Comment 16: Line 212: can you include the sample number (n = ?) for this correlation

Response: Thank you. We have included the sample number (n=10) for this correlation.

Comment 17: Line 220: are you just talking about POC (defined how?) in this study or also entire algal biomass? where is this defined if at all?

Response: Thank you. Within our modeling framework, we are primarily considering the coastal burial of POC. This POC pool is defined broadly to encompass both fine fragmented detritus and larger, stochastically dislodged whole algal biomass. For the specific text of these revisions, please refer to our detailed responses to your Comments 3 and 9.

Comment 18: Line 271: consequences of all this extra seaweed burial from aquaculture? could include one line on this. If harvested... can we count DOC and POC during growth towards C burial?

Response: Thank you. We have added the following sentence (Lines 297-299):

Even if the primary macroalgal biomass is ultimately harvested, the POC detached and the DOC during the growth phase remain quantifiable and significant sources of carbon sequestration.

Comment 19: Line 280: expand on this. it's important and a large barrier/constraint to progress

Response: Thank you. We have expanded on this (Lines 306-314):

However, unlocking this potential as a verifiable nature-based climate solution requires overcoming significant technical barriers to develop standardized frameworks for Monitoring, Reporting, and Verification (MRV). Although existing evidence demonstrates that macroalgal aquaculture can significantly enhance carbon sequestration in underlying and adjacent sediments, the highly dispersive nature of macroalgal carbon demands more rigorous quantification. Further clarifying the net sequestration efficiency of exported detrital POC, alongside tracking the deep-ocean export of DOC and the formation of refractory DOC (RDOC), remains a critical challenge. Addressing these barriers will require the integration of fine-scale empirical field studies with high-resolution local hydrodynamic models to robustly track the ultimate fate of farmed carbon and maximize its climate mitigation potential.

Comment 20: Line 382: this is a really important point that underlies all of your findings. The use of modeled algal NPP has large uncertainties ----- how do they bare out in your analysis... nothing has error bars

Response: Thank you. We agree that both foundational datasets (NPP and SAR) carry inherent uncertainties.

Regarding our data processing and discussion of the modeled NPP data, please refer to our detailed responses to your Comments 3 and 5 above.

Regarding the SAR data, which we modeled independently, we have now fully quantified its spatial uncertainty and calculated the Coefficient of Variation (CV) for every global grid cell. This allows us to transparently present the relative predictive uncertainty of our model across the globe (Supplementary Fig. 6).

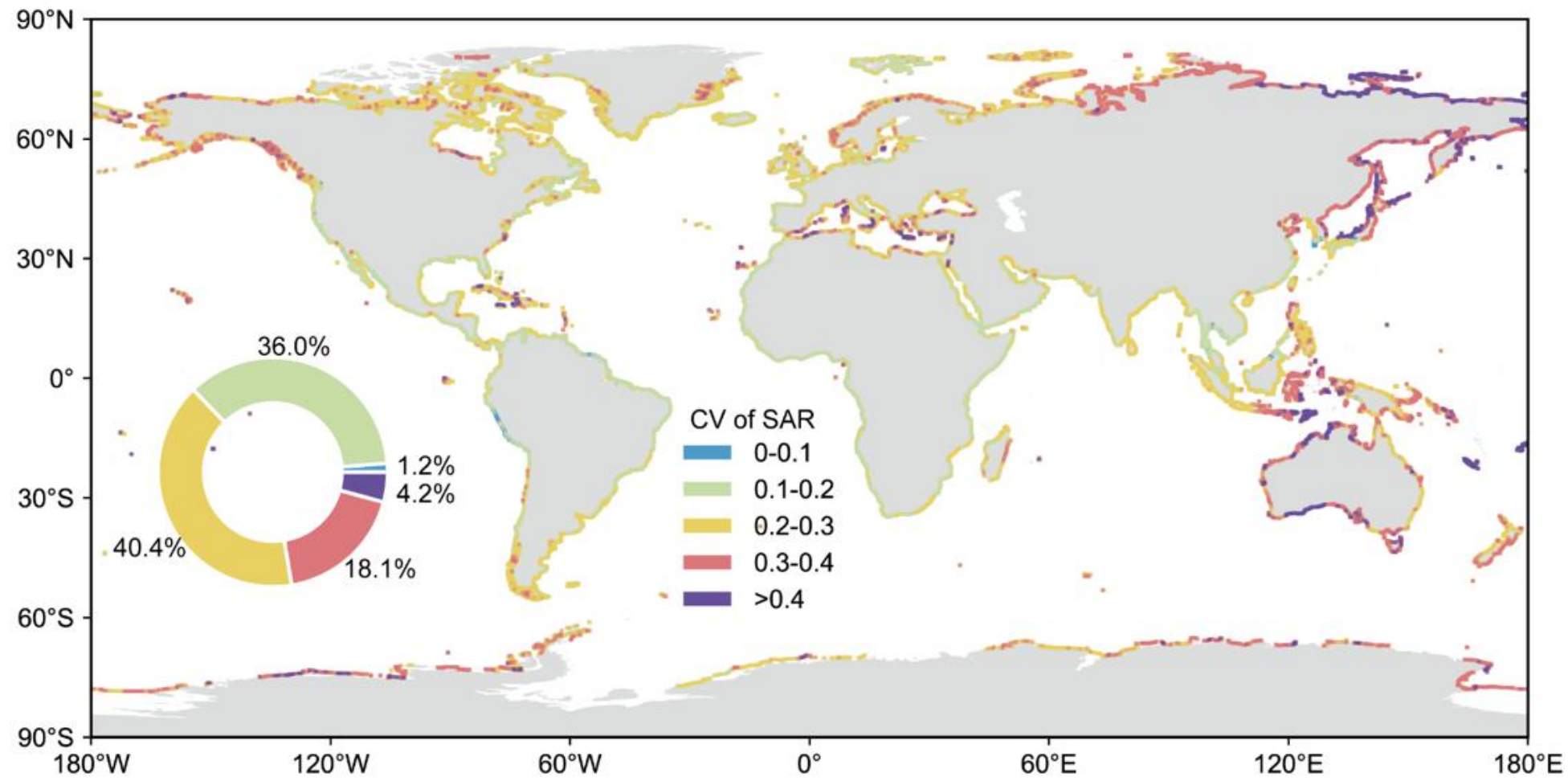

Supplementary Fig. 6. Spatial uncertainty of the global coastal Sediment Accumulation Rate (SAR) predictions.

Comment 21: I am an R user, so review of the python code was outside my expertise.

Response: Thank you. We confirm that the phyton code used is correct.

References cited in the response letter:

1. Xiao, X. et al. Resource (Light and Nitrogen) and Density-Dependence of Seaweed Growth. *Front. Mar. Sci.* **6**, 618 (2019).
2. Filbee-Dexter, K. et al. Carbon export from seaweed forests to deep ocean sinks. *Nat. Geosci.* **17**, 552–559 (2024).
3. Krause-Jensen, D. et al. Seasonal sea ice cover as principal driver of spatial and temporal variation in depth extension and annual production of kelp in Greenland. *Global Change Biol.* **18**, 2981–2994 (2012).
4. Pessarrodona, A. et al. Global seaweed productivity. *Sci. Adv.* **8**, eabn2465 (2022).
5. Gómez, I. et al. Light and temperature demands of marine benthic microalgae and seaweeds in polar regions. *Bot. Mar.* **52**, 593–608 (2009).
6. Smith, R. W., Bianchi, T.S., Allison, M., Savage, C. & Galy, V. High rates of organic carbon burial in fjord sediments globally. *Nat. Geosci.* **8**, 450–453 (2015).
7. Ager, T. G. et al. Macroalgal habitats support a sustained flux of floating biomass but limited carbon export beyond a Greenland fjord. *Sci. Total Environ.* **872**, 162224 (2023).
8. Filbee-Dexter, K., Wernberg, T., Norderhaug, K. M., Ramirez-Llodra, E. & Pedersen, M. F. Movement of pulsed resource subsidies from kelp forests to deep fjords. *Oecologia* **187**, 291–304 (2018).
9. Ørberg, S. B. et al. Prevalent fingerprint of marine macroalgae in arctic surface sediments. *Sci. Total Environ.* **898**, 165507 (2023).
10. Ørberg, S. B. et al. Millennial-scale fingerprint of macroalgae in Arctic marine sediments. *Sci. Total Environ.* **998**, 16 (2010).
11. Filbee-Dexter, K. et al. Kelp carbon sink potential decreases with warming due to accelerating decomposition. *PLOS Biol.* **20**, e3001702 (2022).
12. Arndt, S. et al. Quantifying the degradation of organic matter in marine sediments: A review and synthesis. *Earth Sci. Rev.* **123**, 53–86 (2013).
13. Davidson, E. A. & Janssens, I. A. Temperature sensitivity of soil carbon decomposition and feedbacks to climate change. *Nature* **440**, 165–173 (2006).
14. Krause-Jensen, D. & Duarte, C. M. Substantial role of macroalgae in marine carbon sequestration. *Nat. Geosci.* **9**, 737–742 (2016).
15. Bustamante, R. H., Branch, G. M. & Eekhout, S. Maintenance of an Exceptional Intertidal Grazer Biomass in South Africa: Subsidy by Subtidal Kelps. *Ecology* **76**, 2314-2329 (1995).
16. Queirós, A. M. et al. Connected macroalgal-sediment systems: blue carbon and food webs in the deep coastal ocean. *Ecol. Monogr.* **89**, 21 (2019).
17. Wernberg, T., Vanderklift, M. A., How, J. & Lavery, P. S. Export of detached macroalgae from reefs to adjacent seagrass beds. *Oecologia* **147**, 692–701 (2006).
18. Krumhansl, K. A. & Scheibling, R. E. Production and fate of kelp detritus. *Mar. Ecol. Prog. Ser.* **467**, 281–302 (2012).
19. Duarte, C. M. et al. Carbon burial in sediments below seaweed farms matches that of Blue Carbon habitats. *Nat. Clim. Change.* **15**, 180–187 (2025).
20. Milliman, J. D. & Syvitski, J.P.M. Geomorphic/Tectonic Control of Sediment Discharge to the Ocean: The Importance of Small Mountainous Rivers. *J. Geol.* **100**, 525–544 (1992).
21. Burdige, D. J. Burial of terrestrial organic matter in marine sediments: A re-assessment. *Global Biogeochem. Cy.* **19**, GB4011 (2005).
22. Hedges, J. I. & Keil, R. G. Sedimentary organic matter preservation: an assessment and speculative synthesis. *Mar. Chem.* **49**, 81–115 (1995).

REVIEWER COMMENTS

Responses to comments from Reviewer #1:

Comment 1: This version of the manuscript is greatly improved over the first version I looked at. It does a much better job of explaining what was done and resolving the issues I raised.

Response: We sincerely thank the reviewer for the positive assessment and for the constructive comments on the previous version, which substantially helped us to improve the manuscript.

Responses to comments from Reviewer #4:

Comment 1: I want to disclose that I was invited to review this manuscript after it had already been evaluated by previous reviewers, who were no longer available to assess the revised version. After a thorough examination, I found this to be an interesting paper: the authors integrate an impressive amount of data and have done a great job at addressing the comments. The figures are great and I appreciate the effort they have put into making the paper relevant to a management context by highlighting the fraction of high MCBP currently under any sort of managing or protection.

Response: We thank the reviewer for the time and care taken in reviewing our manuscript, and for the encouraging assessment of our work.

Comment 2: The paper adds to a recent wave of published machine-learning-based global maps and its corresponding criticisms (summarized in (Meyer & Pebesma, 2022). While I agree that a map of macroalgal carbon burial potential fills an important knowledge gap, I do think the approach taken has several weaknesses, and therefore its usefulness is compromised.

Response: We thank the reviewer for acknowledging that this work fills an important knowledge gap, and for providing constructive feedback that greatly contributed to improve our work. We are also grateful to the reviewer for bringing Meyer & Pebesma (2022) to our attention. In this revision, we have leveraged their framework concerning the limitations of machine-learning-based global mapping. We have also carefully addressed the specific weaknesses raised by the reviewer in our point-by-point responses below, and we have substantially strengthened the discussion of the limitations and appropriate scope of our approach throughout the revised manuscript.

Comment 3: As raised by other reviewers, the MCBP metric (NPP x SAR) misses several key processes that determine the fate of organic carbon burial in sediments. For example, we know that sediment mixing processes massively shape organic carbon burial rates and sequestration times. I appreciate the authors have acknowledged this as a limitation in this revision. However some of the MCBP predictions made here contrast with prevailing understanding of carbon burial potential in sediments, suggesting there are important processes that happen when carbon gets deposited/buried in sediment that are currently not captured. This ultimately limits the usefulness of the MCBP metric. For instance, the Yellow Sea coastlines have some of the highest predicted MCBP, but organic carbon freshly deposited in sediments in this area is known to have small burial efficiencies and permanence (Bradley et al., 2022; Song et al., 2016). Incorporating a meaningful measure of the potential fate of the buried accumulation rate therefore seems like an obvious way to improve its meaningfulness. This should be possible as there are now global products of sediment mixed layer depth (Song et al. 2022) or sediment burial/transfer efficiency (Bradley et al. 2022). At the very least the metric of temperature-dependent remineralization (Fig. S7) should be included in the MCBP metric.

Response: We thank the reviewer for this constructive suggestion and fully agree that incorporating burial/transfer efficiency, mixed-layer depth, or temperature-dependent remineralization would help to better represent the ultimate fate of macroalgal organic carbon in sediments. We have taken several steps to address these points.

First, we have incorporated the temperature-dependent remineralization factor into the metric (now denoted as MCBI, as detailed in our response to Comment 4) and mapped the resulting temperature-adjusted MCBI globally (Fig. 2c). The overall spatial pattern of temperature-adjusted MCBI remained strongly correlated with the original MCBI (Spearman ρ = 0.86, $p < 0.001$; Fig. 2d), indicating that the burial hotspots is robust to this modification. The latitudinal analysis showed that the temperature adjustment primarily reduced MCBI in the equatorial zone (−30° to 30°), while high-latitude hotspots remained largely unaffected, reflecting the stronger temperature-driven remineralization in warm tropical waters (Fig. 2e). This clarifies that MCBI hotspots are not simply an artifact of temperature-insensitive assumptions. Nevertheless, because

rapid burial associated with high SAR can shield organic matter from temperature-driven remineralization and this trade-off cannot yet be robustly parameterized at the global scale, we retained the original (unadjusted) metric for the main analyses in Results sections 3 and 4. The temperature-adjusted results are presented and discussed as an integral component of the second section of Results and Discussion (Fig. 2c–e), where we map the temperature-adjusted MCBI globally, quantify its correspondence with the original index, and examine the resulting latitudinal restructuring.

Second, regarding the incorporation of sediment mixed-layer depth (Song et al., 2022) and transfer efficiency (Bradley et al., 2022), we fully agree that these global products offer valuable constraints on post-depositional carbon fate. After careful examination, we noted that both metrics are heavily driven by SAR—Song et al. include SAR as a key predictor in their mixed-layer depth model, and the transfer efficiency of Bradley et al. is fundamentally a function of sedimentation rate and water depth. Since SAR is already a core component of our MCBI metric, including the sediment mixed-layer depth (Song et al., 2022) and transfer efficiency (Bradley et al., 2022) into our metric would introduce substantial redundancy by amplifying the same underlying SAR signal. Moreover, our index is designed as a relative indicator of burial favorability—quantifying the physical boundary conditions conducive to carbon burial—rather than an absolute predictor of long-term carbon sequestration. Therefore, we opted not to incorporate these products as additional constraints in our MCBI metric. To ensure transparency, we now explicitly discuss the potential regional implications of both sediment mixed-layer depth ad transfer efficiency in the revised Discussion (Lines 216-239), acknowledging that our current framework does not account for all post-depositional loss pathways.

Finally, we thank the reviewer for highlighting the apparent conflict between the high predicted MCBI in the Yellow Sea and its known low organic carbon burial efficiency and permanence. In our revised model results, the Yellow Sea coastline is no longer identified as a region of markedly high MCBI (Fig. 1a). This change primarily reflects the influence of unverified high SAR data concentrated in this region that was used in the original dataset. Thus, following the correction of the SAR dataset (see our response to Comment 6), the model no longer produces elevated MCBI predictions in the Yellow Sea. The concern raised by the reviewer is therefore resolved in the updated analysis.

We hope these revisions adequately address the reviewer's concerns.

Lines 216-239: *Our current framework focuses on quantifying the geophysical boundary conditions favorable for carbon burial, yet the realized long-term preservation of buried macroalgal carbon is further modulated by biological and environmental loss pathways prior to ultimate sequestration. Temperature-driven microbial decomposition is regarded as a key control on macroalgal organic carbon preservation: recent large-scale field evidence shows that macroalgal detritus decomposition is strongly temperature-dependent, and microbial remineralization fundamentally follows Arrhenius-type kinetics[1]. To explore the spatial heterogeneity this introduces, without over-parameterizing our dimensionless index, we conducted a theoretical sensitivity analysis by applying a standard biological temperature coefficient (Q_{10} = 2.0) to formulate a relative thermal retention factor (f)[2, 3], and incorporated it into the MCBI (Supplementary Methods 4, Fig. 2c). The temperature-adjusted MCBI remained strongly correlated with the original metric (Spearman $\rho = 0.86$, $p < 0.001$; Fig. 2d), indicating that the relative ranking of burial hotspots is robust to this adjustment. Spatially, the adjustment primarily reduced MCBI in the equatorial zone (−30° to 30°), while high-latitude hotspots remained largely unaffected (Fig. 2e), reflecting stronger temperature-driven remineralization in warm tropical waters. Importantly, however, the high sediment accumulation rates characteristic of many mid-to-low-latitude coastlines may counterbalance this thermodynamic penalty: rapid mineral input facilitates the swift burial of detritus below the active oxic zone, shielding organic matter from rapid microbial decay[4].*

Beyond thermal controls, the long-term preservation of macroalgal carbon is also affected by biological consumption by benthic fauna (e.g., grazing by sea urchins[5]) and by physical processes that decouple SAR from net carbon preservation. Although SAR broadly governs the transfer efficiency of organic carbon into deeper sediment layers[6], and net preservation

generally scales positively with SAR, this coupling can break down in hyper-dynamic settings[6]. In high-energy mobile muds (e.g., the Amazon–Guianas mudbelt[7]) and strongly resuspended large-river estuaries[8], where deep sediment mixing repeatedly re-exposes buried material, intense physical reworking subjects organic matter to prolonged oxic degradation, so that high gross SAR does not translate into long-term preservation[6-8]. Future macroalgal blue-carbon models should prioritize integrating spatially resolved benthic food-web dynamics, diagenetic processes, and high-resolution coastal hydrodynamics to move from mapping burial favorability toward quantifying precise carbon fluxes.

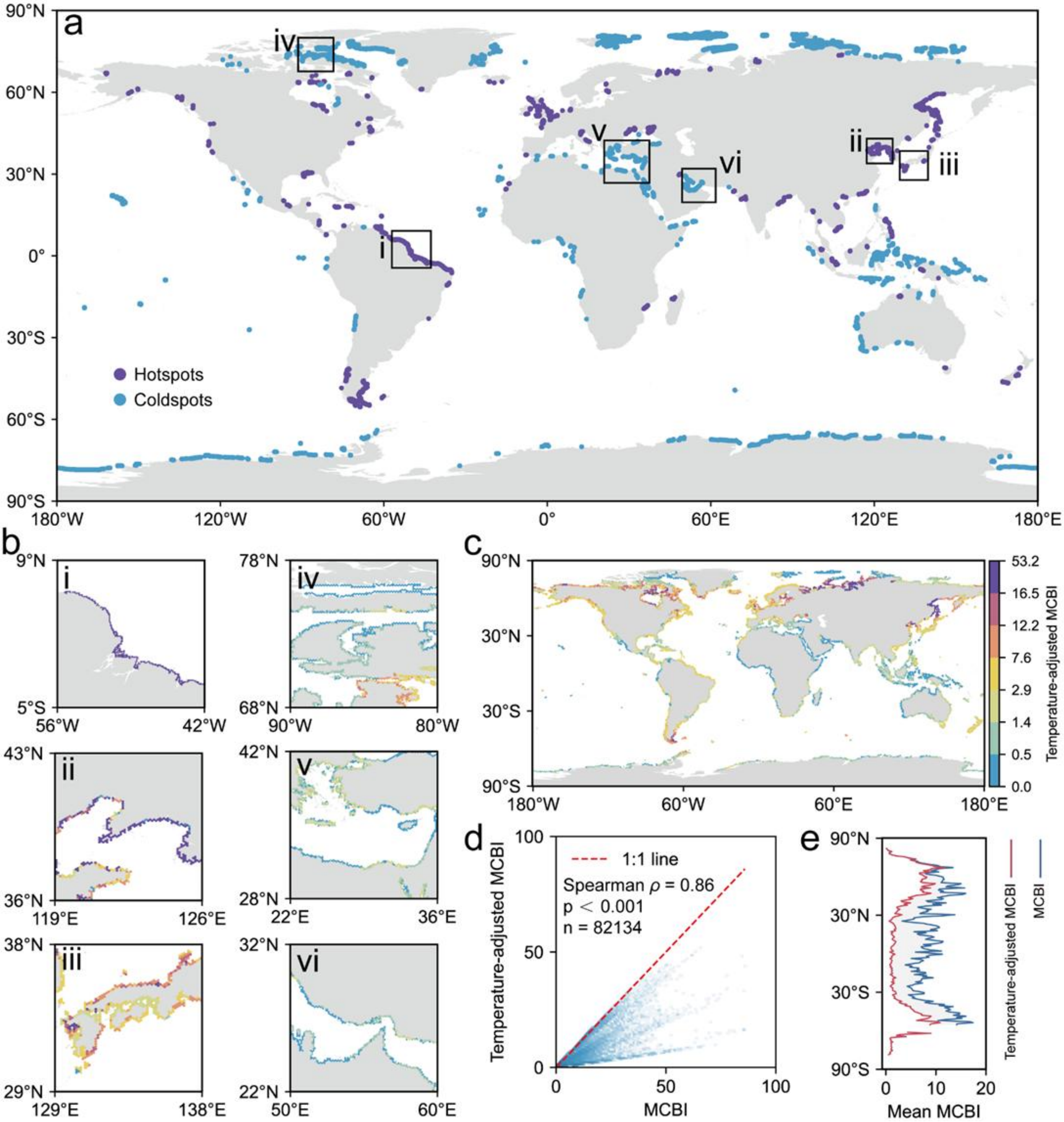

Fig. 2 Biogeochemical coupling and the thermal sensitivity of Macroalgal Carbon Burial Index (MCBI).
a *Hotspots and coldspots of MCBI on a global scale (identified as co-hotspots and co-coldspots of NPP and SAR; 95% confidence level).*
b *Spatially-explicit maps of MCBI in typical hotspot or coldspot areas: (i) the Amazon River estuary, (ii) the Bohai and*

Korea Bays, and (iii) the southwestern Japan as prominent synergistic hotspots; (iv) the Canadian Arctic Archipelago, (v) the Eastern Mediterranean and (vi) the Persian Gulf as representative coldspots.
c *Global distribution of temperature-adjusted MCBI, incorporating a temperature-dependent remineralization factor (Q_{10} = 2.0; see Methods).*
d *Relationship between the original and temperature-adjusted MCBI, assessed by Spearman's rank correlation ($\rho = 0.86$, $p < 0.001$, n = 82,134). The red dashed line denotes the 1:1 relationship.*
e *Latitudinal distribution of the original and temperature-adjusted MCBI (zonal means).*

Comment 4: The naming of the metric is also a bit misleading, as what is quantified is essentially a type of potential burial efficiency (i.e. what fraction of the carbon input may get buried), rather than actual potential burial (i.e. how much carbon may get buried). Since the input is the per-area NPP, the MCBP metric doesn't take into account the absolute input of macroalgal carbon in a given coastline (which depends on the extent of macroalgal habitat AND its productivity). As a result, there are many coasts with high MCBP where the contribution of macroalgae to carbon burial will be negligible because they support very small extents of macroalgae habitat (e.g. Bay of Bengal, Bonaparte Gulf, Duarte et al. 2022). I suggest renaming the metric and explicitly recognizing this limitation in the text. One might argue that macroalgal carbon burial efficiency is what really matters because inputs can always be increased (e.g. farming, restoring seaweeds) or may change in the future, but if that is the case, global maps and predictions are not the right tool to support local-scale interventions.

Response: We are grateful for this comment, which prompted us to state the definition of the metric far more precisely. We agree fully with the reviewer's central point, that the metric does not represent the absolute amount of carbon buried along a given coastline, because it is computed on a per-area basis and does not incorporate the spatial extent of macroalgal habitat.

First, we have renamed the metric from "Macroalgal Carbon Burial Potential (MCBP)" to "Macroalgal Carbon Burial Index (MCBI)", to accurately reflect that the metric quantifies the favorability of coastal environments to entrap and preserve macroalgal organic carbon, rather than the total mass of carbon buried.

Second, we now have explicitly acknowledged this limitation in the text (Lines 140-152): *We emphasize that MCBI is intended to characterize the conditions governing the burial favorability of macroalgal carbon at a given coastline, integrating NPP and SAR on a per-area basis. It therefore represents a relative index of macroalgal carbon burial favorability rather than an estimate of absolute carbon burial, and is best suited to global-scale prioritization of areas where coastal environments are physically conducive to macroalgal carbon sequestration. Because the underlying NPP estimates (Pessarrodona et al.[9]) derive from ecological niche models of potential productivity, high-MCBI regions identify environments favorable for macroalgal carbon burial even where current biomass is locally depleted (e.g., by historical harvesting or overgrazing). Notably, however, because MCBI does not incorporate the actual extent of macroalgal habitat, it should not be interpreted as a measure of the total macroalgal carbon buried at a given location, nor as a substitute for local-scale carbon accounting. As a next step, integrating MCBI with observed or modelled macroalgal distributions (e.g., Duarte et al.[1]) would advance toward the quantification of absolute burial fluxes, representing a promising direction for future work. Moreover, even where sizeable macroalgal stands are currently absent, MCBI can help identify locations where macroalgae aquaculture could yield the strongest carbon-burial benefits beneath the farm.*

Comment 5: The MCBP somewhat overestimates burial as it relies on NPP (the amount of carbon fixed) as opposed to detrital production (the amount of carbon available for transport), but that should be relatively straightforward to address (cf. Filbee-Dexter et al. 2024). Also the approach assumes maximum NPP is the sum of red algal turfs and the canopy, but available evidence suggests that understory and canopy NPP are not additive (Miller et al., 2011). Additionally, these two groups do not always co-occur (at small as well as large spatial scales) and is not clear how the authors dealt with that.

Response: We thank the reviewer for raising these important methodological points regarding the NPP input. We address them sequentially below.

(1) NPP versus detrital production. We fully agree that detrital production, rather than total fixed carbon, represents the fraction available for transport and burial. However, MCBI is a relative, Min-Max normalized index. Since the detrital-to-NPP conversion of Filbee-Dexter et al. (2024) is a globally constant factor (0.71), applying it rescales all NPP values by the same constant, which is eliminated during normalization. Consequently, substituting detrital production for NPP yields identical normalized values and an identical relative spatial ranking of MCBI, and therefore does not affect any of our results, since the metric itself is dimensionless, since the metric itself is dimensionless. We have noted this point in the revised Methods (Lines 458-460).

Lines 458-460: *Because MCBI is Min–Max normalized, any globally uniform rescaling of NPP, such as applying a constant detrital-to-NPP conversion factor (0.71) to represent the fraction of production available for transport[10], leaves the normalized values and the relative spatial ranking unchanged, and is therefore not applied explicitly.*

(2) Additivity of canopy and understory NPP, and (3) co-occurrence. We have clarified our NPP integration approach in the revised Methods to avoid any ambiguity. Our NPP input is derived from the niche-model-based potential NPP of three functional groups—red algal turfs, intertidal brown algae, and subtidal brown algae—from Pessarrodona et al. (2022). For the two brown-algal (canopy) groups we take the maximum rather than the sum, thereby avoiding double-counting within the canopy. We then combine this with the red algal turf value to represent the maximum potential macroalgal productivity under environmentally suitable conditions.

We emphasize that the resulting NPP layer is an upper bound on potential productivity, not an estimate of realized, co-occurring biomass. We acknowledge that shading interactions between canopy and understory groups do occur where these physically co-occur (Miller et al., 2011). MCBI, however, is an index of environmental favorability for burial—characterizing where conditions are conducive to carbon retention rather than how much biomass is currently present—and this potential-based NPP layer is applied consistently to every grid cell. We have made this reasoning explicit, and noted this limitation, in the revised Methods (Lines 429–439):

Lines 429-439: *NPP for each grid cell was derived from the niche-model-based potential NPP of three macroalgal functional groups (red algal turfs, intertidal brown algae, and subtidal brown algae) reported by Pessarrodona et al.[9]. To avoid double-counting productivity within the brown-algal canopy, we took the maximum of the intertidal and subtidal brown-algal values rather than their sum, and combined this with the red algal turf value. To mitigate the influence of extreme model artifacts and ensure conservative estimates, NPP values were capped at the 95th percentile, with all values exceeding this threshold reassigned to the 95th-percentile value[10]. The resulting quantity represents the maximum potential macroalgal productivity attainable under environmentally suitable conditions, rather than an estimate of realized, co-occurring biomass; it therefore does not account for shading interactions between canopy and understory groups where these physically co-occur[11]. This potential-based definition is consistent with the purpose of MCBI as a relative index of burial favorability. Separate analyses for individual functional groups are provided in Supplementary Results 3.*

Comment 6: Predictive maps are only as good as the data that underpins them. The largest data contributor to the SAR dataset (ca. 30% of records) comes from a similar compilation by Song et al. 2022. SARs were not the focus of Song et al. 2022 and so were often extrapolated based on some assumptions. For instance, just looking at the first set of data from their dataset (data from A, Kanitha Srisuksawad et al, 1997 from the Gulf of Thailand), I see that SARs of different locations are often exactly the same and only varied from 7.7-12. Yet if you go to the original Kanitha study the actual SARs vary from 18-70 and are all different. It is not clear form Song et al. 2022 why that is but the fact that this is literally the first records that I glanced over makes me suspicious about all the data underlying your SAR model.

Response: We thank the reviewer for this careful observation. We investigated this issue through our co-author Prof. Faming Wang, who is a coauthor of the Song et al. (2022) and familiar with its data compilation. We learned that the SAR values in that database were not direct field measurements from the cited original studies, but were instead derived from the interpolation of SAR values from neighboring coastal wetlands. This explains the discrepancies noted by the reviewer between the Song et al. database entries and the original source values (e.g., Kanitha Srisuksawad et al., 1997).

Given these findings and the associated constraints on the reliability of the Song-derived records, we have taken the conservative approach of removing all records originating from the Song et al. (2022) compilation from our SAR dataset and re-trained the model on the remaining data (n = 1,617). All results reported in the revised manuscript are therefore based on this Song-free dataset. The spatial coverage of the remaining data remains broadly representative across the global coastal ocean, and the re trained model shows no significant degradation in predictive performance ($R^2 = 0.64$, Supplementary Fig. 1).

The remaining records in our dataset are drawn directly from primary field studies with reported measurement methods, and we have verified their consistency during compilation.

We believe this revision substantially strengthens the robustness of our predictive mapping. We are grateful to the reviewer for prompting this important data quality check.

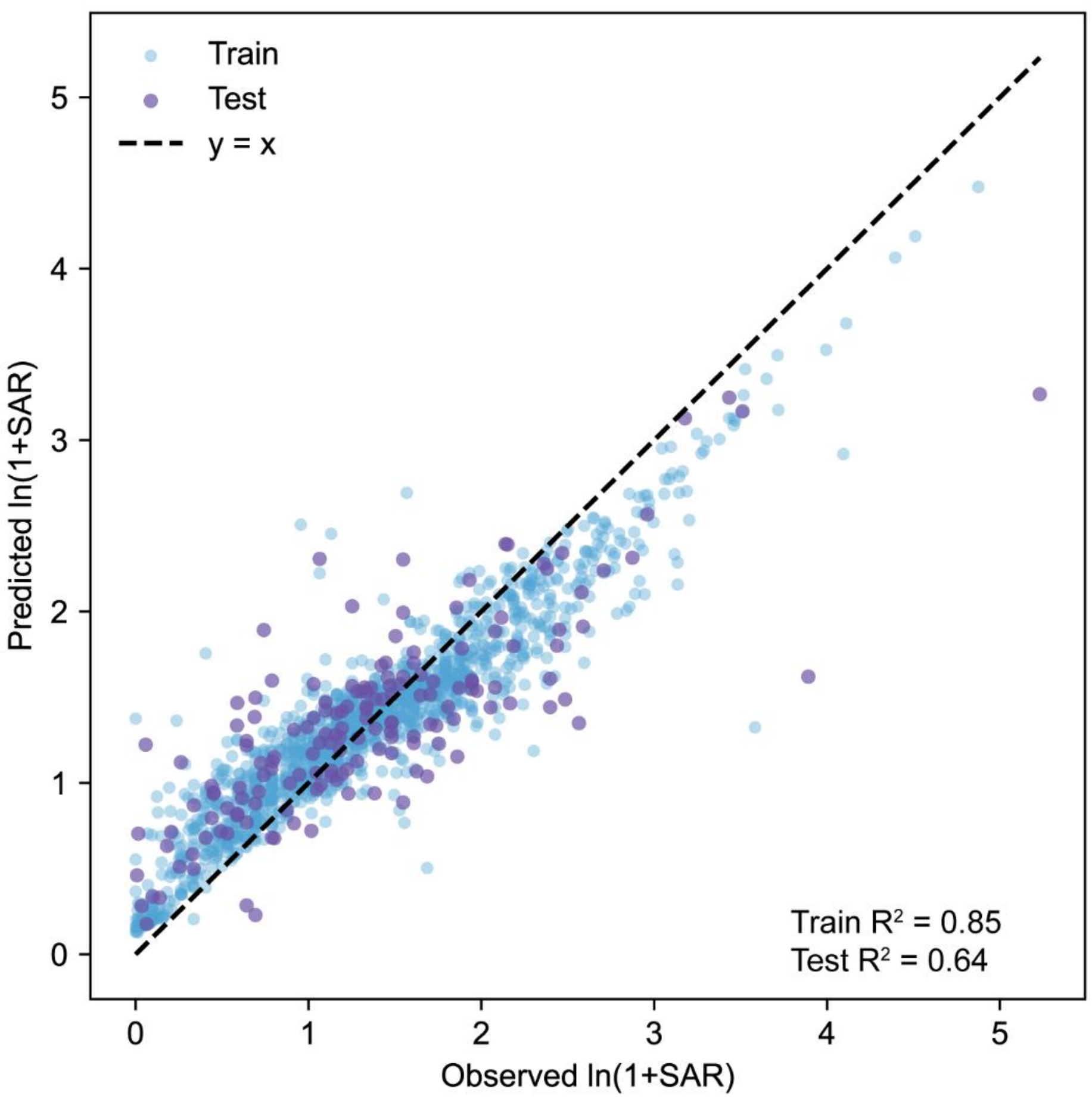

Supplementary Fig. 1 The relationship between observed ln(1 + SAR) and predicted ln(1 + SAR).

Comment 7: Another important issue is that your SAR data contains records from very different depositional environments (e.g. tidal wetlands and continental shelves). The authors argue that this doesn't matter because they are ultimately able to parametrize SAR based on physical drivers such as hydrodynamics and sediment supply. However, this is problematic

because the global products you used to predict SAR (cf. Supplementary Table 2) are unlikely to work equally well for open ocean environments and coastal estuarine environments. For example, NPP estimates in coastal areas are notoriously error-prone (Saba et al., 2011), and so are the ETOPO water-depths in shallow estuarine settings (two of your top predictors), which have 500 m resolution.

Response: We thank the reviewer for raising this important point about the reliability of global products in coastal environments. We have taken this concern seriously and have made substantial revisions to address it.

First, regarding NPP: in the revised model, we have removed phytoplankton-based NPP (Primary productivity) as a predictor. This decision was based on two considerations: (i) the reviewer correctly noted the high uncertainty in coastal NPP estimates (Saba et al., 2011), and (ii) in our original model, removing Primary productivity does not degrade model accuracy (R^2 before vs. after removal: 0.64 vs. 0.64), while improving the model's parsimony and reducing reliance on a high uncertainty input.

Second, regarding water depth: we have replaced ETOPO with the GEBCO 2026 bathymetric grid, the latest and most authoritative publicly available bathymetry, produced under the joint auspices of the IHO and IOC (UNESCO) through the Nippon Foundation–GEBCO Seabed 2030 Project. Unlike ETOPO, which relies substantially on satellite-derived predicted depths, GEBCO integrates an extensive and continually growing body of measured bathymetric data (multibeam, single-beam, and crowdsourced soundings), and incorporates higher-resolution regional compilations in many coastal areas. It is accompanied by a source-type identifier grid documenting, cell by cell, whether each depth value is based on measured or predicted data. This substantially improves the reliability of water-depth values in nearshore and shelf settings compared with ETOPO. Following this change, water depth emerged as one of the two dominant predictors in the model, alongside total suspended matter (Supplementary Methods 1, Supplementary Fig. 1, Supplementary Fig. 3).

We nonetheless acknowledge that, as the reviewer notes, global products inevitably carry greater uncertainty in coastal and estuarine environments than in open-ocean settings. We have added this as an explicit limitation in the revised manuscript (Lines 423-425), and we discuss the associated caveats of applying globally-trained models to heterogeneous coastal environments.

Third, beyond these two predictors, we re-examined the remaining environmental drivers used in the model (Supplementary Table 2). In particular, we have replaced our tidal parameters with those derived from the FES2022b atlas, which includes a coastally extrapolated solution developed specifically to resolve nearshore cells that global tidal products typically leave unconstrained. While the remaining drivers are global-scale products and therefore inevitably carry some uncertainty in coastal settings, they represent the best available global characterizations of their respective physical processes. We have retained them in the revised model, but now explicitly acknowledge the general limitations of global products in nearshore environments.

We believe these revisions substantially strengthen the robustness and transparency of our SAR predictions in coastal environments, and we are grateful to the reviewer for prompting these important improvements.

Lines 423-425: *This assumption necessarily has limits: as with all globally-trained models, the environmental products used here carry greater uncertainty in nearshore than in open-ocean environments, which may introduce local biases in coastal SAR predictions.*

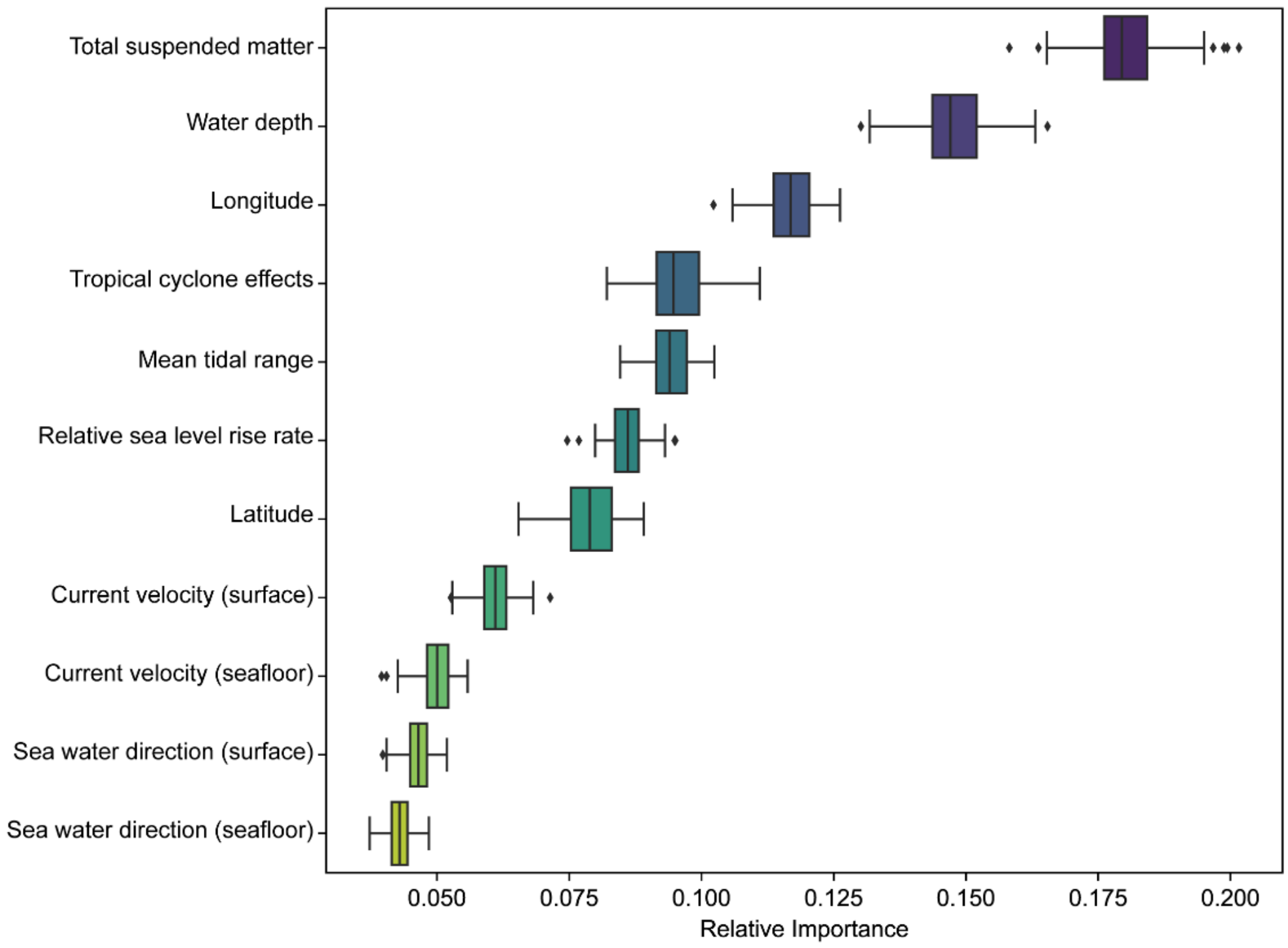

Supplementary Fig. 3 Feature importance of predictors for coastal SAR across 100 random iterations.

Comment 8: Importantly, the strong geographic clustering of depositional environments within the dataset (e.g. most Norwegian data originating from shelf sediments, whereas Western Australian data are largely derived from wetlands or estuarine environments) appears to be an important driver of the model outputs. For example, despite broadly similar NPP values and fjord-dominated coastlines, southern Patagonia exhibits approximately twice the MCBP of Atlantic Norway. Based on the data provided, this difference appears to arise because the Patagonian SAR observations are derived almost exclusively from fjord environments, which are characterized by high sediment accumulation rates (e.g. Figure 1b), whereas the Norwegian dataset contains substantially more continental shelf samples with comparatively low SARs. Consequently, the model may attribute higher SARs to Patagonia primarily because of the geographical location (cf. Fig. S4), even though we know Norwegian coastlines have similar carbon burial rates than Patagonian ones (Smith et al., 2015). I recognize that global assessments inevitably contain some degree of geographical bias; however, validation against existing regional knowledge and observations is precisely what gives these products confidence and utility. At present, the spatial patterns of SAR and MCBP do not seem to align with our current understanding of burial potential, as also noted by another reviewer.

Response: We thank the reviewer for this penetrating and constructive comment. We agree that global assessments inevitably carry geographical bias, and we have taken several steps to examine this issue directly and to report the limitations transparently.

We note that our compiled dataset contains only 2 SAR records from Patagonia, compared with 158 from Norway. Given this very limited local sampling, the predicted SAR for Patagonia relies substantially on the model's globally-learned relationships between SAR and environmental predictors, rather than on dense local observations. While we cannot fully exclude a contribution of geographic location to the prediction, the scarcity of Patagonian records suggests that the regional contrast highlighted by the reviewer is unlikely to be driven primarily by locally clustered fjord observations.

Indeed, Smith et al. (2015) showed that the two regions have comparable organic carbon burial rates (i.e., organic carbon accumulation rates, OC AR). Crucially, however, Smith et al. (2015; Fig. 2) also showed that the OC content of the sediments is about three times lower in Chilean Patagonia (~1.8%) than in NW Europe (Atlantic Norway, ~5.6%).

Therefore, if both regions have similar amount sediment carbon burial per unit area each year but Patagonian sediment have only one-third as much carbon, then Patagonia must accumulate substantially more sediment to bury the same amount of carbon. In other words, the same OC AR combined with much lower carbon content implies a higher SAR in Patagonia—precisely the pattern our model predicts, rather than the reverse.

On the broader question of geographic transferability. To assess directly how well the model predicts geographically unseen regions, we performed spatial block cross-validation (leave-one-block-out over a 5° grid; according to Meyer & Pebesma, 2022). As expected for spatially autocorrelated environmental data, the absolute predictive skill decreased relative to random cross-validation (R^2 from 0.59 to 0.30), confirming that extrapolation of absolute SAR values across regions is inherently limited—consistent with the reviewer's expectation of geographic bias in global products. Nonetheless, the relative spatial ranking of SAR remained moderately preserved across held-out blocks (Spearman $\rho = 0.51$), indicating that the model captures transferable relative gradients rather than purely location-specific patterns. Because MCBI is a relative index—designed to identify the relative ranking of burial hotspots rather than absolute SAR values—this relative transferability supports its intended application.

In line with the reviewer's recommendation, we now explicitly discuss these constraints in the revised manuscript (Lines 154-170), including the reduced reliability of predictions in under-sampled or environmentally atypical regions and the general limitations of machine-learning-based global extrapolation (according to Meyer & Pebesma, 2022). We further note that, in the revised model, the importance of geographic coordinates decreased substantially relative to the previous version (Supplementary Fig. 3), with water depth and tropical cyclone effects emerging as the dominant predictors—indicating that predictions are governed primarily by physical drivers rather than by geographic location per se.

Lines 154-170: *As with all machine-learning-based global assessments, our predictions are subject to geographical bias arising from the uneven spatial distribution of available observations, which reflects historical differences in research effort rather than underlying environmental variability[12]. Overall, our SAR predictions are stable across most temperate and tropical coastlines (98.5% of coastlines with a coefficient of variation below 0.3 across 100 bootstrap iterations; Supplementary Fig. 4), while larger uncertainties persist in under-sampled regions such as the polar coastlines. To further evaluate where predictions are well constrained by data versus where they rely on extrapolation, we performed spatial block cross-validation and computed two complementary coverage metrics, the feature-space distance (mean Euclidean distance to the five nearest training observations in the standardized predictor space, excluding geographic coordinates) and the geographical distance to the nearest observations (Supplementary Methods 2)[12]. As expected for spatially autocorrelated data, the block cross-validation showed that absolute predictive skill decreased relative to random cross-validation, although the relative spatial ranking of predictions remained moderately preserved (Supplementary Methods 2). The two coverage metrics further reveal regions where apparent precision may be misleading. Areas with small feature-space distance but large geographical distance (e.g., the west coast of Africa) exemplify the decoupling between environmental representativeness and local data availability: predictions there may appear precise (low coefficient of variation) yet lack local validation, and should be interpreted with caution irrespective of their apparent uncertainty. Together, these analyses (Supplementary Fig. 4–6) delineate where the global predictions are robust and where they should be treated as extrapolation.*

Comment 9: In summary, I find this to be a conceptually strong and potentially important contribution. However, the usefulness of the resulting maps ultimately depends on the robustness of their validation. The emergence of seemingly implausible hotspots raises concerns about the predictive performance of the model and suggests that one or more important controlling variables may be missing. As a result, the current maps are difficult to interpret with confidence and their practical utility remains limited.

Response: We are grateful for recognizing this as a conceptually strong and potentially important contribution, and we take the concerns about validation and interpretability very seriously. In this revision, we have substantially strengthened the manuscript in three interconnected ways.

First, regarding validation and robustness. We have added spatial block cross-validation to directly assess the model's ability to predict geographically unseen regions, and complementary geographical and feature-space coverage analyses to characterize where predictions are well-constrained by data versus where they rely on extrapolation (see our response to Comment 6, 10).

Second, regarding the seemingly implausible hotspots. We have systematically examined these cases. Several have been resolved in the revised model: following the removal of the Song et al. (2022) records, regions such as the Yellow Sea are no longer identified as high-MCBI areas (see our response to Comment 3). For other cases, such as the high values in the Patagonian fjords, we show that the pattern is in fact consistent with independent observations (see our response to Comment 8). We have also incorporated a temperature-dependent remineralization factor as a sensitivity analysis to represent one of the key downstream processes affecting the fate of buried carbon. The strong correlation between the temperature-adjusted and original metrics (Spearman $\rho = 0.86$, $p < 0.001$) indicates that the hotspot ranking is robust to this important biological modifier.

Third, regarding interpretability and scope. To address the concern that the original naming implied greater predictive certainty than warranted, we have renamed the metric to Macroalgal Carbon Burial Index (MCBI), and now explicitly define it as a relative index of burial favorability intended for global-scale prioritization rather than absolute quantification, while clearly stating its limitations (see our response to Comments 4 and 5).

Finally, regarding the reviewer’s concern that one or more important controlling variables may be missing: we fully acknowledge this possibility. In the revision, we have taken a dual approach. Where globally available data allowed, we have incorporated additional constraints—specifically, the temperature-dependent remineralization sensitivity analysis. For other potentially important processes—such as sediment mixing (Song et al., 2022), burial/transfer efficiency (Bradley et al., 2022), and benthic faunal grazing—we found that their inclusion would introduce substantial redundancy (because they are heavily driven by SAR, already a core component of our metric) or is currently precluded by the lack of globally consistent, spatially explicit datasets. We now explicitly discuss these omissions as clear limitations and priorities for future model development in the revised Discussion (see our response to Comment 3).

These revisions collectively address the reviewer’s concerns and substantially improve the robustness, interpretability, and practical utility of the maps. We are grateful to the reviewer for the constructive and rigorous feedback, which has significantly strengthened the manuscript.

Specific comments

Comment 10: Ln. 153. Your uncertainty is a function of variability within areas that you have sampled. But several under sampled areas (e.g. entire west coast of Africa) have very small variation just because you don’t have any data

Response: We thank the reviewer for this perceptive observation. The bootstrap uncertainty quantifies the precision (consistency) of the model's predictions under data resampling, that is, how stable the predictions are. We fully agree that low uncertainty in under-sampled regions such as the west coast of Africa should not be interpreted as high predictive reliability.

To better understand this behavior, we further examined two complementary coverage metrics for each prediction (Supplementary Fig. 5 and 6). For every predicted grid cell, we computed (i) the geographical distance to the nearest

training observations (mean great-circle distance to the k = 5 nearest sites), which reflects how far a prediction is from any local data; and (ii) the feature-space distance to the nearest training observations (mean Euclidean distance to the k = 5 nearest sites in the standardized environmental-predictor space, excluding geographic coordinates), which reflects whether the environmental conditions of a prediction are represented within the training data. A small feature-space distance indicates that the model has encountered similar environmental settings during training, whereas a large geographical distance indicates the absence of nearby observational constraint. This applicability-domain framework follows Meyer & Pebesma (2022). This analysis clarifies the underlying cause. The west coast of Africa exhibits a relatively small feature-space distance, indicating that its environmental conditions fall within the well-sampled range of the predictor space; the model has therefore encountered similar environmental settings elsewhere and produces consistent (hence low-uncertainty) predictions for this type of environment. At the same time, this region shows a large geographical distance to the nearest observations, confirming that it is genuinely data-sparse and lacks local observational constraint.

This decoupling—consistent predictions supported by environmental similarity, yet no local data for validation—illustrates precisely the limitation the reviewer notes. We emphasize that such data gaps in regions like the west coast of Africa are a widespread feature of global marine sediment datasets and represent a broader, well-recognized sampling gap in the field. We now present these coverage metrics alongside the bootstrap uncertainty and explicitly discuss this limitation in the revised manuscript (Lines 154-170, see our response to Comment 8), noting that predictions in geographically under-sampled regions should be interpreted with appropriate caution regardless of their apparent uncertainty.

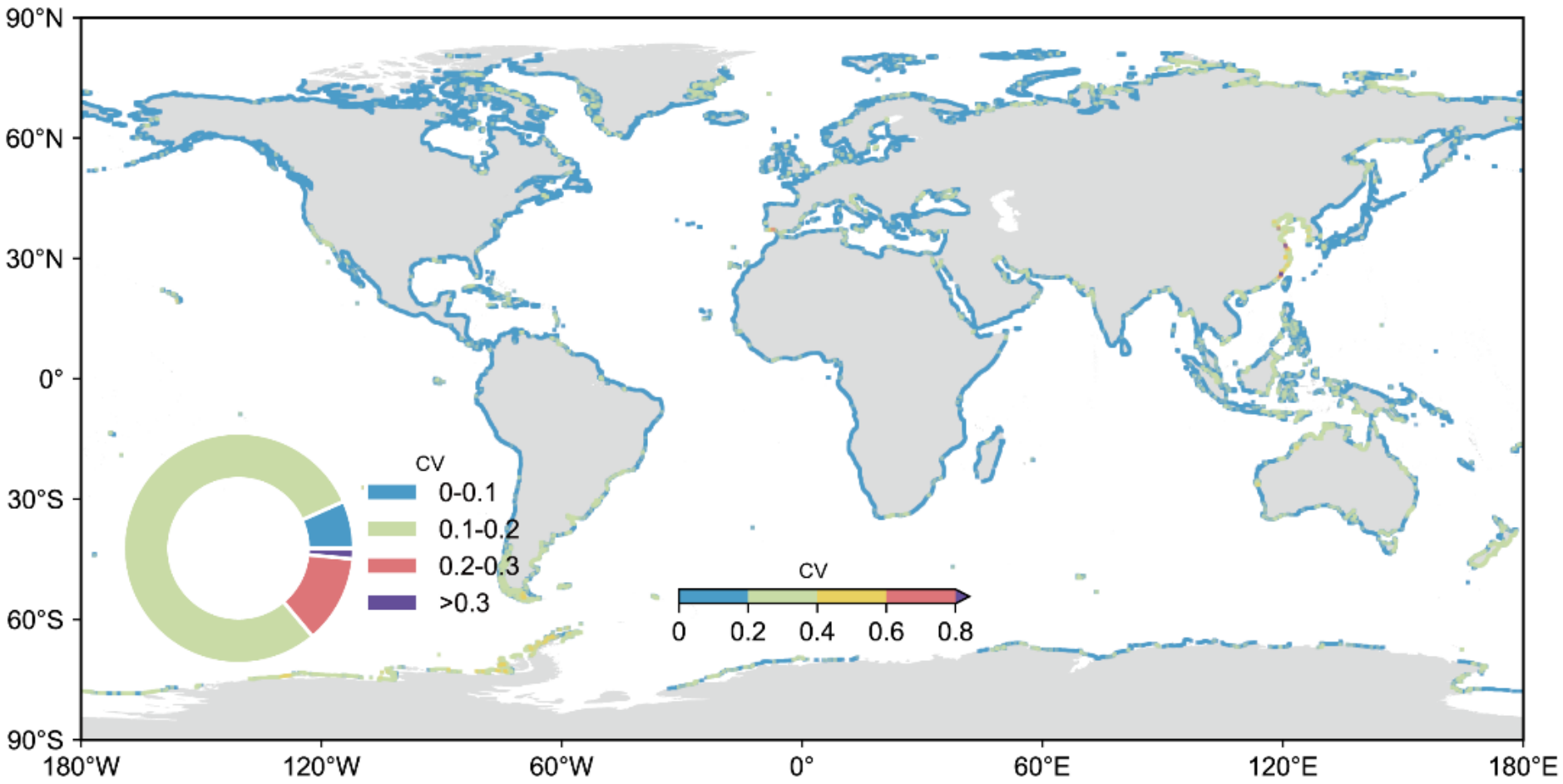

Supplementary Fig. 4. Spatial uncertainty of the global coastal sediment accumulation rate (SAR) predictions, expressed as the coefficient of variation (CV) across 100 bootstrap iterations. The inset shows the frequency distribution of CV values across all predicted coastal grid cells.

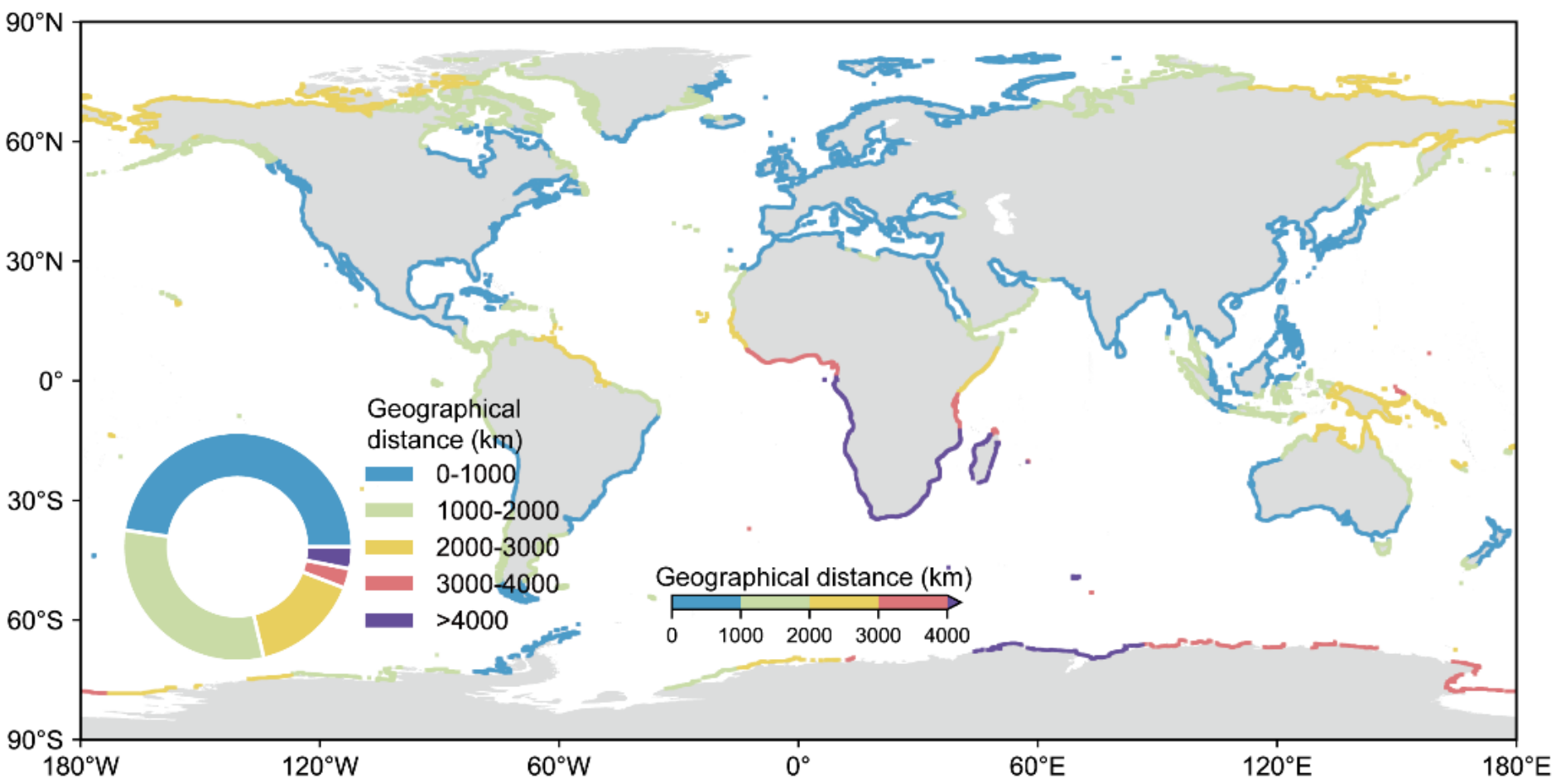

Supplementary Fig. 5. Spatial geographical distance of the global coastal Sediment Accumulation Rate (SAR) predictions. The inset shows the frequency distribution of geographical distance across all predicted coastal grid cells.

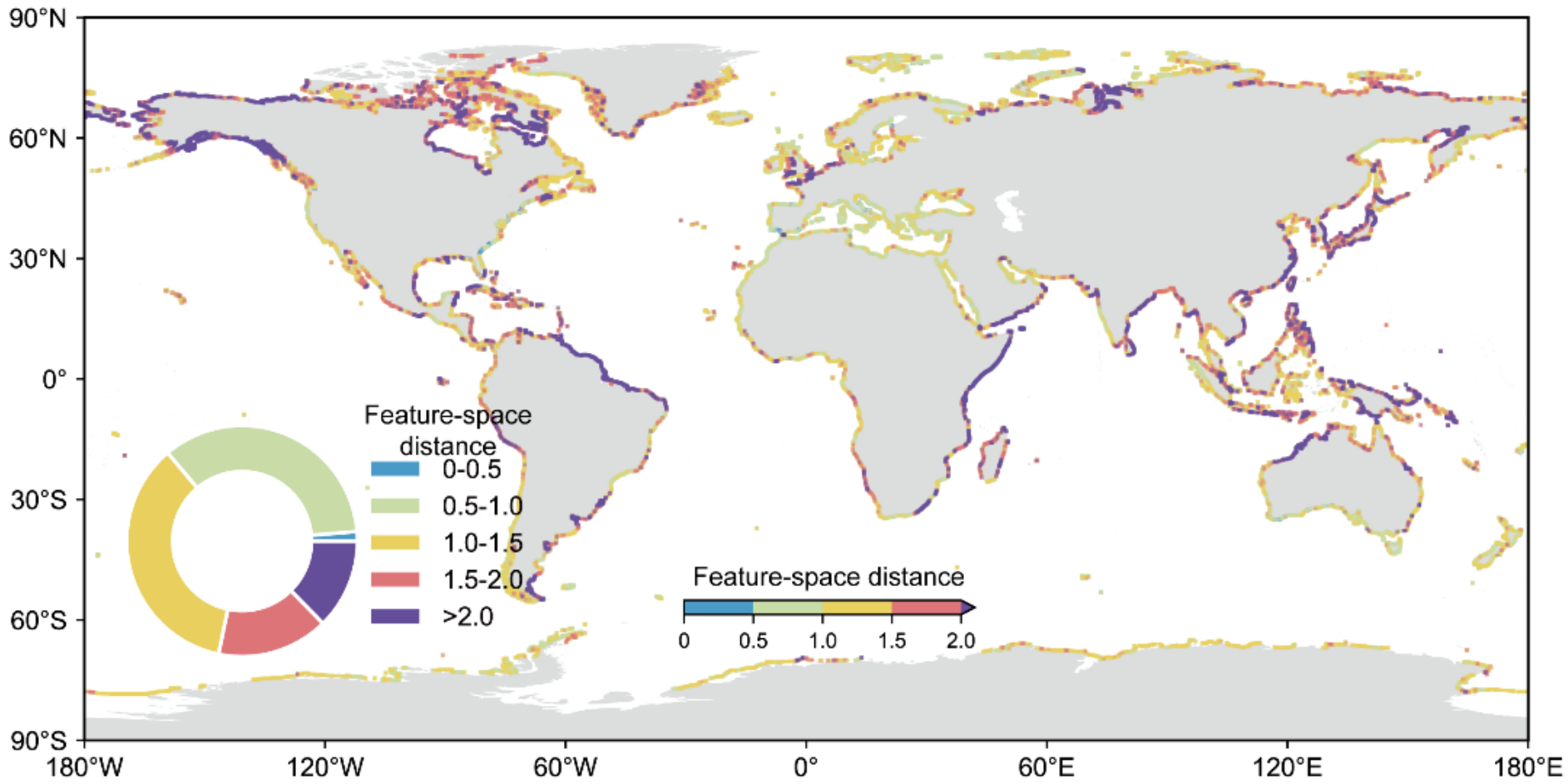

Supplementary Fig. 6. Spatial Feature-space distance of the global coastal Sediment Accumulation Rate (SAR) predictions. The inset shows the frequency distribution of Feature-space distance across all predicted coastal grid cells.

Ln. 211. These are not really comparable because Filbee-Dexter accounted for the areal extent, see my comment above.

Response: We thank the reviewer for this point and agree that a direct comparison between MCBI and the estimates of Filbee-Dexter et al. (2024) is not appropriate.

The two metrics differ fundamentally in what they represent. Although both are ultimately derived from niche-model-based species distributions, Filbee-Dexter et al. constrain their estimates to the observed areal extent of seaweed forests and quantify the realized flux of seaweed carbon exported across the shelf. In contrast, MCBI is computed across all coastlines as a relative index of burial favorability based on per-area productivity, and does not incorporate the actual distribution or extent of macroalgal habitat (see our response to Comment 2).

For these reasons, we have removed this comparison and added a discussion in the revised manuscript.

Comment 11: Ln. 370. This doesn't match with Figure 1 b

Response: We thank the reviewer for catching this inconsistency. In the previous version, the text and Fig. 1b referred to different record counts (the raw total versus the subset used for modeling), which caused the mismatch. In the current revision this no longer applies. The dataset comprises 1,617 records, all of which were used for model training and are shown in Fig. 1b. We have verified that the text and figure are now fully consistent throughout the manuscript.

Comment 12: Ln. 390. It seems like you took the same approach as Song et al. 2022, whose data came mostly from continental shelves. Given the large amount of data from tidal wetlands that you have included in your compilation, I wonder how well you can resolve these parameters using global products.

Response: We thank the reviewer for this point. We note that tidal wetlands constitute a substantial and intentional component of our compilation, because a considerable fraction of macroalgal carbon burial occurs in tidal-wetland and other shallow coastal settings. We would also note that the Song et al. (2022) compilation likewise includes a considerable proportion of non-shelf environments—approximately 38% of their sites are in intertidal flats, wetlands, and the deep ocean—so the environmental composition of the two datasets is in fact broadly comparable in this respect.

We agree that global environmental products are optimized primarily for open-shelf environments and are inherently more uncertain in resolving parameters within fine-scale, very shallow tidal-wetland settings. To mitigate this, we have replaced ETOPO with the higher-quality GEBCO bathymetry, which better resolves nearshore depths, replaced our tidal parameters with the FES2022b atlas, which provides a coastally extrapolated solution developed specifically for nearshore cells, and removed satellite-derived PP. We nonetheless acknowledge that residual uncertainty remains for tidal-wetland environments, and we have added this explicitly as a limitation in the revised manuscript (Lines 423-425; detailed in our response to Comment 7).

Comment 13: Ln. 389. Was there correlation between your variables and how did you deal with that?

Response: Prior to model development, we assessed multicollinearity among all candidate predictors using the Variance Inflation Factor (VIF). This analysis identified strong collinearity within a group of tidal variables (mean tidal range, mean high water spring, and mean high water neap; VIF>10). To eliminate this redundancy, we retained mean tidal range as a single representative variable and excluded the others. After this screening, all predictors used in the final model exhibited low collinearity (VIF<2). This procedure is described in the revised Supplementary Methods 1.

Comment 14: Ln. 394. It would be good to include a few key information in your summary table (spatial resolution of the product, timescale, global coverage). Also for primary productivity I assume you mean phytoplankton NPP?

Response: We have added the requested information (spatial resolution, timescale, and global coverage) for each predictor in the revised Supplementary Table 3.

Regarding primary productivity: the reviewer is correct that the phytoplankton-based primary productivity was a satellite-derived product. As noted in our response to Comment 7, this predictor has now been removed from the revised model.

Comment 15: Ln. 418. Is not clear if you used intertidal and subtidal brown algal forests and when?

Response: For each grid cell, we took the maximum of the intertidal and subtidal brown algal NPP values (rather than their sum, to avoid double-counting within the canopy) and combined this with red algal turf NPP to obtain the total macroalgal NPP. Full details of this integration approach are provided in our response to Comment 3.

Reference

1. Filbee-Dexter, K. et al. Kelp carbon sink potential decreases with warming due to accelerating decomposition. *PLOS Biol.* **20**, e3001702 (2022).
2. Davidson, E. A. & Janssens, I. A. Temperature sensitivity of soil carbon decomposition and feedbacks to climate change. *Nature* **440**, 165–173 (2006).
3. Arndt, S. et al. Quantifying the degradation of organic matter in marine sediments: A review and synthesis. *Earth Sci. Rev.* **123**, 53–86 (2013).
4. Hedges, J. I. & Keil, R. G. Sedimentary organic matter preservation: an assessment and speculative synthesis. *Mar. Chem.* **49**, 81–115 (1995).
5. Filbee-Dexter, K. & Scheibling, R. E. Detrital kelp subsidy supports high reproductive condition of deep-living sea urchins in a sedimentary basin. *Aquat. Biol.* **23**, 71–86 (2014).
6. Bradley, J.A., Hülse, D., LaRowe, D.E. & Arndt, S. Transfer efficiency of organic carbon in marine sediments. *Nat. Commun.* **13**, 7297 (2022).
7. Aller, R. C. & Blair, N. E. Carbon remineralization in the Amazon–Guianas tropical mobile mudbelt: A sedimentary incinerator. *Cont. Shelf Res.* 26, 2241–2259 (2006).
8. Song, S. et al. A global assessment of the mixed layer in coastal sediments and implications for carbon storage. *Nat. Commun.* **13**, 4903 (2022).
9. Pessarrodona, A. et al. Global seaweed productivity. *Sci. Adv.* **8**, eabn2465 (2022).
10. Filbee-Dexter, K. et al. Carbon export from seaweed forests to deep ocean sinks. *Nat. Geosci.* **17**, 552–559 (2024).
11. Miller, R.J., Reed, D.C. & Brzezinski, M.A. Partitioning of primary production among giant kelp (*Macrocystis pyrifera*), understory macroalgae, and phytoplankton on a temperate reef. *Limnol. Oceanogr.* **56**, 119–132 (2011).
12. Meyer, H. & Pebesma, E. Machine learning-based global maps of ecological variables and the challenge of assessing them. *Nat. Commun.* **13**, 2208 (2022).

REVIEWER COMMENTS

Responses to comments from Reviewer #4:

I recognize this has been a long process for the authors and welcome their openness to take constructive feedback. I do have an outstanding concern that will leave the editor to decide on.

I don't really agree that Bradley et al. parametrize transfer efficiency "largely based on SAR and water depth". There is quite an effort to characterize post-depositional organic matter degradation dynamics in their metric (a key weakness of the MCBI), and I personally don't buy the redundancy argument made by the authors in this revision. Incorporating transfer efficiency (e.g., Fig. 3a; the 100-year burial efficiency appears particularly relevant) into the MCBI metric would make it a much stronger and more valuable indicator - and that is coming from someone that is not even on that paper! Burial efficiency is already normalized on a 0–100 scale, and so incorporating it should not introduce any substantial numerical complications.

Response: We are very grateful for this thoughtful recommendation, and for the patience and commitment of the reviewer in improving the paper. We take the reviewer's point on the redundancy argument, and on reflection we agree it was not well founded: Bradley et al.'s transfer efficiency is derived from a spatially resolved reaction-transport model that mechanistically represents the progressive degradation of organic carbon during burial, and thus characterizes the preservation of buried carbon far more rigorously than a simple environmental proxy could. We thank the reviewer for pressing us on this point, which has led to a stronger manuscript.

Action: We have incorporated the 100-year organic-carbon transfer efficiency of Bradley et al. (2022) into our framework. Following the reviewer's suggestion, we weighted the MCBI by the 100-year burial efficiency to construct a centennial-scale MCBI, which represents not only the physical favorability of a coastline for burying macroalgal carbon but also the fraction of that carbon expected to survive a century of post-depositional degradation (Fig. 1c; Lines 154–165; Supplementary Methods 4). As the reviewer anticipated, because it is already expressed on a normalized scale, its incorporation introduced no numerical complications.

The centennial-scale index remains strongly rank-correlated with the base MCBI (Spearman $\rho = 0.76$; Supplementary Fig. 8), confirming that our principal spatial conclusions are robust, while additionally resolving where buried carbon is more likely to be retained over centennial timescales. Coastlines that rank highly under both metrics, such as the Okhotsk–Sakhalin region, the eastern Philippines and the southern Patagonian fjords, emerge as the settings where favorable burial conditions coincide with efficient long-term preservation. We note that the transfer-efficiency values carry appreciable uncertainty in nearshore and shelf environments, and we therefore present this as an exploratory step toward longer-term carbon accounting; the base MCBI remains the basis for our downstream analyses. As the reviewer expected, incorporating burial efficiency has made the framework a more complete and more valuable indicator of long-term macroalgal carbon burial.

The text now reads:

Lines 154-165: *Beyond spatial extent, the long-term fate of buried carbon is governed by post-depositional preservation. To explore this dimension, we developed a centennial-scale MCBI by weighting MCBI with the 100-year organic-carbon transfer efficiency of Bradley et al.[1], which estimates the fraction of deposited carbon that survives a century of degradation within the sediment (Supplementary methods 4, Fig. 1c). The resulting index preserves the principal spatial patterns of MCBI while additionally reflecting where buried carbon is more likely to be retained over centennial timescales (Supplementary Fig. 8). Coastlines that rank highly under both metrics, including the Okhotsk–Sakhalin region, the eastern Philippines, and the southern Patagonian fjords, represent settings where favorable burial conditions coincide with efficient long-term preservation, and thus constitute the most robust priorities for macroalgal carbon management (Fig. 1c). We regard this as an exploratory step toward longer-term carbon accounting rather than a definitive preservation estimate, as the underlying transfer-efficiency values derive from a global reaction-transport model that carries substantial uncertainty in nearshore and shelf settings. Nonetheless, coupling burial favorability with preservation efficiency illustrates a path toward resolving not only where macroalgal carbon is buried, but how durably it is stored.*

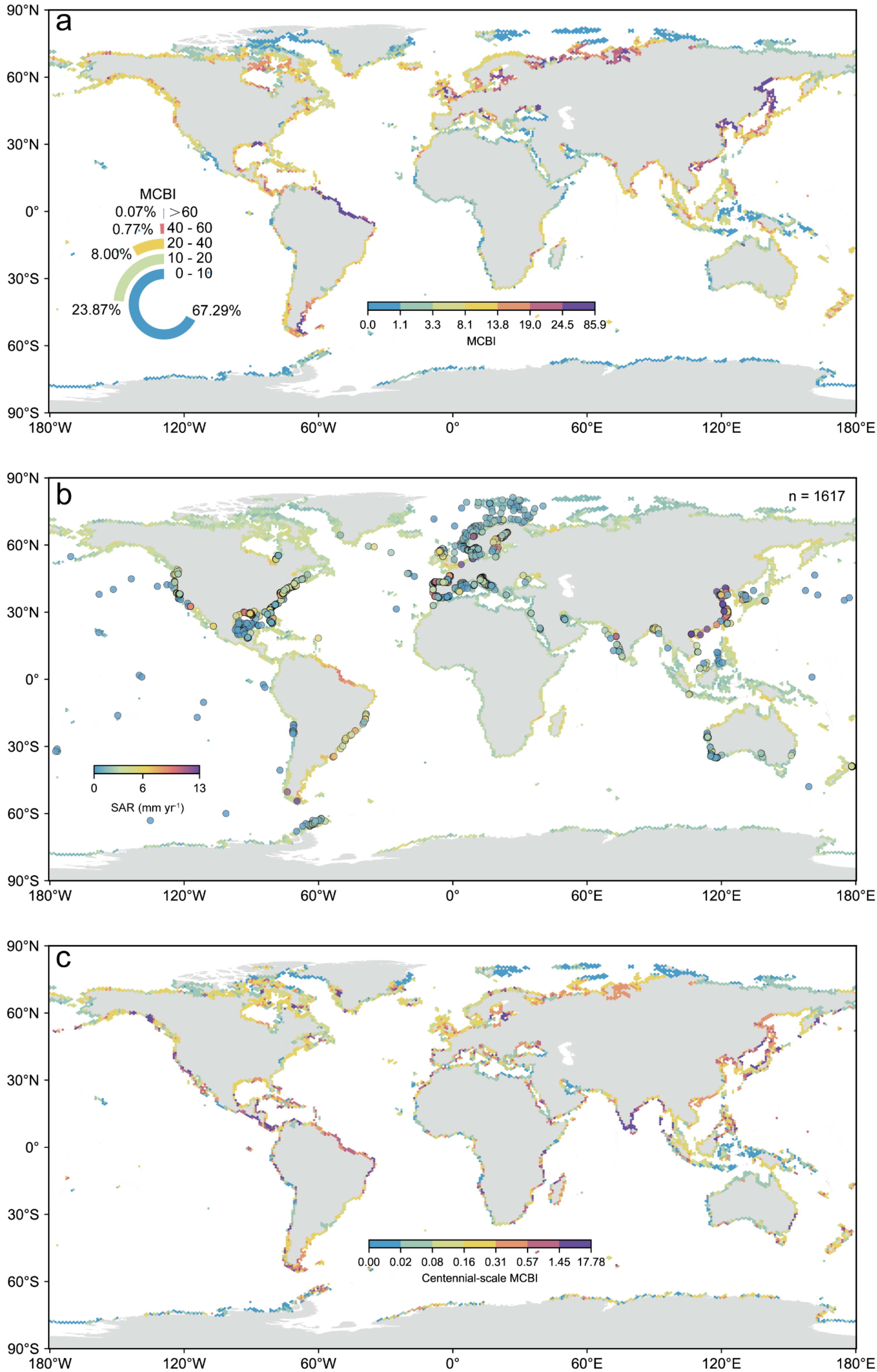

a
MCBI
0.07%
>60
0.77%
40 - 60
8.00%
20 - 40
10 - 20
0 - 10
23.87%
67.29%
0.0
1.1
3.3
8.1
13.8
19.0
24.5
85.9
MCBI
b
n = 1617
0
6
13
SAR (mm yr⁻¹)
c
0.00
0.02
0.08
0.16
0.31
0.57
1.45
17.78
Centennial-scale MCBI
90°N
60°N
30°N
0°
30°S
60°S
90°S
180°W
120°W
60°W
0°
60°E
120°E
180°E

Fig. 1 Global assessment of Macroalgal Carbon Burial Index (MCBI) and its physical foundation.

***a** Spatial distribution of MCBI across global coastlines, and the inset displays the frequency distribution of MCBI.*

***b** Global high-resolution reconstruction of marine Sediment Accumulation Rate (SAR, mm yr^{-1}) derived from a Gradient Boosting Regression (GBR) model, and dots indicate the locations of empirical SAR measurements (n=1,617) compiled to constrain the model.*

***c** Spatial distribution of the centennial-scale MCBI, obtained by weighting MCBI with the 100-year organic-carbon transfer efficiency of Bradley et al. (2022)[1].*

Maps were sourced from Natural Earth (https://www.naturalearthdata.com/).

Supplementary Methods 4: Centennial-scale MCBI

To extend the MCBI toward longer-term carbon storage, we incorporated the 100-year organic-carbon transfer efficiency (T_{eff}) of Bradley et al.[1], which quantifies the fraction of organic carbon deposited at the sediment–water interface that survives 100 years (0.1 ka) of post-depositional degradation (their $T_{eff,age}$, SWI→0.1 ka). This transfer-efficiency field is derived from a spatially resolved global reaction-transport model in which organic-carbon degradation is represented mechanistically.

The global T_{eff} field, distributed at 0.25° resolution, was sampled to our 5-arcminute coastal grid by nearest-neighbour assignment. For each coastal grid cell we defined the centennial-scale MCBI as:

$$\text{Centennial-scale MCBI} = \text{MCBI} \times (T_{eff} / 100)$$

where T_{eff} is expressed as a percentage (0–100%). This weights the physical burial favorability captured by MCBI by the modelled long-term preservation efficiency at each location, yielding an index that reflects both the capacity for burial and the durability of the buried carbon.

The centennial-scale index remained strongly rank-correlated with the base MCBI across all coastal grid cells (Spearman $\rho = 0.76$, $p < 0.001$, $n = 82,134$; Supplementary Fig. 8), confirming that incorporating transfer efficiency preserves the principal spatial patterns of burial favorability while additionally resolving differences in long-term preservation.

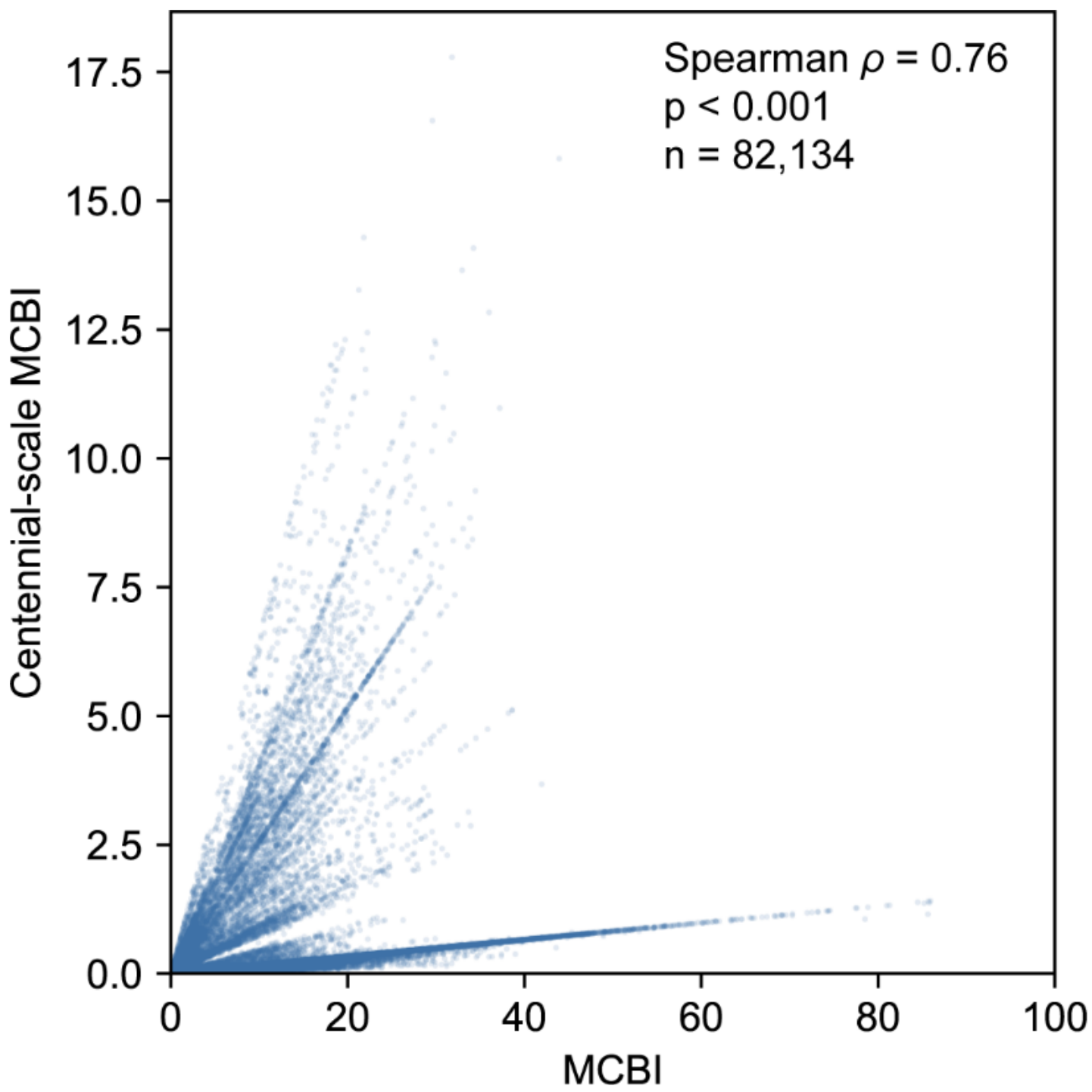

Supplementary Fig. 8 Relationship between the MCBI and the centennial-scale MCBI across all coastal grid cells (Spearman $\rho = 0.76$, $p < 0.001$, $n = 82,134$).

Reference:

1. Bradley, J.A., Hülse, D., LaRowe, D.E. & Arndt, S. Transfer efficiency of organic carbon in marine sediments. *Nat. Commun.* **13**, 7297 (2022).
